# Awakened by Cellular Stress: Isolation and Characterization of a Novel Population of Pluripotent Stem Cells Derived from Human Adipose Tissue

**DOI:** 10.1371/journal.pone.0064752

**Published:** 2013-06-05

**Authors:** Saleh Heneidi, Ariel A. Simerman, Erica Keller, Prapti Singh, Xinmin Li, Daniel A. Dumesic, Gregorio Chazenbalk

**Affiliations:** 1 Department of Pharmacology and Physiology, Georgetown University Medical Center, Washington, District of Columbia, United States of America; 2 Department of Obstetrics and Gynecology, David Geffen School of Medicine at University of Los Angeles, Los Angeles, California, United States of America; 3 Clinical Microarray Core, David Geffen School of Medicine at University of Los Angeles, Los Angeles, California, United States of America; Cedars-Sinai Medical Center, United States of America

## Abstract

Advances in stem cell therapy face major clinical limitations, particularly challenged by low rates of post-transplant cell survival. Hostile host factors of the engraftment microenvironment such as hypoxia, nutrition deprivation, pro-inflammatory cytokines, and reactive oxygen species can each contribute to unwanted differentiation or apoptosis. In this report, we describe the isolation and characterization of a new population of adipose tissue (AT) derived pluripotent stem cells, termed Multilineage Differentiating Stress-Enduring (Muse) Cells, which are isolated using severe cellular stress conditions, including long-term exposure to the proteolytic enzyme collagenase, serum deprivation, low temperatures and hypoxia. Under these conditions, a highly purified population of Muse-AT cells is isolated without the utilization of cell sorting methods. Muse-AT cells grow in suspension as cell spheres reminiscent of embryonic stem cell clusters. Muse-AT cells are positive for the pluripotency markers SSEA3, TR-1-60, Oct3/4, Nanog and Sox2, and can spontaneously differentiate into mesenchymal, endodermal and ectodermal cell lineages with an efficiency of 23%, 20% and 22%, respectively. When using specific differentiation media, differentiation efficiency is greatly enhanced in Muse-AT cells (82% for mesenchymal, 75% for endodermal and 78% for ectodermal). When compared to adipose stem cells (ASCs), microarray data indicate a substantial up-regulation of Sox2, Oct3/4, and Rex1. Muse-ATs also exhibit gene expression patterns associated with the down-regulation of genes involved in cell death and survival, embryonic development, DNA replication and repair, cell cycle and potential factors related to oncogenecity. Gene expression analysis indicates that Muse-ATs and ASCs are mesenchymal in origin; however, Muse-ATs also express numerous lymphocytic and hematopoietic genes, such as *CCR1* and *CXCL2*, encoding chemokine receptors and ligands involved in stem cell homing. Being highly resistant to severe cellular stress, Muse-AT cells have the potential to make a critical impact on the field of regenerative medicine and cell-based therapy.

## Introduction

Cellular stress is induced by abrupt disruption of the physiological niche: the optimal home most conducive to cell survival [1], [2]. Although adult stem cells have been considered an attractive source for cell therapy, their effectiveness and efficiency is hindered by a frequently low survival rate due to their exposure to a high cellular stress environment upon transplantation [3], [4], [5]. This key limitation is observed when utilizing adult stem cells for regenerative purposes, as typical cell engraftment yields are extremely low (<3%) [6]. Multiple factors contribute to this low rate of cell survival, including the harsh environment of the recipient site, harboring pro-apoptotic factors including hypoxia, malnutrition, pro-inflammatory cytokines and reactive oxygen and nitrogen species [1]. The severity of cellular stress is heightened when stem cells are administered to an acutely injured area, such as a myocardial infarction, stroke, or a peripheral ischemic injury, as are the chances of unwanted activation or differentiation of surviving cells [1], [7]. It is extremely difficult to alter the environment of the damaged tissue, which necessitates a viable alternative: to improve post-transplant stem cell survival rates through the administration of a stem cell population with the adaptations necessary for survival in the hostile host environment.

One potential solution to this problem is to gradually adapt stem cells to cellular stress prior to cell delivery. It has been shown that introducing stem cells to hypoxic conditions *in vitro* for a duration of 24–48 hours, also known as hypoxia preconditioning (HPC), provides the opportunity for these cells to adapt to low oxygen concentrations, thus increasing chances for survival upon reintroduction to hypoxic conditions *in vivo* [8]. HPC is a promising solution to the severe apoptosis that accompanies transplantation as it induces an adaptive mechanism that increases the likelihood of cell survival in a pro-apoptotic microenvironment *in vivo* [9]. Adult human mesenchymal stem cells (MSCs) and adult hematopoietic stem cells (HSCs) have similarly been shown to increase expansion, survival, and self-renewal under hypoxia conditions while maintaining the capability for multi-lineage differentiation [8], [10], [11], [12].

Another potential solution to the problem of successful delivery of stem cells to a hostile host environment is to utilize a purified population of stem cells, isolated during exposure to severe cellular stress conditions (e.g. long time incubation to proteolytic enzymes, hypoxic conditions, serum deprivation, low temperatures), for engraftment. Recently, a new stem cell population has been isolated from mesenchymal tissues such as human skin fibroblasts and bone marrow stromal cells under cellular stress conditions. These cells, termed Multilineage Differentiating Stress-Enduring (Muse) Cells, are of mesenchymal stem cell origin and comprise 1–3% of the entire cell population. Muse cells exhibit characteristics of both mesenchymal and pluripotent stem cells. They are double positive for CD105, a mesenchymal stem cell marker, and stage specific embryonic antigen-3 (SSEA3), well known for the characterization of undifferentiated human embryonic stem cells (ES) from bone marrow aspirates or from cultured mesenchymal cells such as bone marrow stromal cells and dermal fibroblasts. They express pluripotency markers including Oct3/4, Nanog and Sox2, differentiate into cells of ectodermal, endodermal, and mesodermal lineages both *in vitro* and *in vivo,* and have the ability to self-renew [13]. Advantageously, Muse cells do not appear to undergo tumorigenic proliferation, and therefore would not be prone to produce teratomas *in vivo,* nor do they induce immuno-rejection in the host upon autologous transplantation [13], [14] **.** In addition, Muse cells are shown to home into the damage site *in vivo* and spontaneously differentiate into tissue specific cells according to the microenvironment to contribute to tissue regeneration when infused into the blood stream [13]. Therefore, they exhibit the potential to make critical contributions to tissue regeneration in the absence of restrictions attributed to the difficult extraction of bone marrow stromal cells and human skin fibroblasts, and time-consuming purification methods such as cell sorting. In order to increase the viability of Muse cells as a source of tissue regeneration, a more accessible supply must be utilized.

Harvesting human adipose tissue by lipoaspiration is a safe and non-invasive procedure [15], and hundreds of millions of cells can be isolated from 1–2 liters of lipoaspirate material [16]. Therefore, adipose tissue could prove the ideal source for Muse cell isolation as opposed to bone marrow or dermis. Using lipoaspirate material, we developed a novel methodology for the isolation of a population of human Muse cells under severe cellular stress conditions (long term incubation with proteolytic enzyme, 4°C, serum deprivation, and hypoxia). Purification of human Muse cells derived from adipose tissue (Muse-ATs) does not require the use of cell sorting, magnetic beads or special devices. Muse-ATs can grow either in suspension, forming cell spheres, or as adherent cells forming cell aggregates similar to human ES cell-derived embryoid bodies as previously reported [13], [14]. Furthermore, Muse-AT cells express pluripotent stem cell markers and a variety of markers indicative of all three germlines. Upon the introduction to specific culture conditions, Muse-AT cells can differentiate to mesenchymal (adipocytes, skeletal and smooth muscle cells), endodermal (hepatocytes and biliary ducts) and ectodermal (neural cells) cell lineages both spontaneously and by differentiation induction. Immunocytochemistry and microarray data demonstrate up-regulation of the pluripotent stem cell markers Sox2, Oct3/4, and Rex1 in Muse-AT cells, as compared to previously studied multipotent adipose stem cells (ASCs). Microarray analysis reveals that Muse-AT cells highly express genes involved in cellular protection against oxidative stress. Additionally, these cells also exhibit up regulation of *CXCL2* gene expression, a critical chemokine involved in stem cell homing [17]. Muse-AT cells display down regulation of genes involved in cell death and survival, embryonic development, organism survival, cellular assembly and organization, mitosis, DNA replication, recombination and repair. Because lipoaspiration is a safe and non-invasive procedure and Muse-AT cell isolation requires a simple yet highly efficient purification technique, Muse-AT cells could provide an ideal source of pluripotent-like stem cells with the potential to have a critical impact on regenerative medicine and cell-based therapy.

## Methods

### Isolation of Muse-AT cells from Lipoaspirated Fat

Lipoaspirates (100–200 g per aspirate) were obtained from subcutaneous abdominal adipose of women undergoing elective liposuction. None of the investigators of this study had any contact with, nor any knowledge of any personal information relating to, these patients. Furthermore, human subjects were unidentifiable as well as all their characteristics and clinical records. Therefore, this study did not meet the criteria of human subjects research and HHS regulations did not apply (45 CFR 46.102(f)).

Lipoaspirate was repeatedly washed with PBS until blood was completely removed from the tissue, and then incubated with equal volume of DMEM containing collagenase (0.1%, Sigma Aldrich) for 30 min at 37°C in a shaking incubator at 110 rpm, followed by incubation in 4°C, while still in collagenase and nutritionally deficient medium (no FCS), for 16 hours under severe hypoxia conditions. Digested material was then centrifuged at 1500 rpm for 10 minutes at 4°C. Supernatant containing adipose cell debris (dead adipocytes, macrophages, red blood cells, adipose stem cells among other cell components) was removed by aspiration and the remaining cell pellets were washed several times with PBS. Pellets were re-suspended in PBS and incubated with a red blood cell lysis buffer (eBiosciences, San Diego, CA) for 10 min at R/T (2×). Remaining cell pellets containing cells highly resistant to severe cellular stress, were re-suspended in Dulbecco’s Modified Eagle Medium 1× (DMEM; CellGro, MediatechInc, Manassas, VA) comprised of 10% fetal bovine serum (FBS; Thermo Scientific Hyclone, Logan, UT) and 5% antibiotic-antimyocotic solution (CellGro, Mediatech Inc, Manassas, VA), and plated as cells in suspension as well as adherent cells. For ASC isolation, lipoaspirate material was subjected to collagenase digestion (0.1%, Sigma Aldrich) for 30 min at 37°C in a shaking incubator at 110 rpm, and ASCs were isolated and cultured as previously described [16].

### Flow Cytometry Analysis

Floating Muse-AT cells were cultured in DMEM/10% FCS for 2 days followed by FACS analysis. Cells were washed in 2% inactivate FCS/0.05% sodium Azide/PBS and were re-suspended in 100 µl of the same buffer and incubated at 4°C for 1 hour in the presence or absence of primary unconjugated rat anti-human SSEA3 (EMD Millipore; Billerica, Massachusetts). Cells were then washed twice with the same buffer and incubated with the corresponding secondary FITC-conjugated anti-rat IgM (BD Biosciences; San Diego, CA) for 45 minutes at 4°C. After two consecutive washes, cells were incubated with PE-mouse anti-human CD105 (BD Biosciences, San Diego, CA) at 4°C for 1 hour. Cells were then washed and re-suspended in 200 µl of the same buffer. Analysis of count and cell type was performed using a FACS Calibur flow cytometer and cEllQuest Pro software.

### Immunocytochemistry

Cells were fixed in 4% paraformaldehyde (20 min at R/T), washed in PBS, then incubated in 0.2% Triton for 20 min. After 2 successive washes in PBS, cells were blocked with 10% normal goat serum in 1% BSA solution for 60 min at R/T. Cells were then incubated with the primary antibodies overnight at 4°C. The following pluripotent stem cell markers were used: rat anti-human stage-specific embryonic antigen (SSEA3, Millipore, Billerica, MA), mouse anti-human octamer-binding transcription factor 3 and 4 (Oct3/4, Santa Cruz Biotech, Santa Cruz, CA), rabbit anti-human Nanog (Millipore, Billerica, MA), rabbit anti-human SRY-box 2 (Sox2, Millipore, Billerica, MA), and mouse anti-human TRA-1-60 (Abcam, Cambridge, MA); for mesenchymal cell lineages: rabbit anti-human preadipocyte factor 1 (Pref-1, [a.k.a. delta-like 1 homolog (drosophila), DLK1] preadipocyte marker, Santa Cruz Biotech, Santa Cruz, CA); mouse anti-human myosin D (MyoD, myocyte marker, R&D Systems, Minneapolis, MN), and mouse anti-human smooth muscle actin (SMA, myocyte marker, Thermo Scientific, Waltham MA); for endodermal cell lineages: mouse anti-human pan keratin (Santa Cruz, CA); rabbit anti-human α-fetoprotein (Dako, Santa Clara, CA); and mouse anti-human cytokeratin 7 (Millipore, Billerica, MA); and for ectodermal cell lineages: mouse anti-human neuron specific enolase (NSE, Millipore, Billerica, MA); rabbit anti-human glutamate receptor (Abcam, Cambridge, MA); rabbit anti-human NeuroD (Chemicon, Temecula CA); mouse anti-human nestin (Chemicon, Temecula CA); and rabbit anti-human microtubule-associated protein 2 (MAP2, AbDSerotech, Raleigh, NC). All primary antibodies were diluted 1∶200 in PBS/0.1% BSA solution. Following treatment with primary antibodies, cells were washed 3 times with PBS and incubated for 1 hour at R/T with PBS/0.1% BSA containing secondary immunofluorescent antibodies (1∶1000) Alexa Fluor 488 conjugated dye (mouse or rat, Invitrogen, Carlsbad, CA) or Texas Red conjugated dye (rabbit, Invitrogen, Carlsbad, CA). Cells were washed 4X with PBS and treated with PBS/0.2% DAPI for 10 minutes. Cells were then washed 3X with PBS. Images were acquired with an Evos immunofluorescence inverted microscope (Advanced Microscopy, Mill Creek, WA).

### Induced Differentiation of Muse-ATs

Various differentiation media were used to induce differentiation of Muse cells-AT to the three germline cell lineages. For adipocyte formation, adherent Muse-AT cells were treated with adipogenic differentiation medium containing DMEM with 0.5 mM isobutylmethylxanthine, 1 µM dexamethasone, 10 µM insulin, 200 µM indomethacin and PPAR-γ (ZenBio, Inc, Research Triangle Park, NC) over 3 or 6 days at 37°C and 5% CO_2_. Adipocytes were detected using fluorescence lipid drop marker BODIPY-C_16_ (1∶1000, Invitrogen, Carslbad, CA) following manufacturer specification. For myocyte formation, adherent Muse-AT cells were incubated in DMEM containing with 10% FBS, 5% NHS, 50µM hydrocortisone, and 1% antibiotic-antimycotic solution [16] over 3 or 6 days at 37°C and 5% CO_2_. Smooth muscle cells were identified by expression of smooth muscle actin (SMA) and skeletal muscle cells myosin D [16].

For hepatocyte and biliary cell induction, adherent Muse-AT cells were incubated in hepatocyte differentiation medium for 3 or 6 days, as previously described [18] adherent Muse-AT cells were incubated in DMEM supplemented with 10% FBS, 10 µg/ml insulin, 5.5 µg/ml transferring, 6.7 ng/ml sodium selenite (ITS; Gibco, Life Technologies, Grand Island, NY), 10 nM dexamethasone (Sigma-Aldrich, St. Louis, MO), 100 ng/ml hepatocyte growth factor (HGF, Peprotech, Rocky Hill, NJ) and 50 ng/ml and fibroblast growth factor- 4 (FGF-4, R & D Systems, Minneapolis, MN) [18] for 3 or 6 days. Hepatocytes were identified by immunohistochemistry using cytokeratin 7 and α-fetoprotein expression (see above).

For neural cell formation, Muse cells-AT were incubated as non-adherent cells in ultra-low attachment plates (Corning Incorporated, Life Sciences, Manassas, VA) in the presence of neural differentiation medium 1 containing Neurobasal medium (Gibco, Life Technology, Grand Island, NY) supplemented with B-27 supplement serum free (Gibco, Life Technology, Grand Island, NY), 100 µg/ml kanamycin (Gibco, Life Technology, Grand Island, NY), 2 mM glutamine (Sigma-Aldrich, St. Louis, MO), 30 ng/ml bFGF (Peprotech, Rocky Hill, NJ) and 30 ng/ml EGF (Peprotech, Rocky Hill, NJ) for 7 days [19]. Cells were then transferred to polystyrene culture slides (BD Biosciences, San Jose, CA) and cultured for another 7 days as adherent cells in the presence of neural differentiation medium 2 containing 1 DMEM supplemented with 2% FCS, 25 ng/ml bFGF and 25 ng/ml BDNF (Peprotech, Rocky Hill, NJ) [19]. Neural cells were identified by immunohistochemistry using nestin and MAP2 as indicated above.

### Microarray Analysis

Muse-AT cells and ASCs were isolated from lipoaspirate material of three different patients. RNA was extracted using an RNeasy Mini Kit (Qiagen) and analyzed by Hokkaido System Science Co. Ltd. Array signals were processed and normalized using the GeneSpring GX version 12.1.0 (Agilent Technologies). Data has been deposited into the Gene Expression Omnibus databank with the access number GSE46353. The criteria for selecting differentially-expressed genes were preset as at least 2-fold difference in either direction plus statistical significance (*P*<0.05, unpaired *t* test). Microarray analysis was performed using the software program IPA via a license to Ingenuity (https://analysis.ingenuity.com/pa/login/login.jsp) to identify (1) functional pathways (cell function, physiological function, diseases), (2) canonical signaling pathways (3) networks of related genes derived from genes changed in the analyzed comparisons and (4) upstream regulators. Further information regarding gene function was obtained from the program GeneDecks V3 at www.genecards.org [20]. Statistical analyses were carried out by Fischer’s exact test (as performed automatically by the software). In determining which genes are only expressed in either Muse-ATs or ASCs, all samples, having been performed in triplicate, had to display uniform detection (indicated with at least 100 standard units) or absence (at most 30 standard units) along with a P-value <0.05.

## Results

### Muse-ATs Isolated from Lipoaspirated Human Adipose Tissue

Adipose tissue is composed of adipocytes (mature cells) and the stromal vascular fraction (SVF) containing a heterogeneous population of cells, including adipose tissue macrophages (ATMs), adipose stem cells (ASCs), mesenchymal stem cells, and fibroblasts [15], [16].

**Figure 1:**
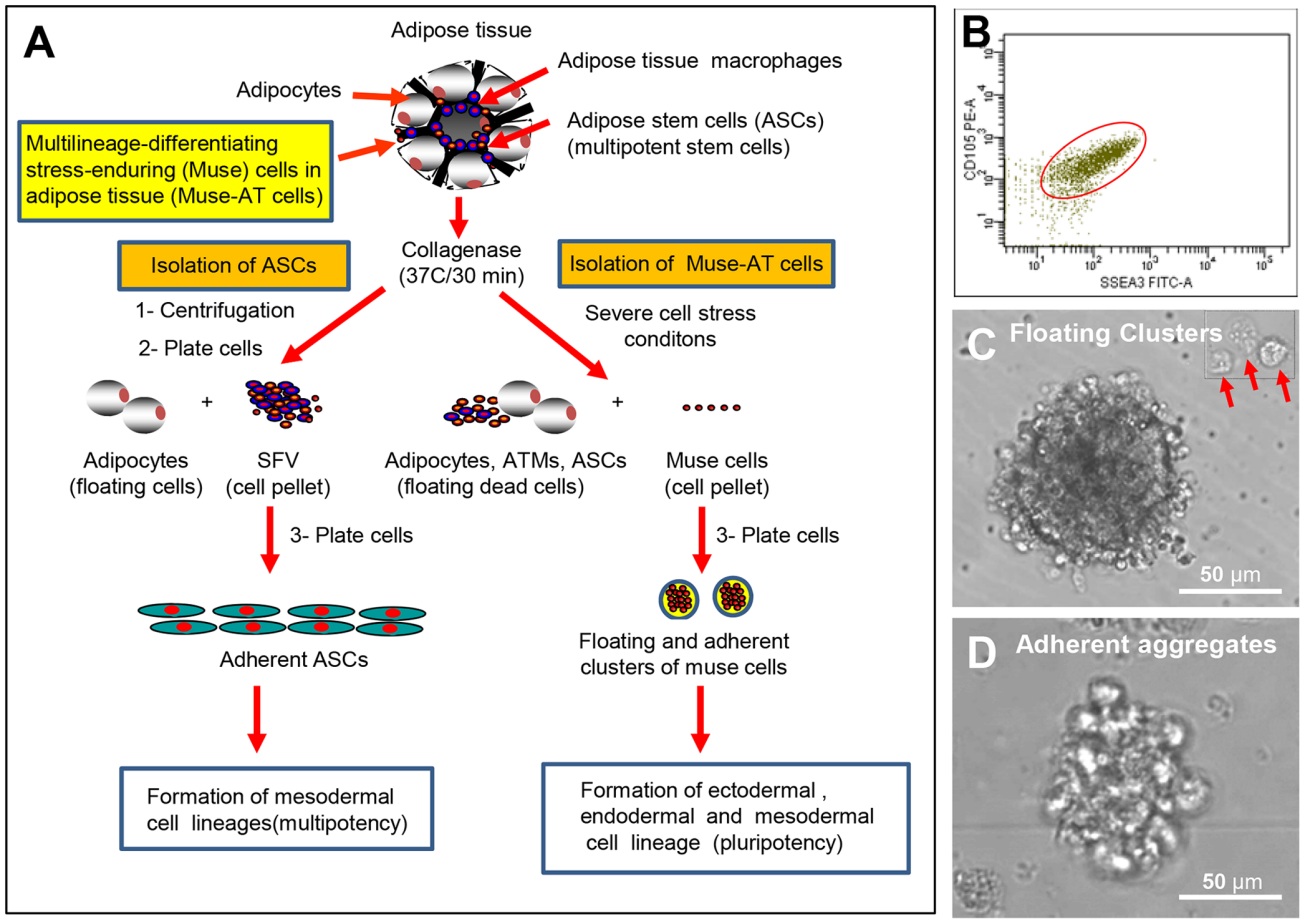
Isolation and morphologic characterization of Muse-ATs. (**A**) Schematic of Muse-AT isolation and activation from their quiescent state by exposure to cellular stress. Muse-AT cells were obtained after 16 hours, with incubation with collagenase in DMEM medium without FCS at 4°C under very low O_2_ (**See Methods**). (**B**) FACS analysis demonstrates that 90% of isolated cells are both SSEA3 and CD105 positive. (**C**) Muse-AT cells can grow in suspension, forming spheres or cell clusters as well as individual cells (see red arrows) or (**D)** Muse-AT cells can adhere to the dish and form cell aggregates. Under both conditions, individual Muse-AT cells reached a diameter of approximately 10µm and cell clusters reached a diameter of up to 50µm, correlating to stem cell proliferative size capacity.

We explored the possibility of both activating and isolating Muse-AT cells from their quiescent state by exposing them to cellular stress **(** **Fig. 1A**). Lipoaspirated material was first incubated in collagenase for 30 min at 37°C to release adipocytes (floating cells) and different cellular components present in the SVF as previously described [16]. This material was then subjected to severe cellular stress, including long incubation with collagenase, low temperatures, low serum and hypoxia, to kill fragile adipose cells and release Muse-AT cells. Optimal conditions for the release of Muse-AT cells were determined to be 16 hours incubation with collagenase in DMEM medium without FCS at 4°C under very low O_2_, which subsequently gave way to a homogenous population of Muse-AT cells. Approximately 90% of isolated cells were both SSEA3 and CD105 positive, as determined by flow cytometry **(** **Fig. 1B** **)**. This high purity is presumably due to the severity of the cellular stress conditions, responsible for the depletion of other cell types. As all other components of the adipose tissue lipoaspirate failed to survive, a population of highly purified Muse-AT cells was obtained, and therefore further purification processes were not necessary. Muse-AT cells were plated in both adherent and non-adherent cell culture dishes. We observed that Muse-AT cells can grow either in suspension or in adherence culture to form the characteristic cell clusters observed in ES cell-derived embryoid body, as described in bone marrow and dermal fibroblast-derived Muse cells in previous reports **(** **Fig. 1C, D** **)** [13], [14]. Under both conditions, individual Muse-AT cells reached a diameter of around 10µm and cell clusters reached a diameter of up to 50µm by day 3 **(** **Fig. 1C–D** **)**, which has been previously demonstrated to mark the limit of their proliferative capacity [**13**].

### Muse-ATs Spontaneously Express Pluripotent Stem Cell Markers

**Figure 2:**
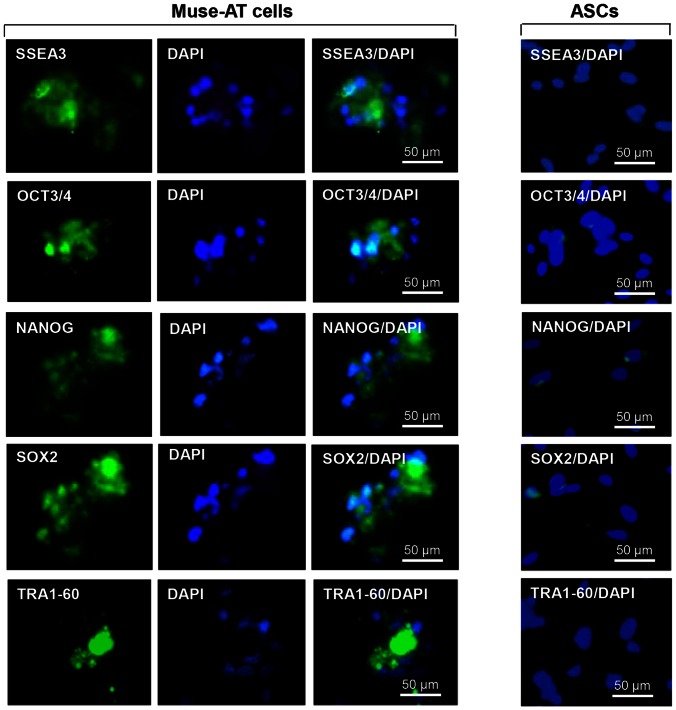
Muse-ATs express pluripotent stem cell markers. Immunofluorescence microscopy demonstrates that Muse-AT aggregates, along with individual Muse-AT cells, express characteristic pluripotent stem cell markers, including SSEA3, Oct3/4, Nanog, Sox2, and TRA1-60. Comparatively, ASCs (right panel) derived from the same lipoaspirate under standard conditions (see above, [16] were negative for these pluripotent stem cell markers. Nuclei were stained with DAPI (blue). Original magnification, 600 X.

Upon transfer and adherence to chamber slides for immunofluorescent staining, both the Muse-AT cell clusters and individual Muse-AT cells strongly expressed all of the characteristic pluripotent stem cell markers that were examined. These included SSEA3, a cell-surface glycosphingolipid frequently used to detect human ES cells and to purify Muse cells from bone marrow and dermis [13]; Oct3/4 a protein involved in the self-renewal of human ES cells; Nanog, a transcription factor involved in the self-renewal of human ES cells; Sox2, a transcription factor that controls genes involved in embryonic development; and TRA-1-60, which reacts with the antigen TRA-1-60 on the surface of embryonic germ cells and ES cells **(** **Fig. 2** **)**. Comparatively, ASCs derived from the same lipoaspirated tissue were either negative or weakly positive for these pluripotent stem cell markers **(** **Fig. 2** **)**.

### Mesodermal Differentiation of Muse-ATs

**Figure 3:**
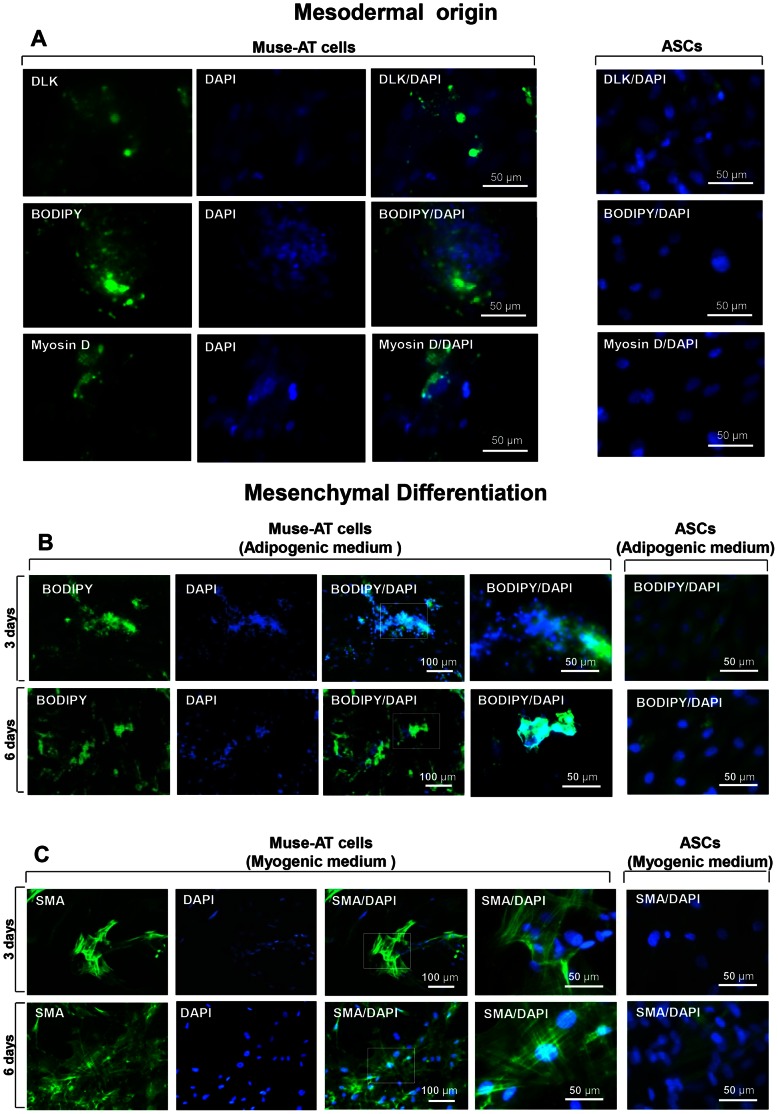
Muse-AT cells can differentiate to mesodermal cell lineages. Isolated Muse-ATs were grown as adherent cells in the presence of DMEM/10%FCS for 6 days. (**A**) Immunostaining indicates that Muse-AT have the capacity to spontaneously differentiate to mesodermal cell lineages. DLK is a specific marker for preadipocytes; BODIPY-C_16_ recognized lipid drops present in adipocytes; Myosin D is specific marker for myocytes. Comparatively, ASCs (right panel) which are mesenchymal stem cells derived from the same lipoaspirate under standard conditions (see above, [16] were slightly positive for DLK; nuclei were stained with DAPI (blue), original magnification, 600 X. (**B**) Isolated Muse-AT cells were grown as adherent cells in the presence of adipocyte differentiation medium **(See Methods**) for 3 or 6 days and formation of adipocytes was detected using BODIPY-C_16_; (**C**) Isolated Muse-AT cells were grown as adherent cells in the presence of myocyte differentiation medium and formation of myocytes was detected using anti human-MSA antibodies. Comparatively, ASCs (right panel) which are mesenchymal stem cells derived from the same lipoaspirate under standard conditions (See above, [16] were slightly positive for BODIPY-C_16_ or MSA **(B and C, right panel)**. Nuclei were stained with DAPI (blue). Original magnification was 200 X (first three rows) or 600 X (last two rows).

Adipose tissue has been shown to harbor ASCs with the ability to differentiate into the mesenchymal cell lineages: adipocytes, chondrocytes, myocytes and osteoblasts [16], [20]. However, ASCs in the appropriate differentiation medium require approximately 2 ½ weeks to develop mature adipocytes, and myocyte differentiation, with characteristically fused nuclei, takes approximately 3 weeks [16]. To determine the potential of Muse-AT cells to spontaneously differentiate into cells of mesodermal lineage, Muse-AT cells were grown as adherent cells in culture medium only containing DMEM, 10% FCS+Antibiotics for 3 days. Spontaneous differentiation of Muse-AT cells into a mesodermal lineage was determined by immunocytochemistry. Mesodermal markers included DLK, a marker for preadipocytes [21], BODIPY-C_16_, a fluorescent dye used to detect lipid accumulation [22], and myosin D (Heavy Chain), a marker for the heavy chain portion of the Myosin II protein found in skeletal muscle cells [16], [23], [24]. After cultured as adherent cells for 3 days, Muse-AT cells displayed significant expression of DLK, (21±8% of all DAPI-positive cells), BODIPY-C_16_ (61±13% of all DAPI-positive cells) and Myosin D (25±4% of all DAPI-positive cells), as compared to ASCs, which were slightly positive only in response to DLK (17±7% of all DAPI-positive cells) **(** **Fig. 3A** **)**. In the presence of adipogenic medium, demonstrated over the course of 3 and 6 days, Muse-AT cells accumulated considerable concentrations of lipid drops indicated by the formation of BODIPY-C_16_ (+) droplets, which characterized 80±4% (3d) and 83±3% (6d) of all DAPI-positive cells **(** **Fig. 3B** **)**. In contrast, ASCs showed a weak yet detectable signal for BODIPY-C_16_ (+) due to the presence of lipid accumulated in the cytoplasm as a result of ASC commitment to the preadipocyte cell fate **(** **Fig. 3B** **)**. At 3 days that there is a stark morphological difference between Muse-AT cells and ASCs, perhaps most apparent in the smaller size of Muse-ATs, which is very evident in the nucleus size, as indicated by DAPI. However, this morphology is actually much more similar at day 6, at which point the nucleus size has grown significantly in Muse-ATs, and is roughly the same as ASCs. BODIPY labeling of lipids with the intensity observed in the Muse-ATs is typically observed in ASCs after 2–3 weeks in culture [25], [26]. Predictably, ASCs fully differentiated to adipocytes after 17 days of incubation in adipogenic medium (data not shown, [16]).

In the presence of myogenic medium for 3 and 6 days, Muse-AT cells differentiated into smooth muscle cells, with the characteristic morphology of smooth muscle fibers and strong expression of SMA that characterized 77±3% (3 d ) and 83±4% (6 d) of all DAPI-positive cells **(** **Fig. 3C** **)**. Under the same culture conditions, ASCs were only slightly positive after 6 days of incubation (25±4% of all DAPI-positive cells) **(** **Fig. 3C** **)**. Differentiation of ASCs to myocytes required ASCs exposure to myogenic medium for at least 21 days (data not shown, [16]). These results demonstrate that while both types of adipose-derived stem/progenitor cells have the capacity to differentiate, activated Muse-AT cells differentiate towards both adipocyte and myocyte lineages much more quickly than ASCs.

### Endodermal Differentiation of Muse-ATs

**Figure 4:**
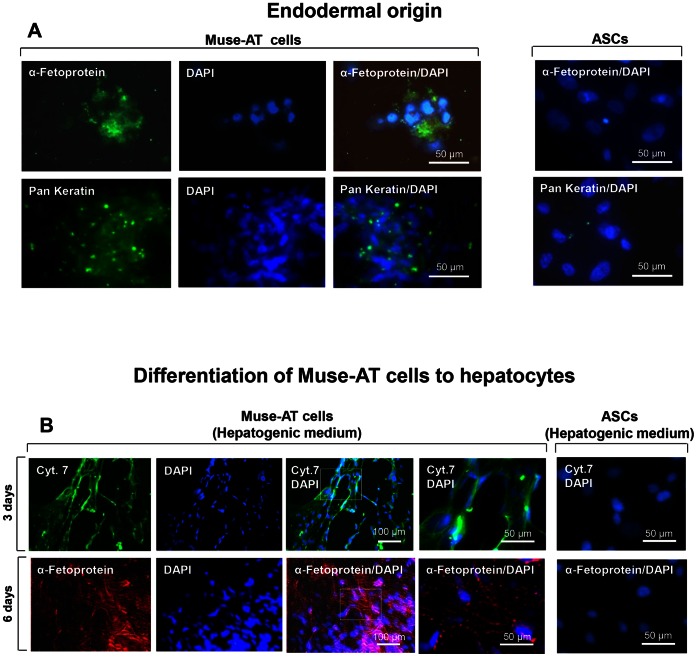
Muse-ATs can differentiate to endodermal cell lineages. Isolated Muse-ATs were grown as adherent cells in the presence of DMEM/10%FCS for 6 days. (**A**) Immunostaining indicates that Muse-AT have the capacity to spontaneously differentiate to endodermal cell lineages. α-fetoprotein and pan-keratin are specific markers for hepatocytes; Nuclei were stained with DAPI (blue). Original magnification, 600 X. Comparatively, ASCs (right panel) derived from the same lipoaspirate under standard conditions (see above, [16]) were negative for α-fetoprotein and pan-keratin. Nuclei were stained with DAPI (blue); original magnification, 600 X; (**B**) Isolated Muse-AT cells were grown as adherent cells in the presence of hepatocyte differentiation medium **(see methods)** for 3 or 6 days and formation of hepatocytes was detected using anti-human cytokeratin 7 or α-fetoprotein antibodies. Comparatively, ASCs (right panel) were completely negative for cytokeratin 7 and α-fetoprotein **(B, right panel)**. Nuclei were stained with DAPI (blue). Original magnification was 200 X (first three rows) or 600 X (last two rows).

Spontaneous differentiation of Muse-AT cells to an endodermal lineage (hepatocytes) was detected in Muse-AT cells cultured in DMEM/10% FCS for 3 days. Muse-AT cells were recognized by α-fetoprotein (19±7% of all DAPI-positive cells), which is expressed during the development of endoderm and progenitors of hepatocytes [27] and pan keratin (21±8% of all DAPI-positive cells), a marker for filaments characteristic of biliary tract epithelial cells [27] **(** **Fig. 4A** **)**. In the cluster of cells, α-fetoprotein strongly recognized fatty acids in dimeric and trimeric forms localized in both the cytoplasm and plasma membrane of Muse-AT cells **(** **Fig. 4A** **)**, as was previously described in human hepatoblastoma cell line HepG2 [28]. In contrast, ASCs were negative for these endodermal cell markers **(** **Fig. 4A** **)**. Muse-AT cells previously incubated in hepatogenic differentiation medium for 3 and 6 days were positive for cytokeratin 7, an intermediate filament protein in biliary cells that characterized 69±2% (3 d) and 80±7% (6 d) of all DAPI-positive cells, as well as for α-fetoprotein which recognized 90±4% (3 d) and 91±5% (6 d) of all DAPI-positive cells (**Fig. 4B** **)**, while ASCs were negative **(** **Fig. 4B** **)**. These results demonstrate that Muse-AT differentiation mirrors previous studies of pluripotent stem cells differentiation to hepatocytes in terms of both time in culture (3 days) and differentiation efficiency [29], [30].

### Ectodermal Differentiation of Muse-ATs

**Figure 5:**
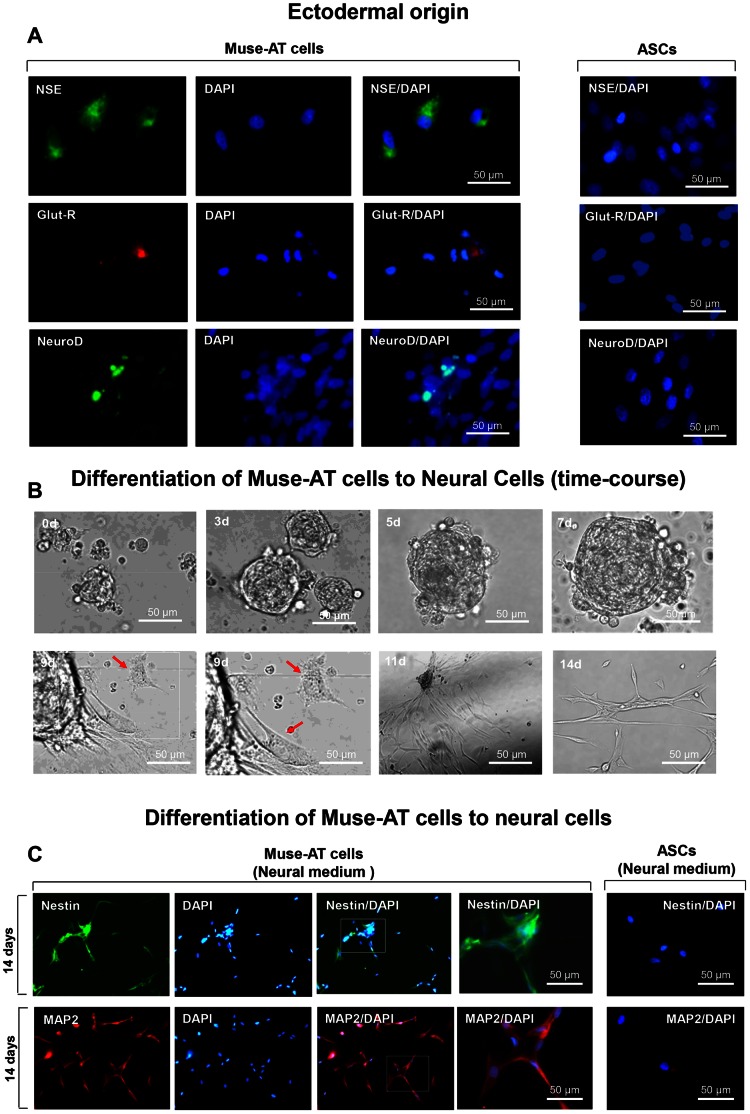
Muse- ATs can differentiate to ectodermal cell lineages. (**A**) Isolated Muse-ATs were grown as adherent cells in the presence of DMEM/10%FCS for 6 days. Immunostaining indicates that Muse-AT cells have the capacity to spontaneously differentiate to ectodermal cell lineages. NSE, Glu-R and Neuro D are specific markers for neural progenitor or neural-like cells. Comparatively, ASCs (right panel) were negative for NSE, Glu-R and NeuroD. Nuclei were stained with DAPI (blue); original magnification was 600X. (**B**) Isolated Muse-AT cells were grown as non-adherent cells in the presence of Neurobasal medium/B-27 supplement serum free/kanamycin/glutamine/bFGF and EGF for 7 days. Formation of neural cell spheres was detected at different times during this incubation. Cells were then grown as adherent cells for an additional 7 days in a DMEM 2% FCS/bFGF/BDNF. Formation of neural-like cells is indicated by red arrows. (**C**) Isolated Muse-AT cells were grown for 7 days as non-adherent cells and then cultured for another 7 days as adherent cells **(See Methods)**. Neural-like cells were detected by immunofluorescence using anti-human nestin and MAP2 antibodies. Comparatively, ASCs (right panel) were negative for nestin and MAP2 (**B, right panel)**. Nuclei were stained with DAPI (blue). Original magnification was 200 X (first three rows) or 600X (last two rows).

To complete our examination of the potential for spontaneous differentiation into the three germlines, Muse-AT cells were cultured for 3 days in DMEM/10%FCS with antibodies characterized by ectodermal cell markers including neuron-specific enolase (NSE), a marker used to detect neocortical neuron progenitors [31], [32], metabrotopic-glutamate receptor (Glut-R), a marker used to detect microglial and neural like cells [33], [34] and NeuroD, a marker used to detect neocortical precursor cells [35], [36]. Again, Muse-AT cells showed significant expression of all these markers with 30±5% (Glut-R), and 15±5% (NeuroD) of all DAPI-positive cells **(** **Fig. 5A** **)**, confirming their potential to spontaneously differentiate into ectodermal cells, as opposed to control ASCs **(** **Fig. 5A** **)**. We monitored the morphological progression of Muse-AT cells into neurons throughout the incubations in both the first and second neurogenic differential mediums **(see methods)** following similar protocols previously used for the differentiation of ES and iPS cells into cells of neural origin [37], [38], [39], [40], [41]. Muse-AT cells exhibited a progression from the formation of large cell spheres with finger-like projections to long, neuron-like cells, which subsequently formed large networks **(** **Fig. 5B**) [37]. Muse-AT cells cultured in suspension for 7 days in neural differentiation medium 1 (**see methods**) progressively form large cell clusters. Subsequent treatment as adherent cells for 7 days in neural differentiation medium 2 (**see methods**) induced the formation of neuron-like cells detectable by morphology and by axon and dendrite-specific markers.

Immunocytochemistry studies on Muse-AT cells having previously undergone the two-tiered neurogenic differentiation revealed expression of both nestin, a stimulant of survival, renewal and proliferation of neural progenitor cells [42] which characterized 65±11% of all DAPI-positive cells and MAP2, a protein involved in the polymerization of microtubules [43] which recognized 92±2% of all DAPI-positive cells **(** **Fig. 5C** **)**. ASCs were negative for nestin, while MAP2 exhibited a minimal degree of non-specificity inherent to the marker (5% of all DAPI-positive cells) **(** **Fig. 5C** **)**.

### Microarray Data

To explore Muse-AT cell gene expression, microarrays were preformed and expression differences between Muse-AT cells and APCs were analyzed. Differential expression of at least a 2-fold change between Muse–AT cells versus ASCs was observed in 435 up and 434 and down-regulated genes respectively (p<0.05, **Tables S1–S2**). Of these, 99 genes were expressed in all Muse-AT samples and absent in all ASC samples. Genes expressed only in Muse-ATs included *TNFSF14* (p<0.0002), *IL3RA* (p<0.0007), *CSF3* (p<0.0013), *IL10RA* (p<0.004), *GATA2* (p<0.005), and *BMP7* (p<0.02) **(Table S3)**. Interestingly, Muse-ATs expressed numerous CD-markers that ASCs did not, while no CD-markers were unique to only the ASCs **(Table S4)**.

ASCs expressed 41 genes that Muse-ATs did not **(Table S5)**. These genes were largely related to mitosis and cell cycling, and included *ESCO2* (p<0.0007), *KIF20A* (p<0.0009), *CENPF* (p<0.0023), *NEK2* (p<0.0029), *RAB3B* (p<0.0031), and *FGF5* (p<0.0068).

**Figure 6:**
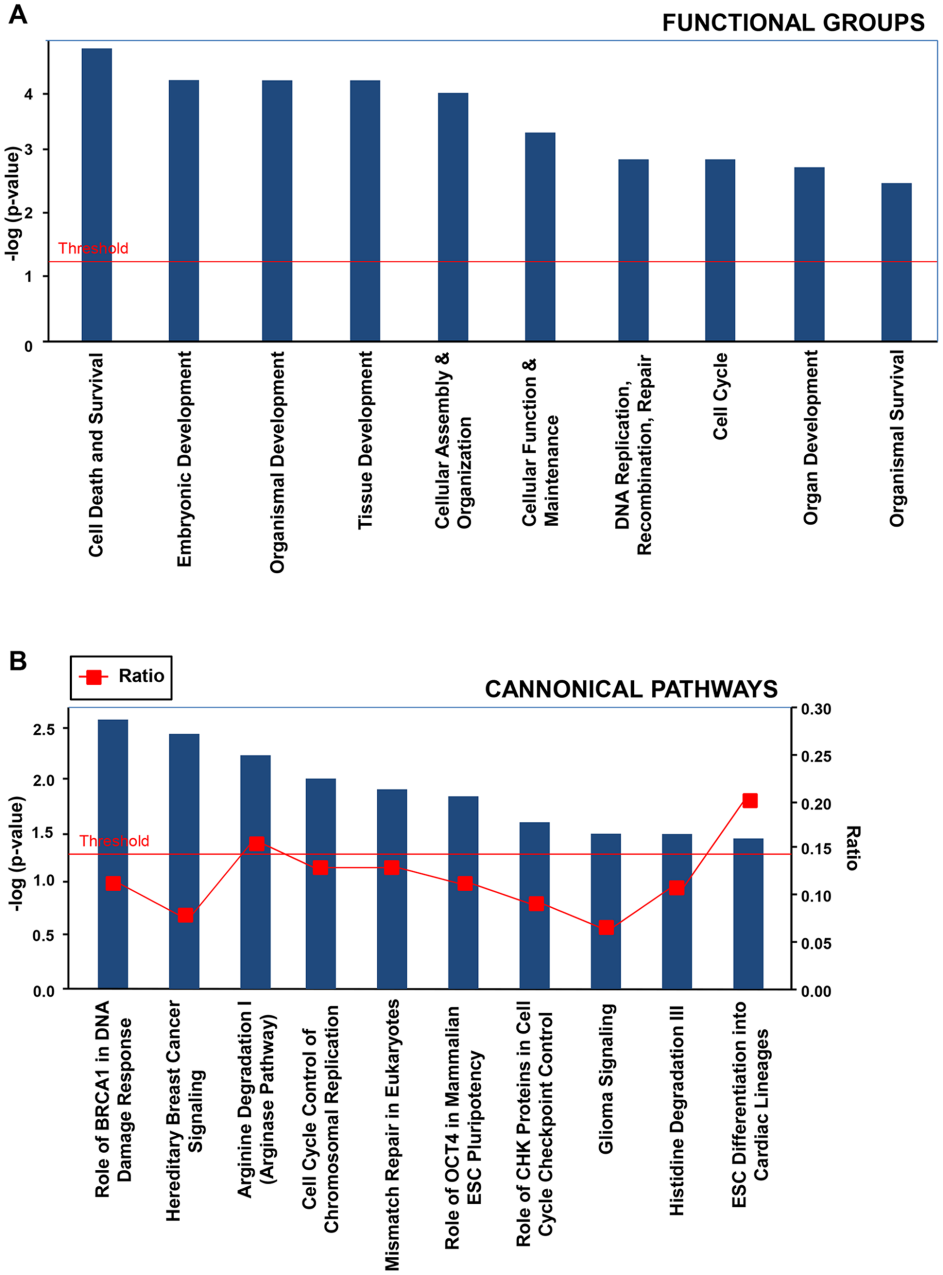
Identification of functional groups and canonical pathways in Muse-ATs vs ASCs. Identification of the top ten (**A**) functional groups (**B**) canonical pathways of all differentially expressed genes (2 fold or higher) in Muse-AT cells versus ASCs. Fischer’s exact test was used to calculate a p-value determining the probability of the association between the genes in the data set with functional groups and canonical pathways. Both (**A**) functional groups (**B**) canonical pathways are displayed along the x-axis, while the y-axis displays logarithm of p values calculated by Fisher exact between the ratio of the number of genes differentially expressed genes (2 fold or higher) in Muse-AT versus ASCs in a given functional group or pathway divided by total number of genes that make up that functional group or pathway with a threshold for statistical significance set at 0.05. The analysis was performed by Ingenuity Pathways analysis software.

Gene ontology analysis was performed, and observed differential expression in Muse-AT cells correlated strongly to categories of cellular functions, the most statistically significant being: cell death and survival (p = 2.04E-05 to 3.15E-02), embryonic development (p = 5.92E-05 to 3.15E-02), tissue development (p = 5.92E-05 to 3.15E-02), cellular assembly and organization (p = 1.07E-04 to 3.15E-02), cellular function and maintenance (p = 4.04E-04 to 3.15E-02), DNA replication, recombination and repair (p = 1E-0.3 to 3.15E-0.2), cell cycle (p = 1.12E-0.3 to 3.15E-0.2), organ development (p = 1.54E-0.3 to 3.15E-0.2) and organismal survival (p = 2.63E-0.3 to 2.63E-0.3) **(** **Fig. 6A** **, Tables S1–S2)**.

The most predominant up-regulated genes of Muse-AT cells versus ASCs included *CXCL2* (777.8 fold), *ESCM2* (153.2 fold) *DLL1* (147.4 fold), *NR4A2* (139.2 fold), *ADAMTS9* (115.3 fold), *BMX* (91.5 fold), *MYZAP* (87.6 fold), *ALDH1A2* (47.1 fold) and *SOD2* (41.4 fold), indicating that these genes were otherwise turned off or suppressed in ASCs **(Table S1)**. The most predominant down-regulated genes included *AK5* (136.6 fold), *GREM2* (115.2 fold), *CEP55* (93.6 fold), *BUB1B* (66.4 fold), *CDCA3* (62.5 fold), *NUF2* (54.8 fold) and *DEPDC1* (52.7 fold) **(Table S2)**.

The most highly expressed canonical pathways include the role of Oct4 in embryonic stem cell pluripotency (*SOX2, NR6A1, BRCA1, ASH2L, POU5F1), BRCA1* in DNA damage and hereditary breast cancer signaling *(POLRJ2/POLR2J3, FANCB, POLR2J, CDK6, RPA1, PIK3R2, RFC5, BLM, BRCA1, RFC3),* cell cycle control of chromosomal replication *(MCM6, ORC3, CDK6, RPA1),* DNA repair *(RPA1, RFC5, RFC3),* arginine degradation *(ALDH4A1, OAT),* and embryonic stem cell differentiation into cardiac lineages *(SOX2, POU5DF1*) **(** **Fig. 6B** **)**. These data provide further insight into the potential role Muse-AT cells in DNA repair, cell cycle, oxidative stress, cancer cell regulation, as well as their intrinsic pluripotency.

Up and down-regulation of critical genes involved in cell death and survival (e.g. *SGK1* (up1.6x), *MDH1*, *ATF2*, *HSPA8*, *PDIA3*, *BRD1*, *CALM1*, *NR4A2*, *GATA2*, *CDK6*, *NUF2*, *CDK6*, *BRC1*, *BUB1B* and *CCXL2*) could contribute to Muse–AT cell resistance to severe cellular stress exposure. The *BRC1* DNA damage and repair pathway [42] is down-regulated in Muse-AT cells versus ASCs, indicating the high capacity of Muse-AT cells to resist DNA damage as a result of severe cellular stress. We detected 14 individual genes related to DNA repair to be up-regulated in Muse-ATs (**Table S6A**). Additionally, eight ABC-cassette genes were more highly expressed in Muse-ATs. (**Table S6B**). Finally, in order to examine methods of cell communication, we analyzed the expression of gap junction related genes, and observed that Muse-ATs expressed three connexin genes *GJA4*, *GJB2*, *GJB4,* as well *as C1orf71* (*CNST*), which encodes the recently described connexin recycling protein, consortin [44] (**Table S6C)**.

## Discussion

Most studies utilizing adult stem cells for regenerative purposes have yielded disappointing results regarding post-transplant stem cell survival, attributed to a hostile host environment at the recipient site of injury, as seen in myocardial infarction, stroke, and peripheral ischemic wound [3], [4], [5], [6]. Despite survival enhancement via hypoxic preconditioning under *in vitro* ischemic environments [8], [9], [10], [11], [12], the *in vivo* effect of HPC on adult stem cells used for tissue engraftment is still controversial and more studies are required to optimize the use of HPC as a stimulus for various stem cell functions before their use in clinical trials [11], [45]. Recent studies in a flap ischemic rat model treated with ASCs having undergone HPC, showed a slight improvement in tissue engraftment and chemotaxis in comparison to the effects produced by untreated ASCs [46]. Therefore, the availability of a new stem cell population resistant to cellular stress could offer an attractive advance in the realm of post-transplant stem cell survival. In the present study, we described a novel population of pluripotent-like stem cells isolated from adipose tissue, termed Muse-AT cells, which are highly resistant to severe cellular stress and could emerge as the optimal population of cells to utilize in regenerative stem cell therapy.

Muse cells, comprising only a small population of MSCs in bone marrow (0.8–1%) and fibroblasts (2–3%), exist in a dormant, or quiescent state under normal physiological circumstances within the cellular niche [13]. This quiescent fate is considered characteristic of multiple adult stem cell lineages, including hematopoietic stem cells and epithelial stem cells, which have been shown to play a role in the preservation of the capacity to self-renew [47], [48]. Quiescence is internally regulated by reactive oxygen species for cell maintenance and HIF-1α to promote survival under hypoxic conditions, among other contributing factors, and externally regulated by environmental factors including transforming growth factors (TGFs) and bone morphogenic proteins (BMPs) within the physiological niche [48], [49], [50]. Quiescence also plays a critical role in the perseverance of malignant cancer stem cells and subsequent cancer relapse despite the action of anti-cancer treatments [48]. In order to activate quiescent stem cells, the physiological niche must undergo a disruption that induces cellular stress.

Chemokines and their receptors play a critical role during this process. *CXCL2*, a critical chemokine involved in stem cell homing, has been found to play a role in the mobilization of previously quiescent stem cells from their dormant state in the physiological niche [48]. CXCL2 is often overexpressed in cancer cells, and this expression is believed to contribute both to survival and to the pervasiveness of some cancers [51]. In the heart, CXCL2 expression rises in response to myocardial infarction, and studies have shown that pre-conditioning of MSCs with CXCL2 increases post-transplant stem cell survival rates [52]. High *CXCL2* gene expression in Muse-AT cells (>770 fold increased in Muse-AT versus ASCs) could therefore explain the intrinsic genetic resistance of Muse-AT cells to cellular stress [13], as well as their capacity for resilience through their quiescent residence in the physiological niche. Additional studies would be warranted to determine if the differential chemokine expression observed in Muse-AT cells might be able to alter the homing of donor cells to areas of tissue injury or stress.

Muse-AT cells express pluripotent stem cell markers and can spontaneously differentiate into mesenchymal (adipocytes, smooth and skeletal muscle cells), endodermal (hepatocytes and biliary cells) and ectodermal (neural cells) cell lineages with an efficiency of approximately 23, 20 and 22% respectively. Interestingly, Muse-AT cells preferentially differentiate spontaneously into adipocytes (61%), suggesting that Muse-AT cells have an epigenetic memory of their tissue of origin. It may be possible that adipose tissue acts as a natural reservoir for the cells, and without stress Muse-ATs may remain in a dormant state. Similarly, iPS cells possess an epigenetic memory, which to date has precluded entirely successful reprogramming, and restricting those cells to a cell fate within the same lineage as the original stem cell source [53]. Muse-AT cells also expressed higher levels of DLK, or Pref-1 (Preadipocyte factor-1), than ASCs. This marker is expressed in preadipocytes and recently differentiated adipocytes [54]. Overall, portions of the dish that are strongly DLK positive have morphologies that are similar to ASCs, with a much stronger label.

Adipose tissue has been shown to harbor ASCs with the ability to differentiate into the mesenchymal cell lineages: adipocytes, chondrocytes, myocytes and osteoblasts in the appropriate differentiation medium [16], [20]. Notably, Muse-AT cells cultured in adipogenic medium exhibit adipocyte identifiers, with visibly detectable lipid drop content, and differentiation progressing substantially by day 6. However, ASCs require approximately 2½ weeks to develop into mature adipocytes [16]. Incubation of Muse-AT cells in myogenic medium exhibits significant differentiation by day 3. In contrast, ASC differentiation into myocytes, characterized by fused nuclei, requires approximately three weeks of incubation in myogenic culture media [16]. Furthermore, the morphological differentiation by day 6 correlates to the ASC differentiation that would normally be observed after approximately 3 weeks in culture. Taking this into account, the abundant availability of lipoaspirate material and the temporal efficiency of Muse-AT cells in induced differentiation is highly advantageous when considering their potential use for stem cell therapy in the generation of soft tissue for reconstructive surgery as well as for muscle regeneration and repair.

Muse-AT cells exhibit high potential as a regenerative treatment for injuries that require regeneration of cardiac muscle (mesenchymal origin) in response to myocardial infarction, in which high rates of post-transplant stem cell survival as well as highly efficient differential capacity are required. Additionally, in cases of ischemia or acute injury, a hypoxic and inflammatory environment with pro-inflammatory cytokines exposes therapeutic stem cells to similar inflammatory stress, which can result in unintended stem cell differentiation to fibroblasts and myofibroblasts, which in the case of cardiomyocytes, results in both scar tissue formation and an unintended reduction in action potential conductivity [55].

Exposing Muse-AT cells to the same culture conditions used to induce ES and iPS hepatogenesis results in cytokeratin 7 and α-fetoprotein positive hepatocyte-like cells [29], [30], [56], [57], [58], [59], [60], [61]. Similarly, Muse-AT cells differentiate into neural-like cells in a comparable manner previously described with regards to ES and iPS cells [38], [39], [40], [41]. Muse-AT cells cultured in suspension for 7 days progressively form large cell clusters **(** **Fig. 5B** **)**. Subsequent treatment as adherent cells for 7 days attribute to the formation neuron-like cells detectable by morphology and by axon and dendrite-specific markers MAP2 [37] and by nestin [62] **(** **Fig. 5C** **)**. These results indicate the potential use of Muse-AT cells for liver and neural regeneration that parallel that of both ES and iPS cells. However, as these cells are not transgenically induced, they may be less worrisome in regards to the issue of teratogenesis.

Our microarray data confirmed that Muse-AT cells over-express the pluripotent stem cell markers *SOX2*, *OCT3/4,* (*POU5F1*) and *REX1* 3–4 fold in comparison with ASCs, indicating the intrinsic pluripotent and differential capacity of Muse-AT cells. Concordantly, Muse-AT cells exhibit up-regulation of genes associated with embryonic development. Up-regulation levels of pluripotent stem cell markers were observed in Muse cells derived from fibroblasts and bone marrow cells [13], although level of expression of these pluripotent stem cell markers in Muse-AT cells were very low compared with hESCs or iPS cells generated from human fibroblasts [14]. Muse-AT cells have a relatively low expression of many genes involved in tissue development, cellular assembly and organization, cellular function and maintenance, DNA replication, repair, and cell cycling in comparison with ASCs. These results suggest an intrinsic non-tumorigenic capacity of Muse-AT cells, in accordance with previously published data of the regenerative properties of Muse cells in the absence of the production of teratomas upon transplantation *in vivo* [13], [14]. However, under abnormal stress conditions (e.g. programming Muse-AT cells with the Yamanaka’s factors), it may be possible to activate endogenous Muse cells, which could account for the small population of cells that are converted into iPS cells [14]. Such a theory is supported by previous studies regarding the possible role of adult organ-specific positive Oct4 (+) stem cells during asymmetric division in the generation of cancer cells [63].

There are 144 differentially expressed genes associated with cell death and survival in Muse-AT cells versus ASCs, indicating that significant changes in expression of many of these genes could be required for the adaptive transition from the quiescent to the active state of Muse-AT cells during severe cellular stress. For example, Muse-AT cells over-express *ALDH1A2* (47 fold change versus ASCs) and *SOD2* (41 fold change versus ASCs) which have an anti-oxidative stress and anti-apoptotic functions [52], [64].

Many genes related to DNA repair are up-regulated in Muse-ATs, indicating a potentially high capacity to resist DNA damage as a result of severe cellular stress. Furthermore, several ABC-cassette genes were differentially expressed in Muse-ATs, indicating that active expression of drug transporter genes may play a role in observed stress resistance. The connexin gene family encodes proteins responsible for gap junction formation vital for direct cell-to-cell communication [65]. Epithelial derived pluripotent stem cells from human kidney and breast down regulate gap junctions formation to maintain a capacity for self renewal, which may be a necessary mechanism for other adult stem cells to retain pluripotent potential [63], [65]. The recently described protein consortin plays a critical role in gap junction recycling and degradation [44]. Our microarray data indicates a significant up-regulation of consortin.

mRNA in Muse-AT cells, which suggest that this protein may play a critical role in maintaining Muse-AT cell stemness and pluripotency, further indicating a need for future studies.

Recently, a potential source of error found in microarray analysis has been described [66] resulting from the comparison of heterogeneous cell populations that produced varied amounts of total RNA, which may result in masking of significant changes, as well as false positives. Recognizing this inherent potential for error in the microarray analysis, we attempted to partially address this issue by examining expression that was specific to either Muse-ATs or ASCs, with genes that were present in all ASCs and absent in all Muse-AT samples, and visa-versa. When considering fluctuations in total RNA, there typically exist fluctuations of several fold in total RNA [66], whereas in comparing mRNA that is present versus absent, fluctuation in sample expression is usually 2 to 3 orders of magnitude.

Many of the differentially expressed genes in Muse-AT cells are highly conserved, with homologues present in numerous small organisms (yeast, *S. Cerevisiae*, *C. elegans*, *chlamydomonas*, *T. californica*, *drosophila*, etc.). This indicates the possibility that Muse cells play a role in a highly conserved cellular mechanism related to cell survival in response to severe cellular stress [7], [67].

In normal adipose tissue, ASCs and Muse-ATs reside side by side and presumably interact during tissue differentiation, growth, and repair. Muse-ATs could not only play a critical role in repairing *in vivo* cellular damage by spontaneously differentiating into tissue-specific cells, but also may serve as regulatory cell, producing high levels of cell signaling molecules (e.g. chemokines and cytokines) to recruit and activate neighboring ASCs to differentiate and home to particular tissues. Future studies are needed to determine the interaction between Muse-ATs and ASCs during times of stress, injury, or inflammation.

In conclusion, we have isolated and characterized a novel population of pluripotent stem cells derived from adipose tissue without the utilization of FACS or magnetic microbead cell sorting. Muse-AT cells are highly resistant to severe cellular stress, are more likely to resist potential oncogenic effects, and therefore have the potential to make a critical impact on the field of regenerative medicine and cell-based therapy.

## Supporting Information

Table S1 **GO Analysis of the up-regulated genes in Muse-AT vs ASCs with 2 fold changes and p<0.05.**
(DOC)

Table S2 **GO Analysis of the down-regulated genes in Muse-AT vs ASCs with 2 fold changes and p<0.05.**
(DOC)

Table S3 **Genes expressed by Muse-AT that are not expressed by ASCs.**
(DOC)

Table S4 **Cluster of Differentiation (CD) genes expressed by Muse-AT that are not expressed by ASCs.**
(DOC)

Table S5 **Genes Expressed by ASCs that are not expressed in Muse-AT cells.**
(DOC)

Table S6 **Genes related to DNA stability.**
(DOC)
