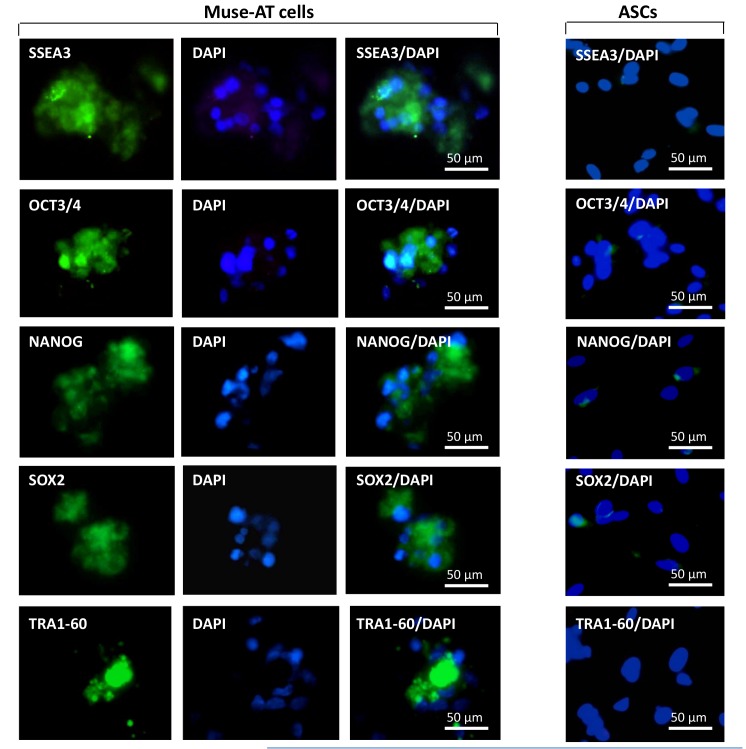# Correction: Awakened by Cellular Stress: Isolation and Characterization of a Novel Population of Pluripotent Stem Cells Derived from Human Adipose Tissue

**DOI:** 10.1371/annotation/190d4d01-a63c-4adc-a123-e519ee40a03e

**Published:** 2013-07-31

**Authors:** Saleh Heneidi, Ariel A. Simerman, Erica Keller, Prapti Singh, Xinmin Li, Daniel A. Dumesic, Gregorio Chazenbalk

There is an error in Figure 2. In the original figure, an image corresponding to the Nanog immunofluorescent staining was wrongly inserted into the mount for the Sox2 staining. The new version of Figure 2 has the correct photo of Sox2 staining inserted. Please see the correct version of Figure 2 at the following link: 

**Figure:**