# Supplementary material for: Awakened by Cellular Stress: Isolation and Characterization of a Novel Population of Pluripotent Stem Cells Derived from Human Adipose Tissue
Source: PLoS One. 2013 Jun 5;8(6):e64752. doi: 10.1371/journal.pone.0064752 (PMC3673968; doi:10.1371/journal.pone.0064752)
Supplement: Table S1 — GO Analysis of the up-regulated genes in Muse-AT vs ASCs with 2 fold changes and p<0.05. (DOC) [file pone.0064752.s001.doc]

**Supplemental Data: Table 1**

**GO Analysis of the up-regulated genes in Muse-AT vs ASCs with 2 fold changes and p < 0.05**

| No. | ProbeName | GeneSymbol | Gene Name | Fold change [aveMUSE] vs[aveASC] | Function |
| --- | --- | --- | --- | --- | --- |
| 1 | A_24_P257416 | CXCL2 | (C-X-C motif) ligand 2 | 777.8 | cell death and survival, organismal development, tissue development, organismal survival, cell to cell signaling and interaction, cellular growth and proliferation |
| 2 | A_19_P00317660 | LOC100505495 | LOC100505495 (LOC100505495) | 422.3 | und |
| 3 | A_33_P3245908 | C10orf128 | chromosome 10 open reading frame 128 | 248.5 | und |
| 4 | A_19_P00320718 | LOC100505495 | uncharacterized LOC100505495 | 210.8 | und |
| 5 | A_23_P72651 | ECSM2 | endothelial cell-specific chemotaxis regulator | 153.2 | und |
| 6 | A_23_P167920 | DLL1 | delta-like 1 (Drosophila) | 147.4 | cell death and survival, embryonic development, organismal development, tissue development, organismal survival, cellular development, growth and proliferation |
| 7 | A_23_P131208 | NR4A2 | nuclear receptor subfamily 4, group A, member 2 | 139.2 | cell death and survival, organismal development, cellular function and maintenance, DNA replication, recombination, and repair, cell cycle, organismal survival |
| 8 | A_32_P196263 | ADAMTS9 | ADAM metallopeptidase with thrombospondin type 1 motif, 9 | 115.3 | cancer, inflammatory diseases, protein sysnthesis, |
| 9 | A_23_P253602 | BMX | BMX non-receptor tyrosine kinase | 91.5 | cell death and survival, cellular growth and proliferation, cancer, hematological system development and function, cancer, post translational modification |
| 10 | A_33_P3423874 | MYZAP | myocardial zonula adherens protein | 87.6 | und |
| 11 | A_32_P100830 | KIF19 | kinesin family member 19 | 82.6 | und |
| 12 | A_23_P153320 | ICAM1 | intercellular adhesion molecule 1 | 50.2 | cell death and survival, embryonic development, organismal development, tisssue development, cellular function and maintenance, organ development, organismal survival |
| 13 | A_24_P942630 | JMJD3 | lysine (K)-specific demethylase 6B | 47.4 | und |
| 14 | A_24_P73577 | ALDH1A2 | aldehyde dehydrogenase 1 family, member A2 | 47.1 | cell death and survival, embryonic development, organismal development, tissue development, nervous system development and function, tissue morphology |
| 15 | A_23_P134176 | SOD2 | superoxide dismutase 2 | 41.4 | cell death and survival, embryonic developmental, organismal development, tissue development, organ development, organismal survival |
| 16 | A_23_P132121 | SNF1LK | salt-inducible kinase 1 | 33.7 | und |
| 17 | A_32_P189592 | USHBP1 | Usher syndrome 1C binding protein 1 | 32.8 | und |
| 18 | A_24_P380679 | C7orf53 | chromosome 7 open reading frame 53 | 30.8 | und |
| 19 | A_23_P137470 | SIPA1L2 | signal-induced proliferation-associated 1 like 2 | 30.0 | und |
| 20 | A_23_P319423 | KCNK5 | potassium channel, subfamily K, member 5 | 28.1 | hematological system development and function |
| 21 | A_33_P3550894 | GATA2 | GATA binding protein 2 | 27.6 | cell death and survival, embryonic development, gene expression, cell cycle, tissue morphology, cellular development, growth, and proliferation |
| 22 | A_33_P3254331 | CTRC | chymotrypsin C (caldecrin) | 27.2 | und |
| 23 | A_33_P3370575 | CD200 | CD200 molecule | 26.0 | cell death and survival, organismal survival, infectious disease, cell morphology, molecular transport, neurological disease |
| 24 | A_23_P10025 | NELL2 | NEL-like 2 (chicken) | 24.6 | und |
| 25 | A_23_P302207 | DKFZp434J1015 | zinc finger protein 853 | 22.0 | und |
| 26 | A_23_P112289 | TMOD1 | tropomodulin 1 (TMOD1) | 20.8 | embryonic development, organismal development, tissue development, cellular assembly and organization, cellular function and maintenance, organismal survival |
| 27 | A_23_P139704 | DUSP6 | dual specificity phosphatase 6 | 20.0 | cell death and survival, cellular growth and proliferation, post translational modification |
| 28 | A_23_P41765 | IRF1 | interferon regulatory factor 1 | 19.9 | cell death and survival, organismal development, cellular assembly and organization, cellular function and maintenance, cell cycle, organismal survival |
| 29 | A_23_P59877 | FABP5 | fatty acid binding protein 5 | 18.8 | organismal development, small molecule biochemistry, cellular growth and proliferation, lipid metabolism, molecular transport, cancer |
| 30 | A_33_P3245415 | N4BP2L1 | NEDD4 binding protein 2-like 1 | 17.9 | und |
| 31 | A_33_P3214720 | ZC3H12A | zinc finger CCCH-type containing 12A | 16.9 | cell death and survival, organismal development, organismal survival |
| 32 | A_32_P218025 | LOC100506253 | hypothetical LOC100506253 | 16.5 | und |
| 33 | A_19_P00316324 | LOC100506810 | uncharacterized LOC100506810 | 16.1 | und |
| 34 | A_23_P66260 | ZNF267 | zinc finger protein 267 (ZNF267), transcript variant 498723 | 15.3 | cellular growth and proliferation |
| 35 | A_23_P118042 | LRRC36 | leucine rich repeat containing 36 (LRRC36), transcript variant 1 | 15.2 | und |
| 36 | A_33_P3370812 | TBC1D1 | TBC1 (tre-2/USP6, BUB2, cdc16) | 14.9 | und |
| 37 | A_23_P153155 | GALR1 | galanin receptor 1 | 14.4 | und |
| 38 | A_23_P79108 | ATP8B3 | ATPase, aminophospholipid transporter, class I | 14.3 | energy production, nucleic acid metabolism, DNA replication, recombination, and repair, lipd metabolism, molecular transport |
| 39 | A_23_P63371 | TAL1 | T-cell acute lymphocytic leukemia 1 | 14.0 | cell death and survival, embryonic development, organismal development, tissue development, cell cycle, organismal survival |
| 40 | A_33_P3268507 | CEACAM1 | carcinoembryonic antigen-related cell adhesion molecule 1 | 13.4 | cell death and survival, embryonic developmental, organismal development, tissue development, cell cycle, infectious disease, cellular development, growth and proliferation |
| 41 | A_24_P145911 | SFRS10 | transformer 2 beta homolog | 13.4 | und |
| 42 | A_23_P92042 | ITPR1 | inositol 1,4,5-trisphosphate receptor, type 1 | 12.5 | cell death and survival, organismal development, nervous system development and function, organ development, organismal survival, cell morphology |
| 43 | A_33_P3424566 | FLJ38717 | FLJ38717 protein (FLJ38717), | 12.0 | und |
| 44 | A_33_P3462422 | C6orf190 | thymocyte selection associated (THEMIS), transcript variant 1 | 11.8 | und |
| 45 | A_19_P00318096 |  | lincRNA:chr8:90736857-90737871 reverse strand | 11.4 | und |
| 46 | A_23_P34452 | LOR | loricrin | 11.4 | organismal development, dermatological disease and conditions, organismal injury and abnormalities, hereditary disorders |
| 47 | A_23_P48029 | CLEC4A | C-type lectin domain family 4, member A | 11.4 | und |
| 48 | A_32_P98072 | TCHH | trichohyalin | 11.1 | und |
| 49 | A_33_P3245480 | LOC100130000 | phosphodiesterase 4D interacting protein pseudogene | 10.8 | und |
| 50 | A_23_P4662 | BCL3 | B-cell CLL/lymphoma 3 (BCL3), mRNA [NM_005178] | 10.7 | cell death and survival, organismal development, DNA replication, recombination, and repair, cell cycle, organismal survival, cellular growth and proliferation |
| 51 | A_23_P38206 | LSMD1 | LSM domain containing 1 | 10.7 | cell death and survival |
| 52 | A_33_P3288942 | FAM107B | family with sequence similarity 107, member B | 10.6 | cell death and survival |
| 53 | A_23_P210330 | HSPC159 | Homo sapiens lectin, galactoside-binding-like (LGALSL) | 10.5 | und |
| 54 | A_33_P3337267 | NRARP | NOTCH-regulated ankyrin repeat protein | 10.5 | embryonic developmental, organismal development, tissue development, cell function and maintenance, cellular growth and proliferation |
| 55 | A_23_P140928 | TMC7 | transmembrane channel-like 7 transcript variant 1 | 10.3 | und |
| 56 | A_33_P3297517 | SFRS10 | transformer 2 beta homolog (Drosophila) | 10.1 | und |
| 57 | A_19_P00318561 | LOC100506516 | hypothetical LOC100506516 | 9.8 | und |
| 58 | A_24_P377775 | RGS3 | regulator of G-protein signaling 3 | 9.7 | cell death and survival, tissue development, cell to cell signaling and interaction |
| 59 | A_33_P3211443 | LRRC8B | leucine rich repeat containing 8 family, member B | 9.6 | und |
| 60 | A_33_P3301351 | RBM44 | RNA binding motif protein 44 | 9.6 | und |
| 61 | A_33_P3719083 | CHRFAM7A | cholinergic receptor, nicotinic, alpha 7, exons 5-10),family with sequence similarity 7A, exons A-E) fusion transcript variant 1 | 9.5 | und |
| 62 | A_32_P177539 | IRX6 | iroquois homeobox 6 (IRX6) | 9.5 | cellular growth and proliferation |
| 63 | A_33_P3214035 | ADORA2A | adenosine A2a receptor | 9.3 | cell death and survival, embryonic development, organismal development, tissue development, cellular function and maintenance, organ development, organismal survival |
| 64 | A_23_P30655 | NFKBIE | nuclear factor of kappa light polypeptide gene enhancer in B-cells inhibitor, epsilon | 9.2 | Reproductive System Development and Function |
| 65 | A_23_P103110 | MAFF | v-maf musculoaponeurotic fibrosarcoma oncogene homolog F (avian) | 9.1 | organismal development, gene expression, organismal survival, cellular growth and proliferation, RNA post-transcriptional modifications |
| 66 | A_23_P163195 | LRFN5 | leucine rich repeat and fibronectin type III domain containing 5 | 9.1 | Protein trafficking |
| 67 | A_23_P203391 | ASRGL1 | asparaginase like 1 (ASRGL1), transcript variant 1 | 9.1 | und |
| 68 | A_33_P3279831 | PNRC1 | proline-rich nuclear receptor coactivator 1 | 9.0 | infectious disease, hematopoiesis |
| 69 | A_33_P3393766 | C17orf96 | chromosome 17 open reading frame 96 | 8.9 | und |
| 70 | A_32_P23308 | LOC257358 | uncharacterized LOC257358 | 8.7 | und |
| 71 | A_23_P116264 | NRGN | neurogranin (protein kinase C substrate, RC3), transcript variant 1 | 8.4 | cell death and survival, nervous system development, cell to cell signaling and interaction |
| 72 | A_33_P3329098 | WTAP | Wilms tumor 1 associated protein, transcript variant 1 | 8.2 | cell death and survival, cellular growth and proliferation |
| 73 | A_32_P122226 | AMDHD1 | amidohydrolase domain containing 1 | 8.2 | und |
| 74 | A_19_P00320293 |  | lincRNA:chr22:27066108-27066654 reverse strand | 8.1 | und |
| 75 | A_19_P00324774 |  | lincRNA:chr3:13093175-13147525 reverse strand | 8.0 | und |
| 76 | A_23_P326474 | L3MBTL4 | l(3)mbt-like 4 (Drosophila) | 7.9 | und |
| 77 | A_33_P3710442 | FLJ11710 | cDNA FLJ11710 fis, clone HEMBA1005149 | 7.9 | und |
| 78 | A_33_P3375934 | NAMPT | nicotinamide phosphoribosyltransferase | 7.8 | cell death and survival, cell cycle, cellular development, cellular growth and proliferation |
| 79 | A_23_P150343 | SLN | sarcolipin | 7.8 | und |
| 80 | A_23_P62642 | CCDC19 | coiled-coil domain containing 19 | 7.7 | cellular growth and proliferation |
| 81 | A_23_P104318 | DDIT4 | DNA-damage-inducible transcript 4 | 7.7 | cell death and survival, cellular function and maintenance, cell morphology, tumor morphology |
| 82 | A_33_P3423949 | CBX2 | chromobox homolog 2 (CBX2) | 7.6 | embryonic developmental, organismal development, tissue development, cell cycle, organismal survival, cellular development, growth and proliferation |
| 83 | A_33_P3420266 | ANKRD20A2 | ankyrin repeat domain 20 family, member A2 | 7.6 | und |
| 84 | A_33_P3332156 | C1orf95 | chromosome 1 open reading frame 95 | 7.5 | und |
| 85 | A_33_P3412418 |  | cDNA FLJ40480 fis, clone TESTI2043313 | 7.5 | und |
| 86 | A_23_P93269 | ZNF165 | zinc finger protein 165 | 7.2 | und |
| 87 | A_33_P3334102 | ARHGAP27 | Rho GTPase activating protein 27 | 7.2 | und |
| 88 | A_19_P00806923 |  | lincRNA:chr7:66004115-66042779 reverse strand | 7.1 | und |
| 89 | A_33_P3659678 | NR6A1 | nuclear receptor subfamily 6, group A, member 1 | 7.1 | embryonic development, organismal development, tissue development, gene expression, organsimal survival, cellular growth and proliferation |
| 90 | A_19_P00318213 | HCG18 | HLA complex group 18 (non-protein coding) | 7.0 | und |
| 91 | A_33_P3234025 |  | chromosome 1 open reading frame 196 | 7.0 | und |
| 92 | A_23_P125809 | ZCCHC12 | zinc finger, CCHC domain containing 12 | 6.7 | und |
| 93 | A_23_P134953 | ADFP | perilipin 2 (PLIN2) | 6.7 | lipid metabolism |
| 94 | A_33_P3211513 | CLK1 | CDC-like kinase 1 (CLK1), transcript variant 2 | 6.5 | organismal development, cell cycle, organismal survival, infectious disease, cell development, growth and proliferation, connective tissue development and function |
| 95 | A_19_P00323211 |  | lincRNA:chr1:32515613-32529913 reverse strand | 6.5 | und |
| 96 | A_19_P00813171 |  | lincRNA:chr2:19532269-19547044 reverse strand | 6.5 | und |
| 97 | A_33_P3367795 | RPS6 | ribosomal protein S6 | 6.4 | cell cycle, cell death and survival, hematological disease, infectious disease, cell development, growth and proliferation, connective tissue development and function |
| 98 | A_19_P00328463 |  | lincRNA:chr6:86381806-86396056 reverse strand | 6.3 | und |
| 99 | A_23_P94546 | GKAP1 | G kinase anchoring protein 1 , transcript variant 1 | 6.2 | cell death and survival |
| 100 | A_33_P3341906 | C10orf128 | chromosome 10 open reading frame 128 | 6.1 | cell death and survival |
| 101 | A_33_P3413840 | GK | glycerol kinase (GK), transcript variant 4 | 6.1 | tissue development, cellular assembly and organization, cellular function and maintenance, DNA replication and repair, cell cycle, metabolic disorders |
| 102 | A_33_P3299656 | RPGR | retinitis pigmentosa GTPase regulator (RPGR) | 6.0 | und |
| 103 | A_23_P342053 | RBBP6 | retinoblastoma binding protein 6 | 6.0 | cell death and survival, embryonic development, organismal development, organismal survival, molecular transport, hereditary disorder |
| 104 | A_23_P100386 | IL34 | interleukin 34 (IL34), transcript variant 1 | 5.8 | cellular growth and proliferation, post translational modifications |
| 105 | A_19_P00320359 |  | lincRNA:chr7:130600777-130606704 reverse strand | 5.8 | und |
| 106 | A_33_P3346688 | HSPA8 | heat shock 70kDa protein 8 | 5.8 | cell death and survival, DNA replication, recombination, and repair, energy production, nuclear acid metabolism, cell cycle, organismal injuries and abnormalities |
| 107 | A_23_P9485 | ORM2 | orosomucoid 2 | 5.6 | und |
| 108 | A_23_P212595 | VPRBP | Vpr (HIV-1) binding protein (VPRBP), transcript variant 1 | 5.6 | cell death and survival, embryonic development, organismal development, cell cycle, organismal survival, growth and proliferation |
| 109 | A_33_P3377346 | LOC100132526 | FYVE, RhoGEF and PH domain containing 5 pseudogene | 5.6 | und |
| 110 | A_23_P114057 | SEMA4C | sema domain, immunoglobulin domain (Ig), transmembrane domain (TM) and short cytoplasmic domain | 5.5 | cellular growth and proliferation |
| 111 | A_23_P135576 | PTPRT | protein tyrosine phosphatase, receptor type, T, transcript variant 1 | 5.5 | und |
| 112 | A_23_P89621 | CBX4 | chromobox homolog 4 | 5.4 | und |
| 113 | A_32_P8813 | LOC283663 | uncharacterized LOC283663 | 5.4 | und |
| 114 | A_23_P70201 | CHD1 | chromodomain helicase DNA binding protein 1 | 5.4 | gene expression, infection diseases |
| 115 | A_23_P69109 | PLSCR1 | phospholipid scramblase 1 | 5.4 | cell death and survival, cellular assembly and organization, gene expression, cellular development, growth and proliferation, lipid metabolism, molecular transport |
| 116 | A_24_P130936 | DDX3Y | DEAD (Asp-Glu-Ala-Asp) box polypeptide 3, Y-linked, transcript variant 2 | 5.3 | und |
| 117 | A_23_P83781 | PSCD1 | cytohesin 1 (CYTH1), transcript variant 1 | 5.3 | und |
| 118 | A_33_P3332474 | LOC283767 | golgin A6 family-like 1 | 5.3 | und |
| 119 | A_24_P818010 | LOC100133161 | uncharacterized LOC100133161, non-coding RNA | 5.2 | und |
| 120 | A_33_P3329750 |  |  | 5.2 | und |
| 121 | A_32_P64475 | C1orf55 | chromosome 1 open reading frame 55 | 5.2 | und |
| 122 | A_19_P00804882 |  | lincRNA:chr12:9277383-9292308 forward strand | 5.1 | und |
| 123 | A_23_P211985 | SNRK | SNF related kinase (SNRK), transcript variant 1 | 5.1 | cell death and survival, post-Translational Modification |
| 124 | A_19_P00327303 |  | lincRNA:chr10:74758344-74765569 reverse strand | 5.1 | und |
| 125 | A_33_P3336252 | OR7E37P | olfactory receptor, family 7, subfamily E, member 37 pseudogene | 5.0 | und |
| 126 | A_32_P219520 | TNFAIP8 | tumor necrosis factor, alpha-induced protein 8 (TNFAIP8), transcript variant 1 | 5.0 | cell death and survival, cellular gowth and proliferation |
| 127 | A_23_P16817 | CLK1 | CDC-like kinase 1 (CLK1), transcript variant 1 | 4.9 | organismal development, cell cycle, organismal survival, cellular growth and proliferation, post-translational modification, RNA post-transcriptional modification |
| 128 | A_19_P00801521 |  | lincRNA:chr6:86381806-86396056 reverse strand | 4.9 | und |
| 129 | A_23_P203191 | APOA1 | apolipoprotein A-I | 4.9 | cell death and survival, tissue development, nucleic acid metabolism, organismal injury and abnormalities, cell to cell signaling and interaction, cellular growth and proliferation |
| 130 | A_32_P72110 | PVR | poliovirus receptor , transcript variant 1 | 4.9 | cell death and survival, cellular assembly and organization, infectious disease, cell to cell signaling and abnormalities, cancer, neurological disease |
| 131 | A_24_P257336 | POPDC2 | popeye domain containing 2 | 4.9 | und |
| 132 | A_23_P136964 | RPGR | retinitis pigmentosa GTPase regulator (RPGR), transcript variant A | 4.8 | molecular transport, hereditary disorder, protein trafficking, ophthalmological disease, hereditary disorder, protein trafficking |
| 133 | A_19_P00806590 |  | lincRNA:chr5:36701011-36702056 reverse strand | 4.8 | und |
| 134 | A_33_P3881056 |  | cDNA FLJ27128 fis, clone SPL07659 | 4.8 | und |
| 135 | A_33_P3336720 | HAMP | hepcidin antimicrobial peptide | 4.7 | organismal development, hematological disease, organismal survival |
| 136 | A_33_P3250323 |  |  | 4.7 | und |
| 137 | A_32_P117464 | C3orf59 | Mab-21 domain containing 2 | 4.6 | und |
| 138 | A_23_P3956 | C1QTNF1 | C1q and tumor necrosis factor related protein 1 | 4.6 | Protein Synthesis |
| 139 | A_33_P3424328 | FLJ43093 | RAB44, member RAS oncogene family | 4.5 | und |
| 140 | A_33_P3398411 | RASSF3 | Ras association (RalGDS/AF-6) domain family member 3, transcript variant 1 | 4.5 | und |
| 141 | A_23_P209805 | NAB1 | NGFI-A binding protein 1 (EGR1 binding protein 1) | 4.5 | organismal development |
| 142 | A_33_P3337415 | GNAL | guanine nucleotide binding protein (G protein), alpha activating activity polypeptide, olfactory type, transcript variant 2 | 4.5 | organismal development, organismal survival |
| 143 | A_32_P524904 | FLJ22675 | chromosome 11 open reading frame 86 (C11orf86) | 4.5 | und |
| 144 | A_23_P1682 | TMEM45B | transmembrane protein 45B | 4.5 | und |
| 145 | A_19_P00330413 |  | lincRNA:chr13:106751024-106760449 reverse strand | 4.4 | und |
| 146 | A_19_P00319561 | JPX | JPX transcript, XIST activator (non-protein coding) (JPX), non-coding RNA | 4.4 | und |
| 147 | A_33_P3407895 | FLJ45909 | Ras and Rab interactor-like (RINL), transcript variant 1 | 4.4 | und |
| 148 | A_24_P135769 | LOH11CR2A | von Willebrand factor A domain containing 5A, transcript variant 2 | 4.4 | embryonic development, cellular assembly and organization, cellular growth and proliferation, post translational modification |
| 149 | A_32_P61684 | PAG1 | phosphoprotein associated with glycosphingolipid microdomains 1 | 4.3 | cellular assembly and organization |
| 150 | A_23_P397347 | MCM9 | minichromosome maintenance complex component 9, transcript variant 2 | 4.3 | und |
| 151 | A_24_P137997 | ZNF34 | zinc finger protein 34 | 4.3 | und |
| 152 | A_23_P106874 | PMFBP1 | polyamine modulated factor 1 binding protein 1, transcript variant 1 | 4.3 | und |
| 153 | A_33_P3711933 | FLJ32810 | Rho GTPase activating protein 42 | 4.3 | und |
| 154 | A_19_P00803333 | ZSWIM6 | zinc finger, SWIM-type containing 6 | 4.3 | und |
| 155 | A_33_P3259183 | FAM78B | family with sequence similarity 78, member B | 4.3 | und |
| 156 | A_33_P3319140 |  | cDNA FLJ46080 fis, clone TESTI2004971 | 4.2 | und |
| 157 | A_19_P00807053 | LOC100506411 | PREDICTED: Homo sapiens hypothetical LOC100506411 | 4.2 | und |
| 158 | A_33_P3300495 |  | plasminogen-like B1 | 4.1 | und |
| 159 | A_33_P3620087 | LOC400756 | hypothetical gene supported by BC030752 | 4.1 | und |
| 160 | A_33_P3332348 | RN7SL1 | 7SL, cytoplasmic 1, small cytoplasmic RNA | 4.1 | und |
| 161 | A_33_P3293169 | AMBRA1 | autophagy/beclin-1 regulator 1 | 4.1 | cell death and survival, cellular growth and proliferation |
| 162 | A_23_P4294 | ZNF232 | zinc finger protein 232 | 4.1 | und |
| 163 | A_24_P9671 | DNAJA1 | DnaJ (Hsp40) homolog, subfamily A, member 1 | 4.1 | cell death and survival, embryonic development, organismal development, DNA replication, recombination, and repair, molecular transport |
| 164 | A_33_P3293675 | SLC12A7 | solute carrier family 12 (potassium/chloride transporters), member 7 | 4.0 | cell death and survival, organismal development, hematological diseases |
| 165 | A_33_P3251430 | NRIP1 | nuclear receptor interacting protein 1 | 4.0 | embryonic development, gene expression, cell cycle, cellular growth and proliferation, molecular transport, carbohydrate metabolism, connective tissue development |
| 166 | A_32_P167239 | AFAP1L1 | actin filament associated protein 1-like 1, transcript variant 1 | 4.0 | cellular growth and proliferation, cellular movement, cancer |
| 167 | A_19_P00326172 |  | lincRNA:chr15:75201097-75208047 reverse strand | 4.0 | und |
| 168 | A_24_P9321 | HIST1H3I | histone cluster 1, H3i | 4.0 | und |
| 169 | A_33_P3248787 |  | DB221055 TRACH3 Homo sapiens cDNA clone TRACH3011617 5' | 4.0 | und |
| 170 | A_24_P590560 | RRN3 | RRN3 RNA polymerase I transcription factor homolog (S. cerevisiae) | 4.0 | cell death and survival, embryonic development, tissue development, cellular assembly and organization, cellular function and maintenance, DNA replication, recombination, and repair |
| 171 | A_19_P00813468 |  | lincRNA:chr18:72258320-72282445 forward strand | 4.0 | und |
| 172 | A_19_P00332333 |  | lincRNA:chr16:72314424-72332999 forward strand | 4.0 | und |
| 173 | A_33_P3445679 | FLJ42392 | uncharacterized LOC400123 | 3.9 | und |
| 174 | A_23_P144123 | SLC22A13 | solute carrier family 22 (organic anion transporter), member 13 | 3.9 | und |
| 175 | A_23_P166 | MOBKL2C | MOB kinase activator 3C, transcript variant 1 | 3.9 | und |
| 176 | A_24_P30923 | SNN | stannin | 3.9 | cell death and survival, infectious disease |
| 177 | A_24_P411863 | MLL5 | myeloid/lymphoid or mixed-lineage leukemia 5 (trithorax homolog, Drosophila), transcript variant 1 | 3.9 | embryonic development, organismal development, cell cycle, organ morphology, organismal survival, lymphoid tissue structure and development |
| 178 | A_23_P99044 | KRT71 | keratin 71 | 3.9 | und |
| 179 | A_33_P3333033 | SGSM2 | cDNA FLJ42893 fis, clone BRHIP3008598 | 3.8 | und |
| 180 | A_33_P3275943 | PWWP2A | PWWP domain containing 2A, transcript variant 1 | 3.8 | und |
| 181 | A_24_P277615 | TRIM7 | tripartite motif containing 7, transcript variant 2 | 3.8 | infectious disease |
| 182 | A_33_P3256695 | PRPF40A | PRP40 pre-mRNA processing factor 40 homolog A (S. cerevisiae) | 3.8 | und |
| 183 | A_24_P144601 | POU5F1 | POU class 5 homeobox 1, transcript variant 1 | 3.8 | cell death and survival, gene expression, cell cycle, cell to cell signaling and interaction, cell growth and proliferation, development disorder |
| 184 | A_23_P164258 | PIPOX | pipecolic acid oxidase | 3.8 | und |
| 185 | A_32_P213637 | FLJ14186 | uncharacterized LOC401149 | 3.8 | und |
| 186 | A_23_P92642 | ANKHD1 | ankyrin repeat and KH domain containing 1, transcript variant 3 | 3.7 | und |
| 187 | A_23_P335239 | GAB1 | GRB2-associated binding protein 1 , transcript variant 1 | 3.7 | cell death and survival, embryonic development, organismal development, tissue development, cell cycle, organ development, organismal survival |
| 188 | A_33_P3265679 | LOC389199 | hypothetical LOC389199 | 3.7 | und |
| 189 | A_23_P401055 | SOX2 | SRY (sex determining region Y)-box 2 (SOX2), mRNA [NM_003106] | 3.7 | embryonic development, organismal survival, tissue development, nervous system development, cell cycle, organ development |
| 190 | A_33_P3353330 |  | cDNA FLJ40828 fis, clone TRACH2011574 | 3.7 | und |
| 191 | A_24_P943997 | ARL5B | ADP-ribosylation factor-like 5B | 3.7 | und |
| 192 | A_32_P125338 | FAM43B | family with sequence similarity 43, member B | 3.7 | und |
| 193 | A_33_P3288364 | SPATA2L | spermatogenesis associated 2-like | 3.7 | und |
| 194 | A_23_P108082 | CREB3L3 | cAMP responsive element binding protein 3-like 3 | 3.7 | cellular growth and proliferation, inflammatory response |
| 195 | A_23_P53015 | TUT1 | uridylyl transferase 1, U6 snRNA-specific | 3.7 | und |
| 196 | A_23_P434347 | ITSN2 | intersectin 2, transcript variant 2 | 3.7 | infectious disease |
| 197 | A_33_P3241596 |  |  | 3.7 | und |
| 198 | A_33_P3230011 | STOX1 | storkhead box 1, transcript variant 3 | 3.6 | cardiovascular disease |
| 199 | A_23_P19673 | SGK1 | serum/glucocorticoid regulated kinase 1, transcript variant 1 | 3.6 | cell death and survival, tissue development, cellular assembly and organization, cellular function and maintenance, DNA replication, recombination and repair, cell cycle |
| 200 | A_19_P00328932 |  | lincRNA:chr2:129195780-129215280 reverse strand | 3.6 | und |
| 201 | A_24_P458479 |  | Putative protein FAM27E1 | 3.6 | und |
| 202 | A_19_P00805718 |  | lincRNA:chr13:33916689-33917170 reverse strand | 3.6 | und |
| 203 | A_23_P97584 | DNTTIP2 | deoxynucleotidyltransferase, terminal, interacting protein 2 | 3.6 | und |
| 204 | A_23_P213592 | RNF44 | ring finger protein 44 | 3.5 | und |
| 205 | A_33_P3313779 | CCDC64 | coiled-coil domain containing 64 | 3.5 | und |
| 206 | A_19_P00318165 |  | lincRNA:chr6:86382986-86383808 reverse strand | 3.5 | und |
| 207 | A_23_P162766 | DOCK9 | dedicator of cytokinesis 9, transcript variant 1 | 3.4 | und |
| 208 | A_33_P3313929 | CCR6 | chemokine (C-C motif) receptor 6, transcript variant 2 | 3.4 | embryonic development, organismal development, tissue development, organ development, infectious disease, cellular development, growth and proliferation |
| 209 | A_23_P126266 | HLX | H2.0-like homeobox | 3.4 | tissue development, hematological disease, infectious disease, cellular growth and proliferation |
| 210 | A_33_P3327250 |  |  | 3.4 | und |
| 211 | A_33_P3302696 | PPM1D | protein phosphatase, Mg2+/Mn2+ dependent, 1D | 3.4 | cell death and survival, organismal development, cell cycle, organ morphology, organismal survival, cellular growth and proliferation, cancer |
| 212 | A_23_P2801 | ELF1 | E74-like factor 1 (ets domain transcription factor), transcript variant 1 | 3.4 | gene expression, cellular growth and proliferation, post translational modification, |
| 213 | A_23_P321223 | PMCH | pro-melanin-concentrating hormone | 3.4 | organismal development, Nucleic acid metabolism, small molecule biochemistry, molecular transport, carbohydrate metabolism |
| 214 | A_23_P93360 | AGER | advanced glycosylation end product-specific receptor, transcript variant 1 | 3.4 | cell death and survival, organismal development, tissue development, cellular assembly and organization, organ development and morphology, organismal survival |
| 215 | A_23_P140450 | SLC27A2 | solute carrier family 27 (fatty acid transporter), member 2, transcript variant 1 | 3.4 | small molecule biochemistry, lipid metabolism, molecular transport |
| 216 | A_24_P196704 | MLL5 | myeloid/lymphoid or mixed-lineage leukemia 5 (trithorax homolog, Drosophila), transcript variant 1 | 3.3 | organismal development, tissue development, cell cycle, organ morphology, organismal survival, lymphoid tissue structure and development, cell signaling |
| 217 | A_23_P94762 | ZNF354B | zinc finger protein 354B | 3.3 | und |
| 218 | A_32_P231493 |  | clone IMAGE:1257951 | 3.3 | und |
| 219 | A_23_P341223 | KLHL21 | kelch-like 21 (Drosophila) | 3.3 | cell cycle, cellular movement |
| 220 | A_23_P97677 | MATN1 | matrilin 1, cartilage matrix protein | 3.3 | und |
| 221 | A_23_P160025 | IFI16 | interferon, gamma-inducible protein 16, transcript variant 2 | 3.3 | cell death and survival, DNA replication, recombination, and repair, cell cycle, cellular development, organ morphology, cellular growth and proliferation |
| 222 | A_23_P13359 | NXF1 | nuclear RNA export factor 1 (NXF1), transcript variant 1 | 3.2 | und |
| 223 | A_33_P3371493 | TOP1 | topoisomerase (DNA) I | 3.2 | cell death and survival, organismal development, cellular assembly and organization, cellular function and maintenance, DNA replication, recombination, and repair, cell cycle, organismal survival |
| 224 | A_24_P751074 | ETS1 | v-ets erythroblastosis virus E26 oncogene homolog 1 (avian), transcript variant 2 | 3.2 | cell death and survival, tissue development, cellular assembly and organization, cellular function and maintenance, cell cycle, tissue and morphology, organismal survival |
| 225 | A_33_P3255499 | MCART1 | mitochondrial carrier triple repeat 1, transcript variant 2 | 3.1 | und |
| 226 | A_23_P107051 | TCAP | titin-cap (telethonin) | 3.1 | und |
| 227 | A_24_P85317 | CHD2 | chromodomain helicase DNA binding protein 2, transcript variant 2 | 3.1 | gene expression |
| 228 | A_33_P3259821 | DOCK9 | dedicator of cytokinesis 9, transcript variant 4 | 3.1 | und |
| 229 | A_33_P3290085 | ZNF394 | zinc finger protein 394 | 3.1 | und |
| 230 | A_33_P3271990 | DGAT1 | diacylglycerol O-acyltransferase 1 | 3.1 | organismal survival, organ morphology, organismal injury and abnormalities, infectious disease, lipid metabolism, molecular transport |
| 231 | A_33_P3237517 | ZNF292 | zinc finger protein 292 | 3.1 | und |
| 232 | A_19_P00324768 |  | lincRNA:chr15:70543146-70563646 forward strand | 3.1 | und |
| 233 | A_23_P202104 | PPIF | peptidylprolyl isomerase F (PPIF), nuclear gene encoding mitochondrial protein | 3.1 | cell death and survival, organismal development, cellular assembly and organization, cellular function and maintenance, organismal survival |
| 234 | A_23_P61050 | MLKL | mixed lineage kinase domain-like, transcript variant 1 | 3.1 | cell death and survival |
| 235 | A_33_P3302055 | LOC728073 | cDNA DKFZp762K239 (from clone DKFZp762K239) | 3.1 | und |
| 236 | A_33_P3334419 | RPS6KL1 | ribosomal protein S6 kinase-like 1 | 3.0 | cell death and survival |
| 237 | A_33_P3526315 | MGC12488 | clone IMAGE:3932794 | 3.0 | und |
| 238 | A_23_P312718 | PNPLA8 | patatin-like phospholipase domain containing 8 | 3.0 | organismal development, cellular assembly and organization, cellular function and maintenance, small molecule biochemistry, organismal survival, lipid metabolism |
| 239 | A_33_P3405946 | RBM39 | RNA binding motif protein 39, transcript variant 1 | 3.0 | gene expression |
| 240 | A_23_P58407 | UGT2B15 | UDP glucuronosyltransferase 2 family, polypeptide B15 | 3.0 | cellular growth and proliferation |
| 241 | A_23_P109254 | RBM39 | RNA binding motif protein 39, transcript variant 1 | 3.0 | gene expression |
| 242 | A_33_P3363645 | MGAT1 | mannosyl (alpha-1,3-)-glycoprotein beta-1,2-N-acetylglucosaminyltransferase, transcript variant 1 | 3.0 | embryonic development, organismal development, tissue development, infectious disease, cellular development, growth and proliferation, |
| 243 | A_24_P391104 | RFX1 | regulatory factor X, 1 (influences HLA class II expression) | 3.0 | cellular growth and proliferation |
| 244 | A_23_P154526 | GRB14 | growth factor receptor-bound protein 14 | 3.0 | und |
| 245 | A_23_P82979 | LAMC3 | laminin, gamma 3 | 3.0 | infectious disease, cellular growth and proliferation |
| 246 | A_23_P258002 | CDKN2AIP | CDKN2A interacting protein | 2.9 | und |
| 247 | A_23_P170608 | TSPYL2 | TSPY-like 2 | 2.9 | tissue morphology, cellular growth and proliferation |
| 248 | A_33_P3315801 | CRKRS | cyclin-dependent kinase 12, transcript variant 1 | 2.9 | und |
| 249 | A_33_P3284584 |  |  | 2.9 | und |
| 250 | A_24_P145316 | DTNBP1 | dystrobrevin binding protein 1, transcript variant 2 | 2.9 | metabolic disorder, dermatological disease and conditions, organismal injuries and abnormalities, development disorder, hereditary disorder, ophthalmological disease |
| 251 | A_33_P3220853 |  |  | 2.9 | und |
| 252 | A_33_P3227041 | BID | BH3 interacting domain death agonist, transcript variant 1 | 2.9 | cell death and survival, organismal development, cellular assembly and organization, cellular function and maintenance, cell cycle, organismal survival |
| 253 | A_19_P00809147 |  | lincRNA:chr8:52809004-52810047 reverse strand | 2.9 | und |
| 254 | A_23_P156993 | CNOT4 | CCR4-NOT transcription complex, subunit 4 , transcript variant 2 | 2.9 | und |
| 255 | A_24_P177553 |  | cDNA clone IMAGE:6744194 5' | 2.9 | und |
| 256 | A_23_P20275 | PLEKHF2 | pleckstrin homology domain containing, family F (with FYVE domain) member 2 | 2.9 | und |
| 257 | A_24_P916378 | HNRPLL | heterogeneous nuclear ribonucleoprotein L-like | 2.9 | und |
| 258 | A_24_P371399 | C3orf58 | chromosome 3 open reading frame 58 , transcript variant 1 | 2.9 | und |
| 259 | A_23_P211007 | NRIP1 | nuclear receptor interacting protein 1 | 2.8 | embryonic development, cell cycle |
| 260 | A_23_P143845 | TIPARP | TCDD-inducible poly(ADP-ribose) polymerase, transcript variant 2 | 2.8 | und |
| 261 | A_24_P576174 | DCP1A | DCP1 decapping enzyme homolog A (S. cerevisiae) | 2.8 | gene expression |
| 262 | A_33_P3385782 | ZNF713 | zinc finger protein 713 | 2.8 | und |
| 263 | A_33_P3238525 |  | clone IMAGE:4424208 | 2.8 | und |
| 264 | A_32_P191004 | ATAD2B | ATPase family, AAA domain containing 2B, transcript variant 1 | 2.8 | und |
| 265 | A_24_P126060 | DDX3X | DEAD (Asp-Glu-Ala-Asp) box polypeptide 3, X-linked, transcript variant 1 | 2.8 | cell death and survival, infectious disease, cellular development, cellular growth and proliferation, connective tissue development |
| 266 | A_33_P3235706 | ZCCHC11 | zinc finger, CCHC domain containing 11, mRNA (cDNA clone IMAGE:5505348), with apparent retained intron | 2.8 | und |
| 267 | A_23_P401361 | PITPNM2 | phosphatidylinositol transfer protein, membrane-associated 2 | 2.8 | und |
| 268 | A_33_P3322430 | CTNS | cystinosin, lysosomal cystine transporter | 2.8 | energy production, nucleic acid metabolism, small biochemistry, metabolic disorder, developmental disorder, hereditary disorder |
| 269 | A_33_P3257703 | C9orf131 | chromosome 9 open reading frame 131, transcript variant 1 | 2.7 | und |
| 270 | A_33_P3802146 |  | cDNA FLJ16301 fis, clone PLACE7000333 | 2.7 | und |
| 271 | A_19_P00327297 | XLOC_008015 | lincRNA (XLOC_008015) | 2.7 | und |
| 272 | A_23_P90062 | DNAJB1 | DnaJ (Hsp40) homolog, subfamily B, member 1 | 2.7 | cell death and survival, organismal development, gene expression, DNA replication, recombination, and repair, organismal survival, cellular growth and proliferation, |
| 273 | A_33_P3353343 | SRRM2 | serine/arginine repetitive matrix 2 | 2.7 | infectious disease |
| 274 | A_24_P167877 | LOC100132247 | nuclear pore complex interacting protein related gene | 2.7 | und |
| 275 | A_33_P3369419 | GON4L | gon-4-like (C. elegans), transcript variant 2 | 2.7 | und |
| 276 | A_24_P403244 | PILRB | paired immunoglobin-like type 2 receptor beta, transcript variant 3 | 2.7 | und |
| 277 | A_19_P00802937 |  | lincRNA:chr4:88813780-88814117 reverse strand | 2.7 | und |
| 278 | A_33_P3380837 | AMZ1 | archaelysin family metallopeptidase 1 | 2.7 | und |
| 279 | A_33_P3645805 |  | cDNA FLJ11735 fis, clone HEMBA1005447 | 2.7 | und |
| 280 | A_19_P00327081 | XLOC_l2_001273 | lincRNA (XLOC_l2_001273) | 2.7 | und |
| 281 | A_33_P3273719 | ELOVL5 | ELOVL fatty acid elongase 5, transcript variant 4 | 2.7 | small molecule biochemistry, lipid metabolism, molecular transport, carbohydrate metabolism |
| 282 | A_23_P408353 | HLA-A | major histocompatibility complex, class I, A , transcript variant 1 | 2.7 | cell death and survival |
| 283 | A_24_P625382 | CSDA | cold shock domain protein A , transcript variant 1 | 2.7 | cell death and survival, embryonic development, organismal development, gene expression, organismal survival, cellular growth and proliferation |
| 284 | A_24_P323545 | MYH14 | myosin, heavy chain 14, non-muscle, transcript variant 1 | 2.7 | cellular assembly and organization, energy production, DNA replication, recombination, and repair, cell cycle, cellular growth and proliferation, cellular movement |
| 285 | A_23_P156445 | DDX43 | DEAD (Asp-Glu-Ala-Asp) box polypeptide 43 | 2.7 | und |
| 286 | A_33_P3702281 | LOC284014 | cDNA FLJ38248 fis, clone FCBBF2007556 | 2.7 | und |
| 287 | A_23_P162300 | IRAK3 | interleukin-1 receptor-associated kinase 3, transcript variant 1 | 2.7 | cell death and survival, organismal development, organismal survival, infectious disease, inflammatory response, post-translational modification |
| 288 | A_23_P25224 | CSDA | cold shock domain protein A, transcript variant 1 | 2.6 | cell death and survival, embryonic development, organismal development, organismal survival, cellular growth and proliferation, neurological disease |
| 289 | A_33_P3800664 |  | cDNA FLJ33330 fis, clone BRACE2000441 | 2.6 | und |
| 290 | A_33_P3365701 |  | Q7SFQ1_NEUCR Predicted protein, partial (13%) | 2.6 | und |
| 291 | A_33_P3380405 | PSCD1 | cDNA FLJ41900 fis, clone OCBBF3000483 | 2.6 | und |
| 292 | A_33_P3259403 | PAN3 | PAN3 poly(A) specific ribonuclease subunit homolog (S. cerevisiae) | 2.6 | und |
| 293 | A_32_P806841 | ARL4A | ADP-ribosylation factor-like 4A, transcript variant 1 | 2.6 | und |
| 294 | A_33_P3310070 | FOXK2 | forkhead box K2 (FOXK2) | 2.6 | und |
| 295 | A_23_P213863 | FLJ10404 | family with sequence similarity 193, member B, transcript variant 3 | 2.6 | und |
| 296 | A_33_P3236267 | REXO1L1 | REX1, RNA exonuclease 1 homolog (S. cerevisiae)-like 1 (REXO1L1) | 2.6 | und |
| 297 | A_33_P3387901 | SLC25A19 | solute carrier family 25 (mitochondrial thiamine pyrophosphate carrier), member 19, nuclear gene encoding mitochondrial protein, transcript variant 1 | 2.6 | cell death and survival, embryonic development, organismal development, tissue development, cellular assembly and organization, cellular function and maintenance |
| 298 | A_33_P3349474 |  | ankyrin repeat and SOCS box containing 3 | 2.6 | und |
| 299 | A_23_P342668 | SFRS17A | A kinase (PRKA) anchor protein 17A (AKAP17A), transcript variant 1 | 2.6 | und |
| 300 | A_33_P3451157 |  | cDNA FLJ30418 fis, clone BRACE2008754 | 2.5 | und |
| 301 | A_33_P3397955 | C14orf85 | ITPK1 antisense RNA 1 (non-protein coding) | 2.5 | und |
| 302 | A_23_P208334 | PDE4A | phosphodiesterase 4A, cAMP-specific, transcript variant 4 | 2.5 | cell death and survival, nuclear acid, small molecule biochemistry, organismal injury and abnormalities, infectious disease, neurological disease |
| 303 | A_23_P30315 | TRIM7 | tripartite motif containing 7, transcript variant 6 | 2.5 | infectious disease |
| 304 | A_23_P3574 | GFOD2 | glucose-fructose oxidoreductase domain containing 2, transcript variant 1 | 2.5 | und |
| 305 | A_19_P00318861 |  | lincRNA:chr7:66370580-66371287 forward strand | 2.5 | und |
| 306 | A_33_P3219720 | ZNF248 | zinc finger protein 248 | 2.5 | und |
| 307 | A_23_P73150 | TTC25 | tetratricopeptide repeat domain 25 | 2.5 | und |
| 308 | A_33_P3404097 | PGM5P2 | phosphoglucomutase 5 pseudogene 2 | 2.5 | und |
| 309 | A_23_P59349 | HECA | headcase homolog (Drosophila) | 2.5 | und |
| 310 | A_33_P3237760 |  | ZSWM1_HUMAN (Q9BR11) Zinc finger SWIM domain-containing protein 1, partial (79%) | 2.5 | und |
| 311 | A_33_P3318292 | SFPQ | splicing factor proline/glutamine-rich | 2.5 | gene expression, infectious disease, cancer |
| 312 | A_33_P3374952 | KLF17 | Kruppel-like factor 17 (KLF17) | 2.5 | gene expression |
| 313 | A_23_P87575 | CCNT1 | cyclin T1 | 2.5 | cell death and survival, gene expression, infectious disease, post-translational modification |
| 314 | A_33_P3346403 | PTMA | prothymosin, alpha, transcript variant 1 | 2.5 | cell death and survival, gene expression, cell cycle, cellular development, cellular growth and differentiation, connective tissue development |
| 315 | A_19_P00325352 |  | lincRNA:chr15:64753847-64771422 forward strand | 2.5 | und |
| 316 | A_33_P3266928 | LMTK3 | lemur tyrosine kinase 3 | 2.5 | und |
| 317 | A_33_P3371752 | EPS15 | epidermal growth factor receptor pathway substrate 15 | 2.5 | infectious disease, cellular growth and proliferation, molecular transport, connective tissue, protein trafficking |
| 318 | A_23_P251232 | TTTY14 | testis-specific transcript, Y-linked 14 (non-protein coding) | 2.5 | und |
| 319 | A_23_P110879 | TRAF3IP2 | TRAF3 interacting protein 2, transcript variant 2 | 2.4 | cell death and survival, gene expression, tissue morphology, organ morphology, cancer, immunological disease |
| 320 | A_33_P3342807 |  | ALU1_HUMAN (P39188) Alu subfamily J sequence contamination warning entry, partial (10%) | 2.4 | und |
| 321 | A_33_P3212716 |  | cDNA FLJ46080 fis, clone TESTI2004971 | 2.4 | und |
| 322 | A_19_P00801911 |  | lincRNA:chr7:35664275-35671050 reverse strand | 2.4 | und |
| 323 | A_23_P212639 | SFRS10 | transformer 2 beta homolog (Drosophila), transcript variant 1 | 2.4 | und |
| 324 | A_19_P00810181 |  | lincRNA:chr6:64262816-64269341 forward strand | 2.4 | und |
| 325 | A_23_P64689 | PAN2 | PAN2 poly(A) specific ribonuclease subunit homolog (S. cerevisiae), transcript variant 3 | 2.4 | und |
| 326 | A_23_P12784 | FRAT2 | frequently rearranged in advanced T-cell lymphomas 2 | 2.4 | und |
| 327 | A_19_P00810474 | LOC100652741 | protein capicua homolog | 2.4 | und |
| 328 | A_33_P3307894 |  | Q7Z5D8_HUMAN (Q7Z5D8) Homeobox C14 protein, partial (14%) | 2.4 | und |
| 329 | A_23_P160992 | FMO4 | flavin containing monooxygenase 4 | 2.4 | und |
| 330 | A_33_P3310232 | L2HGDH | L-2-hydroxyglutarate dehydrogenase, nuclear gene encoding mitochondrial protein | 2.4 | cell death and survival, metabolic disorder, renal and urological disease, dermatological disease, hereditary disorder |
| 331 | A_33_P3239317 | LOC400752 | uncharacterized LOC400752 | 2.4 | und |
| 332 | A_24_P207479 | DEDD2 | death effector domain containing 2 | 2.4 | cell death and survival |
| 333 | A_23_P434301 | PTMA | prothymosin, alpha, transcript variant 2 | 2.4 | cell death and survival, gene expression, cell cycle, cellular development, cellular growth and differentiation, connective tissue development |
| 334 | A_24_P246467 | ATF2 | activating transcription factor 2 | 2.4 | cell death and survival, embryonic development, organismal development, tissue and organ morphology, cellular development, growth and differentiation, organismal survival |
| 335 | A_23_P166536 | BRD1 | bromodomain containing 1 | 2.4 | cell death and survival, embryonic development, organismal development, tissue development, cellular assembly and organization |
| 336 | A_24_P402836 | ZNF141 | zinc finger protein 141 | 2.4 | und |
| 337 | A_23_P141636 | EIF4A3 | eukaryotic translation initiation factor 4A3 | 2.4 | gene expression, infectious disease |
| 338 | A_19_P00317575 |  | lincRNA:chrX:39696789-39707612 reverse strand | 2.4 | und |
| 339 | A_19_P00322968 | LOC100506190 | uncharacterized LOC100506190 | 2.4 | und |
| 340 | A_33_P3284408 | DDI2 | DNA-damage inducible 1 homolog 2 (S. cerevisiae) | 2.4 | und |
| 341 | A_33_P3760125 |  | non-protein coding RNA 230B | 2.4 | und |
| 342 | A_23_P147439 | ATXN2L | ataxin 2-like (ATXN2L), transcript variant E | 2.4 | und |
| 343 | A_33_P3302681 | TLE4 | transducin-like enhancer of split 4 (E(sp1) homolog, Drosophila) | 2.4 | gene expression, cellular growth and proliferation |
| 344 | A_33_P3328292 | PLAGL2 | pleiomorphic adenoma gene-like 2 | 2.4 | cell death and survival, organismal development, gene expression, cell cycle, cellular development, growth and differentiation |
| 345 | A_33_P3336514 |  | Uncharacterized protein | 2.4 | und |
| 346 | A_33_P3244998 | ODF2L | outer dense fiber of sperm tails 2-like, transcript variant 2 | 2.4 | und |
| 347 | A_19_P00805327 |  | lincRNA:chr6:84676486-84677143 forward strand | 2.3 | und |
| 348 | A_19_P00813409 |  | lincRNA:chr7:66366065-66371465 forward strand | 2.3 | und |
| 349 | A_19_P00329121 |  | lincRNA:chr1:36522963-36529063 forward strand | 2.3 | und |
| 350 | A_33_P3549874 | LOC255177 | cDNA clone IMAGE:5295221 | 2.3 | und |
| 351 | A_19_P00805047 | NASP | nuclear autoantigenic sperm protein (histone-binding), transcript variant 2] | 2.3 | embryonic development, organismal development, cell cycle, organismal survival, cell growth and proliferation |
| 352 | A_23_P422212 | SLC35F3 | solute carrier family 35, member F3 | 2.3 | und |
| 353 | A_23_P162782 | ARGLU1 | arginine and glutamate rich 1 | 2.3 | infectious disease |
| 354 | A_33_P3274528 | C9orf153 | chromosome 9 open reading frame 153 | 2.3 | und |
| 355 | A_33_P3248072 | SIRPB1 | signal-regulatory protein beta 1, transcript variant 3 | 2.3 | und |
| 356 | A_33_P3220437 | TNFAIP8L1 | tumor necrosis factor, alpha-induced protein 8-like 1, transcript variant 1 | 2.3 | cell death and survival |
| 357 | A_33_P3422429 | INADL | InaD-like (Drosophila) | 2.3 | und |
| 358 | A_24_P941441 | GNA13 | guanine nucleotide binding protein (G protein), alpha 13 | 2.3 | cell death and survival, embryonic development, organismal development, tissue development, cellular assembly and organization, cell function and maintenance, cell cycle, organismal survival |
| 359 | A_23_P218158 | SMEK1 | SMEK homolog 1, suppressor of mek1 (Dictyostelium) | 2.3 | und |
| 360 | A_33_P3344264 | DUS3L | dihydrouridine synthase 3-like (S. cerevisiae), transcript variant 1 | 2.3 | und |
| 361 | A_33_P3323929 | FLJ40536 | cDNA FLJ40536 fis, clone TESTI2047930 | 2.3 | und |
| 362 | A_23_P83917 | FBXL11 | lysine (K)-specific demethylase 2A , transcript variant 1 | 2.3 | und |
| 363 | A_33_P3408221 | LOC729799 | SEC14-like 1 pseudogene | 2.3 | und |
| 364 | A_33_P3233981 | NASP | nuclear autoantigenic sperm protein (histone-binding), transcript variant 2 | 2.3 | embryonic development, organismal development, cell cycle, organismal survival, cellular growth and proliferation |
| 365 | A_33_P3325671 | LOC100132731 | Uncharacterized protein FLJ43738 | 2.3 | und |
| 366 | A_19_P00804072 | RNF213 | ring finger protein 213, transcript variant 1 | 2.3 | und |
| 367 | A_33_P3350306 | ZNF589 | zinc finger protein 589 | 2.3 | und |
| 368 | A_23_P201483 | MAPKAPK2 | mitogen-activated protein kinase-activated protein kinase 2, transcript variant 1 | 2.3 | cell death and survival, organismal development, gene expression, organismal survival, cellular morphology, cellular development, growth and differentiation |
| 369 | A_33_P3384442 | LAMA5 | laminin, alpha 5 | 2.2 | cell death and survival, embryonic development, tissue development and morphology, cell to cell signaling and interaction, cell growth and differentiation, cell movement |
| 370 | A_33_P3231005 |  |  | 2.2 | und |
| 371 | A_23_P38723 | SMCHD1 | structural maintenance of chromosomes flexible hinge domain containing 1 | 2.2 | und |
| 372 | A_33_P3365878 | BMP8B | bone morphogenetic protein 8b | 2.2 | cell death and survival, tissue development, reproductive system disease |
| 373 | A_23_P34510 | PHC2 | polyhomeotic homolog 2 (Drosophila), transcript variant 1 | 2.2 | embryonic development, organismal development, tissue development, cell cycle, organ development, organismal survival, cellular development growth and differentiation, |
| 374 | A_19_P00325334 |  | lincRNA:chr7:126990389-127005914 reverse strand | 2.2 | und |
| 375 | A_33_P3265956 | CBFA2T2 | core-binding factor, runt domain, alpha subunit 2; translocated to, 2 | 2.2 | organismal development, organismal survival, |
| 376 | A_24_P150874 | GNA13 | guanine nucleotide binding protein (G protein), alpha 13 | 2.2 | cell death and survival, embryonic development, organismal development, tissue development, cellular assembly and organization, cellular function and maintenance, cell cycle, organismal survival |
| 377 | A_33_P3224483 | RABL2B | RAB, member of RAS oncogene family-like 2B, mRNA (cDNA clone IMAGE:3902486), with apparent retained intron | 2.2 | und |
| 378 | A_33_P3316686 | CYB5R4 | cytochrome b5 reductase 4 | 2.2 | nucleic acid metabolism, small molecule biochemistry, molecular transport, protein synthesis |
| 379 | A_19_P00321306 | RNF213 | ring finger protein 213, transcript variant 1 | 2.2 | und |
| 380 | A_19_P00320819 |  | lincRNA:chr7:22895704-22896694 forward strand | 2.2 | und |
| 381 | A_33_P3270009 | NCOA1 | nuclear receptor coactivator 1, transcript variant 2 | 2.2 | cell death and survival, embryonic development, organismal development, tissue development, cellular assembly and organization, organismal survival |
| 382 | A_33_P3416301 | E2F6 | E2F transcription factor 6 | 2.2 | cell death and survival, organismal development, gene expression, cell cycle, organismal survival, cell growth and proliferation, cancer |
| 383 | A_19_P00328788 |  | lincRNA:chr6:56809416-56818656 forward strand | 2.2 | und |
| 384 | A_23_P61180 | PLCXD1 | phosphatidylinositol-specific phospholipase C, X domain containing 1, transcript variant 1 | 2.2 | und |
| 385 | A_23_P14649 | C15orf28 | ANP32A intronic transcript 1 (non-protein coding) (ANP32A-IT1) | 2.2 | und |
| 386 | A_23_P135031 | C9orf4 | chromosome 9 open reading frame 4 | 2.2 | und |
| 387 | A_33_P3499692 | LOC645261 | PP565 mRNA, complete cds | 2.2 | und |
| 388 | A_23_P206960 | SEC14L1 | SEC14-like 1 (S. cerevisiae), transcript variant 1 | 2.2 | infectious disorder |
| 389 | A_33_P3264780 | CDK8 | cyclin-dependent kinase 8 | 2.2 | cell death and survival, gene expression, cell cycle, post-translational modification |
| 390 | A_33_P3316293 | CAB39 | calcium binding protein 39, transcript variant 2 | 2.2 | post-translational modification |
| 391 | A_33_P3317211 | MECP2 | methyl CpG binding protein 2 (Rett syndrome), transcript variant 2 | 2.2 | cell death and survival, organismal development, cellular assembly and organization, cell cycle, organ development, organismal survival |
| 392 | A_19_P00809776 |  | lincRNA:chr6:126419657-126440982 forward strand | 2.2 | organismal developmental, cellular assembly and organization, nervous system development, tissue morphology, organ development and morphology, organismal survival |
| 393 | A_23_P6303 | U2AF1 | U2 small nuclear RNA auxiliary factor 1, transcript variant c | 2.2 | hematological disease, cancer |
| 394 | A_19_P00802417 | P39195 | ALU8_HUMAN (P39195) Alu subfamily SX sequence contamination warning entry, partial (20%) | 2.1 | und |
| 395 | A_33_P3888365 | RSBN1 | round spermatid basic protein 1 | 2.1 | und |
| 396 | A_33_P3221563 | ARMC5 | armadillo repeat containing 5 , transcript variant 2 | 2.1 | und |
| 397 | A_33_P3301915 | USP51 | ubiquitin specific peptidase 51 | 2.1 | und |
| 398 | A_33_P3298440 | hCG_1986447 | cDNA FLJ31019 fis, clone HLUNG2000362 | 2.1 | und |
| 399 | A_19_P00317566 |  | lincRNA:chr7:22896146-22900458 forward strand | 2.1 | und |
| 400 | A_24_P542375 | PTMA | prothymosin, alpha , transcript variant 2 | 2.1 | cell death and survival, gene expression, cell cycle, cellular development, growth and proliferation, connective tissue development and function |
| 401 | A_24_P320254 | HNRNPH1 | heterogeneous nuclear ribonucleoprotein H1 (H) | 2.1 | infectious disease, RNA post-transcriptional modification |
| 402 | A_23_P142421 | MLL4 | myeloid/lymphoid or mixed-lineage leukemia 4 | 2.1 | cell death and survival, embryonic development, organismal development, cellular assembly and organization, tissue development, development disorder |
| 403 | A_24_P97001 | PRPF38A | PRP38 pre-mRNA processing factor 38 (yeast) domain containing A | 2.1 | infectious disease |
| 404 | A_33_P3351934 | LOC100129405 | misato homolog 2 pseudogene | 2.1 | und |
| 405 | A_33_P3302791 | LOC730338 | cDNA FLJ43321 fis, clone NT2RI2027396 | 2.1 | und |
| 406 | A_19_P00331395 |  | lincRNA:chr13:112169174-112181374 forward strand | 2.1 | und |
| 407 | A_33_P3239267 | C3orf54 | chromosome 3 open reading frame 54 | 2.1 | und |
| 408 | A_24_P170763 | KIAA0323 | KH and NYN domain containing (KHNYN) | 2.1 | und |
| 409 | A_24_P400473 | SLC25A44 | solute carrier family 25, member 44 | 2.1 | und |
| 410 | A_23_P54116 | DAAM1 | dishevelled associated activator of morphogenesis 1 | 2.1 | und |
| 411 | A_23_P15511 | DDX5 | DEAD (Asp-Glu-Ala-Asp) box polypeptide 5 | 2.1 | cell death and survival, gene expression, organismal development, infectious disease, cellular growth and proliferation, organismal survival |
| 412 | A_23_P90311 | TICAM1 | toll-like receptor adaptor molecule 1 | 2.1 | cell death and survival, gene expression, infectious disease, cellular growth and proliferation, hematological system development and function |
| 413 | A_33_P3369696 | CCDC94 | coiled-coil domain containing 94 | 2.1 | und |
| 414 | A_19_P00808297 |  | lincRNA:chr2:179375079-179386329 forward strand | 2.1 | und |
| 415 | A_33_P3343428 | TRAF3IP2 | TRAF3 interacting protein 2, transcript variant 2 | 2.1 | cell death and survival |
| 416 | A_23_P89422 | ABCA10 | ATP-binding cassette, sub-family A (ABC1), member 10 | 2.1 | und |
| 417 | A_19_P00329664 |  | lincRNA:chr15:79198170-79208745 forward strand | 2.1 | und |
| 418 | A_24_P752208 |  |  | 2.1 | und |
| 419 | A_19_P00327772 | XLOC_l2_000864 | lincRNA (XLOC_l2_000864), lincRNA | 2.1 |  |
| 420 | A_23_P382602 | BCL9 | B-cell CLL/lymphoma 9 | 2.1 | und |
| 421 | A_24_P188377 | CD55 | CD55 molecule, decay accelerating factor for complement (Cromer blood group), transcript variant 1 | 2.1 | cell death and survival, tissue development |
| 422 | A_23_P93629 | TRIM24 | tripartite motif containing 24, transcript variant 1 | 2.0 | cell death and survival, cell cycle |
| 423 | A_33_P3378920 | RBM14-RBM4 | RBM14-RBM4 readthrough, transcript variant 1 | 2.0 | und |
| 424 | A_23_P27381 | TSHZ1 | teashirt zinc finger homeobox 1 | 2.0 | und |
| 425 | A_23_P131646 | RPIA | ribose 5-phosphate isomerase A | 2.0 | und |
| 426 | A_24_P91701 | LOC51233 | chromosome 22 open reading frame 43 | 2.0 | und |
| 427 | A_24_P135276 | USP42 | ubiquitin specific peptidase 42 | 2.0 | und |
| 428 | A_33_P3290296 | LOC100130175 | cDNA FLJ43841 fis, clone TESTI4006137 | 2.0 | und |
| 429 | A_32_P225854 | SPRED2 | sprouty-related, EVH1 domain containing 2, transcript variant 1 | 2.0 | organismal development, organismal survival |
| 430 | A_23_P120428 | C20orf10 | TP53 target 5 | 2.0 | und |
| 431 | A_19_P00320843 |  | lincRNA:chr7:66369626-66371283 forward strand | 2.0 | und |
| 432 | A_24_P267664 | C21orf88 | chromosome 21 open reading frame 88, transcript variant 2 | 2.0 | und |
| 433 | A_33_P3223660 | C9orf130 | long intergenic non-protein coding RNA 476, transcript variant 2 | 2.0 | und |
| 434 | A_33_P3334248 | FBRS | fibrosin | 2.0 | und |
| 435 | A_33_P3416448 | BTBD7 | BTB (POZ) domain containing 7 (BTBD7), transcript variant 1, mRNA [NM_001002860] | 2.0 | und |
