# Supplementary material for: Awakened by Cellular Stress: Isolation and Characterization of a Novel Population of Pluripotent Stem Cells Derived from Human Adipose Tissue
Source: PLoS One. 2013 Jun 5;8(6):e64752. doi: 10.1371/journal.pone.0064752 (PMC3673968; doi:10.1371/journal.pone.0064752)
Supplement: Table S2 — GO Analysis of the down-regulated genes in Muse-AT vs ASCs with 2 fold changes and p<0.05. (DOC) [file pone.0064752.s002.doc]

**Supplemental Data: Table 2**

**GO Analysis of the down-regulated genes in Muse-AT vs ASCs with 2 fold changes and p < 0.05**

| No | Probe Name | Gene Symbol | Description | Fold change [ave MUSE] vs[ave ASC] | Function |
| --- | --- | --- | --- | --- | --- |
| 1 | A_24_P943894 | SCUBE3 | signal peptide, CUB domain, EGF-like 3 | 253.8 | cellular development, cellular growth and proliferation, cellular movement, cancer, protein synthesis, |
| 2 | A_24_P342632 | AK5 | adenylate kinase 5, transcript variant 1 | 136.6 | cellular death and survival, nucleic acid metabolism, small molecular biochemistry |
| 3 | A_23_P17593 | CDH4 | cadherin 4, type 1, R-cadherin (retinal), transcript variant 1 | 117.3 | cellular growth and proliferation |
| 4 | A_24_P40626 | GREM2 | gremlin 2 | 115.2 | und |
| 5 | A_23_P115872 | CEP55 | centrosomal protein 55kDa, transcript variant 1 | 93.6 | cell cycle, cellular movement |
| 6 | A_23_P163481 | BUB1B | budding uninhibited by benzimidazoles 1 homolog beta (yeast) | 66.4 | cellular death and survival, cellular assembly and organization, cell cycle, cellular growth and proliferation, post-translational modifications |
| 7 | A_24_P218979 | CDCA3 | cell division cycle associated 3 | 62.5 | und |
| 8 | A_19_P00802201 |  |  | 59.3 |  |
| 9 | A_33_P3326210 | ESCO2 | establishment of cohesion 1 homolog 2 (S. cerevisiae) | 56.8 | cell cycle, cellular movement, dermatological diseases and conditions, development disorder, hereditary disorder, cardiovascular disease |
| 10 | A_23_P74349 | NUF2 | NDC80 kinetochore complex component, homolog (S. cerevisiae) | 54.8 | cellular death and survival, cellular assembly and organization, cell cycle |
| 11 | A_23_P200310 | DEPDC1 | DEP domain containing 1 , transcript variant 2 | 52.7 | cellular death and survival |
| 12 | A_33_P3311755 | KIF23 | kinesin family member 23, transcript variant 1 | 52.0 | cellular assembly and organization, cellular function and maintainance, DNA replication, recombination, and repair, cell cycle, cellular movement |
| 13 | A_23_P416395 | STC2 | stanniocalcin 2 | 39.5 | organismal development, cellular growth and proliferation |
| 14 | A_23_P108673 | TMEM166 | family with sequence similarity 176, member A, transcript variant 2 | 38.2 | und |
| 15 | A_32_P157945 | DSP | desmoplakin, transcript variant 1 | 37.7 | cellular death and survival, embryonic development, organismal development, cellular assembly and organization, tissue morphology, organismal survival |
| 16 | A_23_P132760 | TRH | thyrotropin-releasing hormone | 35.1 | cell death and survival, nucleic acid metabolism, small molecule biochemistry, nervous system development, cell morphology, cell to cell signaling and interaction |
| 17 | A_23_P253524 | CENPE | centromere protein E, 312kDa | 30.6 | cellular assembly and organization, cell cycle, cellular compromise, hepatic system disease |
| 18 | A_23_P150935 | TROAP | trophinin associated protein (tastin), transcript variant 1 | 28.3 | und |
| 19 | A_23_P29723 | SGOL1 | shugoshin-like 1 (S. pombe), transcript variant A2 | 26.6 | cellular assembly and organization, cellular maintenance and function, cell cycle |
| 20 | A_23_P206059 | PRC1 | protein regulator of cytokinesis 1, transcript variant 1 | 25.0 | cellular assembly and organization, cellular function and maintainance, DNA replication, recombination, and repair, cell cycle, cellular movement |
| 21 | A_32_P72341 | TRIM59 | tripartite motif containing 59 | 23.9 | und |
| 22 | A_23_P118246 | GINS2 | GINS complex subunit 2 (Psf2 homolog) | 18.6 | und |
| 23 | A_33_P3397443 | PKMYT1 | protein kinase, membrane associated tyrosine/threonine 1, transcript variant 2 | 17.4 | cell death and survival, cell cycle, infectious disease, pos-translational modification, DNA replication, recombination and repair, cell cylce |
| 24 | A_24_P156490 | KCNMA1 | potassium large conductance calcium-activated channel, subfamily M, alpha member 1, transcript variant 2 | 15.9 | cell death and survival, organismal development, cell cycle, nervous system development and function, organismal survival, cellular growth and proliferation |
| 25 | A_23_P1331 | COL13A1 | collagen, type XIII, alpha 1, transcript variant 5 | 15.2 | neurological disease, developmental disorder |
| 26 | A_23_P211504 | KDELR3 | KDEL (Lys-Asp-Glu-Leu) endoplasmic reticulum protein retention receptor 3, transcript variant 2 | 14.8 | und |
| 27 | A_33_P3313075 |  |  | 14.0 | und |
| 28 | A_33_P3418833 | FLRT3 | fibronectin leucine rich transmembrane protein 3, transcript variant 2 | 13.8 | developmental disorder |
| 29 | A_33_P3368049 | LOC643988 | unlocalized LOC643988 | 13.5 | und |
| 30 | A_32_P4626 | LOC100505880 | hypothetical LOC100505880, transcript variant 1 | 13.3 | und |
| 31 | A_33_P3260575 | CERCAM | cerebral endothelial cell adhesion molecule | 12.5 | und |
| 32 | A_24_P876522 | LOC493869 | glutathione peroxidase 8 (putative) | 12.3 | und |
| 33 | A_23_P76245 | SCN8A | sodium channel, voltage gated, type VIII, alpha subunit, transcript variant 1 | 11.9 | molecular transport, neurological disease |
| 34 | A_33_P3402868 | GRIN2D | glutamate receptor, ionotropic, N-methyl D-aspartate 2D | 11.6 | cell death and survival |
| 35 | A_23_P207400 | BRCA1 | breast cancer 1, early onset , transcript variant 2 | 8.7 | cellular death and survival, embryonic development, organismal development, tissue development, cellular assembly and organization, cellular function and maintenance, cell cycle, organismal survival |
| 36 | A_23_P307502 | C9orf53 | chromosome 9 open reading frame 53 | 7.9 | und |
| 37 | A_23_P30363 | P4HA2 | prolyl 4-hydroxylase, alpha polypeptide II, transcript variant 1 | 7.9 | und |
| 38 | A_23_P366366 | SCRN1 | secernin 1 , transcript variant 2 | 7.9 | und |
| 39 | A_24_P277934 | COL1A2 | collagen, type I, alpha 2 | 7.7 | cellular growth and proliferation, |
| 40 | A_23_P307328 | WHSC1 | Wolf-Hirschhorn syndrome candidate 1, transcript variant 8 | 7.6 | und |
| 41 | A_33_P3253304 | LOC100270710 | uncharacterized LOC100270710 | 7.4 | und |
| 42 | A_23_P75071 | MPHOSPH1 | kinesin family member 20B | 7.3 | und |
| 43 | A_19_P00812135 | XLOC_000670 | BROAD Institute lincRNA (XLOC_000670), lincRNA [TCONS_00002277] | 7.3 | und |
| 44 | A_23_P58396 | PDGFC | platelet derived growth factor C , transcript variant 1 | 7.2 | embryonic development, organismal development, tissue development, cellular assembly and organization, organ development, organismal survival |
| 45 | A_19_P00318057 |  | lincRNA:chr4:15670037-15683240 reverse strand | 6.9 | und |
| 46 | A_23_P12363 | ROR1 | receptor tyrosine kinase-like orphan receptor 1, transcript variant 1 | 6.3 | cellular death and survival, cell growth and proliferation, cancer, neurological disease, post-translational modification,developmental disorder |
| 47 | A_33_P3240787 | LOC100131910 | hypothetical LOC100131910 | 6.2 | und |
| 48 | A_33_P3323847 | RECQL4 | RecQ protein-like 4 | 6.2 | organismal development, metabolic disorder, organ morphology, organismal injury and abnormalities, organismal survival, cellular development, growth and proliferation |
| 49 | A_23_P89665 | KRT33B | keratin 33B | 5.9 | und |
| 50 | A_23_P65041 | RACGAP1P | Rac GTPase activating protein 1 pseudogene | 5.8 | und |
| 51 | A_23_P141715 | C18orf10 | chromosome 18 open reading frame 10 | 5.8 | und |
| 52 | A_23_P67339 | RCN3 | reticulocalbin 3, EF-hand calcium binding domain | 5.8 | und |
| 53 | A_23_P215634 | IGFBP3 | insulin-like growth factor binding protein 3, transcript variant 1 | 5.7 | cellular death and survival, embryonic development, organismal development, tissue development, cell cycle, organismal survival |
| 54 | A_23_P74299 | ADORA1 | adenosine A1 receptor, transcript variant 1 | 5.5 | cellular death and survival, embryonic development, organismal development, tissue development, cellular function and maintainance, organismal survival |
| 55 | A_24_P109644 |  | docking protein 1-like protein | 5.4 | und |
| 56 | A_23_P50591 | KCNK6 | potassium channel, subfamily K, member 6 | 5.4 | und |
| 57 | A_33_P3367855 | PIK3R2 | phosphoinositide-3-kinase, regulatory subunit 2 (beta) | 5.3 | cellular death and survival, small molecule biochemistry, infectious disease, cellular growth and proliferation, molecular transport, carbohydrate metabolism |
| 58 | A_19_P00315641 | XLOC_008079 |  | 5.2 | und |
| 59 | A_33_P3385957 | TTLL1 | tubulin tyrosine ligase-like family, member 1, transcript variant 1 | 5.2 | cellular death and survival, organismal development |
| 60 | A_23_P146830 | SLC25A10 | mitochondrial carrier; dicarboxylate transporter, member 10, nuclear gene encoding mitochondrial protein | 5.0 | cellular death and survival, embryonic development, organismal development, tissue development, cellular assembly and organization, cellular maintenance and function, |
| 61 | A_33_P3325843 | FLJ40039 | cDNA FLJ40039 fis, clone SYNOV2000397 | 5.0 | und |
| 62 | A_23_P102706 | SNPH | syntaphilin | 4.9 | cellular assembly and organization, cellular function and maintainance, cellular development, cellular growth and proliferation |
| 63 | A_23_P153628 | YIF1B | Yip1 interacting factor homolog B (S. cerevisiae) (YIF1B), transcript variant 2 | 4.8 | und |
| 64 | A_24_P60441 | EVC | Ellis van Creveld syndrome | 4.8 | und |
| 65 | A_23_P72961 | PRPS1 | phosphoribosyl pyrophosphate synthetase 1, transcript variant 14 | 4.8 | nucleic acid metabolism, small molecule biochemistry, metabolic disease, infectious disease, skeletical and muscular disorders, neurological disorders |
| 66 | A_23_P135499 | CLIC4 | chloride intracellular channel 4 (CLIC4), nuclear gene encoding mitochondrial protein | 4.7 | cellular death and survival, embryonic development, organismal development, tissue development, cellular function and maintenance, organ development, organismal survival |
| 67 | A_23_P132226 | TPST2 | tyrosylprotein sulfotransferase 2, transcript variant 1 | 4.7 | organismal development |
| 68 | A_23_P214950 | PERP | PERP, TP53 apoptosis effector | 4.7 | cellular death and survival, organismal development, tissue development, dermatological disease and conditions, organismal survival |
| 69 | A_19_P00322929 | XLOC_003870 | hypothetical LOC100507376, transcript variant 2 | 4.7 | und |
| 70 | A_33_P3298024 | ABCC3 | ATP-binding cassette, sub-family C (CFTR/MRP), member 3 (ABCC3), transcript variant 2 | 4.7 | cell death and survival |
| 71 | A_23_P152305 | CDH11 | cadherin 11, type 2, OB-cadherin (osteoblast) | 4.7 | embryonic development, organismal development, tissue development, organ development, organismal injuries and abnormalities, cell morphology, cellular growth and proliferation |
| 72 | A_33_P3423570 | METRN | meteorin, glial cell differentiation regulator | 4.7 | und |
| 73 | A_23_P88740 | CENPN | centromere protein N, transcript variant 3 | 4.6 | und |
| 74 | A_23_P356021 | FANCB | Fanconi anemia, complementation group B, transcript variant 1 | 4.6 | hematological disease |
| 75 | A_23_P353574 | NEK7 | NIMA (never in mitosis gene a)-related kinase 7 | 4.6 | cellular death and survival, cell cycle, lopment, cellular movement, post translational modification, development disorder |
| 76 | A_24_P157156 | CCDC150 | coiled-coil domain containing 150 | 4.6 | und |
| 77 | A_24_P128524 | ICMT | isoprenylcysteine carboxyl methyltransferase | 4.6 | cellular death and survival, embryonic development, organismal development, organismal survival, cellular development, cellular growth and proliferation |
| 78 | A_23_P312536 | ARSK | arylsulfatase family, member K | 4.5 | und |
| 79 | A_24_P404245 | PCYT2 | phosphate cytidylyltransferase 2, ethanolamine, transcript variant 2 | 4.5 | organismal development, organismal survival, organismal injury and abnormalities |
| 80 | A_33_P3262181 | APOBEC3F | apolipoprotein B mRNA editing enzyme, catalytic polypeptide-like 3F, transcript variant 2 | 4.5 | infectious disease, inflammatory response |
| 81 | A_23_P88630 | BLM | Bloom syndrome, RecQ helicase-like | 4.5 | cellular death and survival, embryonic development, organismal development, tissue development, cellular assembly and organization, cellular function and maintenance, DNA replication, recombination, and repair, cell cycle, organismal survival |
| 82 | A_23_P54000 | SNX6 | sorting nexin 6 , transcript variant 1 | 4.5 | gene expression, organismal survival, molecular transport, protein trafficking |
| 83 | A_23_P91430 | PXMP4 | peroxisomal membrane protein 4, 24kDa transcript variant 1 | 4.5 | und |
| 84 | A_33_P3393360 | LOC100130927 | cDNA FLJ42793 fis, clone BRAWH3007726 | 4.5 | und |
| 85 | A_33_P3213155 | psiTPTE22 | TPTE pseudogene | 4.4 | und |
| 86 | A_23_P9662 | IPP | intracisternal A particle-promoted polypeptide, transcript variant 1 | 4.3 | tissue development |
| 87 | A_33_P3224705 | C14orf135 | chromosome 14 open reading frame 135 | 4.3 | und |
| 88 | A_23_P100486 | VKORC1 | vitamin K epoxide reductase complex, subunit 1, transcript variant 2 | 4.3 | cellular growth and proliferation |
| 89 | A_33_P3230219 | TMEM54 | transmembrane protein 54 | 4.2 | und |
| 90 | A_23_P60227 | CCIN | calicin | 4.2 | und |
| 91 | A_23_P138725 | MARVELD1 | MARVEL domain containing 1 | 4.1 | und |
| 92 | A_23_P150365 | REXO2 | REX2, RNA exonuclease 2 homolog (S. cerevisiae), nuclear gene encoding mitochondrial protein | 4.1 | nucleic acid metabolism, small molecule biochemistry, RNA damage and repair |
| 93 | A_32_P196142 | LOC100130938 | hypothetical LOC100130938 | 4.1 | und |
| 94 | A_23_P96350 | PRAF2 | PRA1 domain family, member 2 | 4.1 | cellular death and survival, cellular growth and proliferation |
| 95 | A_23_P35609 | UROS | uroporphyrinogen III synthase | 4.1 | nuclear acid metabolism, hematological disease, metabolic disease, development disorder, hereditary disorder |
| 96 | A_33_P3292540 | CDKN2C | cyclin-dependent kinase inhibitor 2C (p18, inhibits CDK4), transcript variant 2 | 4.1 | cellular death and survival, organismal development, cell cycle, organ morphology, organismal injury and abnormalities, organismal survival |
| 97 | A_19_P00315452 | LOC100130938 | hypothetical LOC100130938 | 4.0 | und |
| 98 | A_33_P3271657 | HHIPL1 | HHIP-like 1, transcript variant 1 | 4.0 | und |
| 99 | A_32_P55860 | FAM33A | spindle and kinetochore associated complex subunit 2, transcript variant 1 | 4.0 | und |
| 100 | A_33_P3261828 | LOC390660 | mRNA for FLJ00317 protein | 4.0 | und |
| 101 | A_23_P143274 | NRSN2 | neurensin 2 | 4.0 | und |
| 102 | A_23_P138693 | NMT2 | N-myristoyltransferase 2 | 3.9 | und |
| 103 | A_23_P14193 | RFC3 | replication factor C (activator 1) 3, 38kDa, transcript variant 1 | 3.9 | DNA replication, recombination, and repair, enrgy production, nucelic acid metabolism, small molecule biochemistry |
| 104 | A_33_P3343145 | MAP1B | microtubule-associated protein 1B | 3.9 | cell death and survival, embryonic development, organismal development, tissue development, cellular assembly and organization, cellular function and maintenance, organ development and morphology, organismal survival |
| 105 | A_24_P366749 | ATP8B2 | ATPase, class I, type 8B, member 2, transcript variant 2 | 3.9 | und |
| 106 | A_19_P00329141 |  | lincRNA:chr1:54583637-54593837 reverse strand | 3.9 | und |
| 107 | A_23_P150407 | CREB3L1 | cAMP responsive element binding protein 3-like 1, mRNA [NM_052854] | 3.9 | cell death and survival, tissue development, gene expression, developmental disease |
| 108 | A_19_P00317776 |  | lincRNA:chr8:49330508-49333120 reverse strand | 3.9 | und |
| 109 | A_23_P89030 | C16orf95 | chromosome 16 open reading frame 95, transcript variant 1 | 3.9 | und |
| 110 | A_33_P3244249 | SH3TC2 | SH3 domain and tetratricopeptide repeats 2 [Source:HGNC Symbol;Acc:29427] [ENST00000513604] | 3.9 | lymphoid tissue structure and development, skeletal muscle disorder, neurological disorder |
| 111 | A_23_P24616 | SIAE | sialic acid acetylesterase, transcript variant 1 | 3.9 | cellular growth and proliferation |
| 112 | A_32_P175539 | RCN2 | reticulocalbin 2, EF-hand calcium binding domain | 3.8 | und |
| 113 | A_24_P244356 | NLRX1 | NLR family member X1, transcript variant 2 | 3.7 | organismal development, infectious disease, inflammatory disease, antimicrobial response |
| 114 | A_19_P00332245 |  | lincRNA:chr12:54523008-54559358 reverse strand | 3.7 | und |
| 115 | A_24_P81947 | CORO1C | coronin, actin binding protein, 1C, transcript variant 1 | 3.7 | und |
| 116 | A_23_P329890 | TMEM136 | transmembrane protein 136, transcript variant 2 | 3.7 | und |
| 117 | A_32_P221799 | HIST1H2AM | histone cluster 1, H2am | 3.7 | und |
| 118 | A_23_P29836 | TMEM42 | transmembrane protein 42 | 3.7 | und |
| 119 | A_23_P15714 | NSF | N-ethylmaleimide-sensitive factor, transcript variant 1 | 3.7 | cell death and survival, cellular assembly and organization, cellular function and maintenance, DNA replication, recombination, and repair, nucleic acid metabolism, nervous system development and function |
| 120 | A_33_P3400224 | DZIP1 | DAZ interacting protein 1, transcript variant 1 | 3.7 | und |
| 121 | A_23_P331670 | PYGB | phosphorylase, glycogen; brain | 3.6 | und |
| 122 | A_23_P208389 | AXL | AXL receptor tyrosine kinase, transcript variant 1 | 3.6 | cell death and survival, tissue development, cellular assembly and organization, cell cycle, organismal injury and abnormalities, cell morphology |
| 123 | A_33_P3281850 | CGREF1 | cell growth regulator with EF-hand domain 1 | 3.6 | infectious disease, cellular growth and proliferation |
| 124 | A_33_P3292679 | SNX6 | sorting nexin 6 , transcript variant 1 | 3.6 | gene expression, infectious disease, molecular transport, protein trafficking |
| 125 | A_33_P3387463 | ATP6V1D | ATPase, H+ transporting, lysosomal 34kDa, V1 subunit D | 3.6 | und |
| 126 | A_23_P35066 | SNX7 | sorting nexin 7, transcript variant 1 | 3.6 | cell death and survival |
| 127 | A_32_P135243 | MTHFD1L | methylenetetrahydrofolate dehydrogenase (NADP+ dependent) 1-like, nuclear gene encoding mitochondrial protein, transcript variant 2 | 3.6 | small molecule biochemistry |
| 128 | A_19_P00318645 | CRNDE | colorectal neoplasia differentially expressed (non-protein coding), transcript variant 1 | 3.5 | und |
| 129 | A_33_P3237835 |  |  | 3.5 | und |
| 130 | A_33_P3236102 | IER5L | immediate early response 5-like | 3.5 | und |
| 131 | A_32_P209094 | FGGY | FGGY carbohydrate kinase domain containing, transcript variant 2 | 3.5 | cell cycle |
| 132 | A_33_P3259092 | FKTN | fukutin (FKTN), transcript variant 1 | 3.5 | embryonic development, organismal development, tissue morphology, cellular growth and proliferation, skeletal and muscular disorders, neurological disease |
| 133 | A_33_P3390102 | GIPC1 | GIPC PDZ domain containing family, member 1, transcript variant 1 | 3.5 | cellular growth and proliferation, small molecule biochemistry, aminoacid metabolism |
| 134 | A_23_P170587 | SMYD2 | SET and MYND domain containing 2 | 3.4 | embryonic development, gene expression, cell morphology, cellular growth and proliferation |
| 135 | A_23_P53057 | ZNF215 | zinc finger protein 215 | 3.4 | und |
| 136 | A_23_P258621 | RTCD1 | RNA terminal phosphate cyclase domain 1, transcript variant 2 | 3.4 | und |
| 137 | A_32_P50066 | MAP9 | microtubule-associated protein 9 | 3.4 | cellular assembly and organization, cellular function and maintenance, cell cycle |
| 138 | A_23_P204980 | UGCGL2 | UDP-glucose glycoprotein glucosyltransferase 2 | 3.4 | und |
| 139 | A_33_P3375314 | ATP9A | ATPase, class II, type 9A | 3.4 | und |
| 140 | A_24_P945000 | FAM33A | spindle and kinetochore associated complex subunit 2, transcript variant 1 | 3.4 | und |
| 141 | A_24_P133162 | C1orf201 | chromosome 1 open reading frame 201, transcript variant 3 | 3.3 | und |
| 142 | A_33_P3380523 | PRTFDC1 | phosphoribosyl transferase domain containing 1 | 3.3 | und |
| 143 | A_23_P97853 | C10orf57 | chromosome 10 open reading frame 57 | 3.3 | und |
| 144 | A_19_P00809688 |  | lincRNA:chr16:1430124-1444874 reverse strand | 3.3 | und |
| 145 | A_24_P85511 | ANKRD26 | ankyrin repeat domain 26 | 3.3 | organismal development |
| 146 | A_23_P346405 | CCDC109A | mitochondrial calcium uniporter, nuclear gene encoding mitochondrial protein | 3.3 | und |
| 147 | A_33_P3338909 | ARPC5 | actin related protein 2/3 complex, subunit 5, 16kDa | 3.3 | cellular assembly and organization |
| 148 | A_24_P175176 | PHTF2 | putative homeodomain transcription factor 2, transcript variant 2 | 3.3 | und |
| 149 | A_23_P77174 | PIGB | phosphatidylinositol glycan anchor biosynthesis, class B | 3.3 | und |
| 150 | A_33_P3383331 | SLC35A3 | solute carrier family 35 (UDP-N-acetylglucosamine (UDP-GlcNAc) transporter), member A3 | 3.3 | und |
| 151 | A_23_P141394 | WIPI1 | WD repeat domain, phosphoinositide interacting 1 | 3.3 | und |
| 152 | A_23_P316612 | GLIS1 | GLIS family zinc finger 1 | 3.3 | gene expression |
| 153 | A_32_P116058 | FLJ35348 | long intergenic non-protein coding RNA 94 | 3.2 | und |
| 154 | A_19_P00316836 | XLOC_001537 |  | 3.2 | und |
| 155 | A_23_P66117 | ITFG3 | integrin alpha FG-GAP repeat containing 3 | 3.2 | und |
| 156 | A_24_P270728 | NUPR1 | nuclear protein, transcriptional regulator, 1, transcript variant 1 | 3.2 | cellular death and survival, DNA replication, recombination, and repair, cell cycle, cell growth and proliferation |
| 157 | A_23_P325924 | FAM59B | family with sequence similarity 59, member B , transcript variant 2 | 3.2 | und |
| 158 | A_33_P3219641 | CEP78 | centrosomal protein 78kDa, transcript variant 1 | 3.1 | und |
| 159 | A_23_P371682 | GPC6 | glypican 6 | 3.1 | und |
| 160 | A_23_P15285 | C16orf68 | methyltransferase like 22 | 3.1 | und |
| 161 | A_23_P1615 | FIBP | fibroblast growth factor (acidic) intracellular binding protein, transcript variant 2 | 3.1 | und |
| 162 | A_33_P3416937 | C9orf6 | chromosome 9 open reading frame 6 | 3.1 | und |
| 163 | A_33_P3264577 | DCTN1 | dynactin 1, transcript variant 1 | 3.1 | cell death and survival, embryonic development, organismal survival, cellular assembly and organization, cellular function and maintenance, cell cycle |
| 164 | A_33_P3251227 | C1GALT1 | core 1 synthase, glycoprotein-N-acetylgalactosamine 3-beta-galactosyltransferase, 1 | 3.1 | organismal development, tissue morphology, organismal survival, cell to cell signaling and interaction, cellular movement, inflamamtory response |
| 165 | A_23_P213000 | WDR1 | Homo sapiens WD repeat domain 1 (WDR1), transcript variant 1, mRNA [NM_017491] | 3.1 | organismal development, organismal survival |
| 166 | A_33_P3246007 | APOA1BP | apolipoprotein A-I binding protein | 3.1 | und |
| 167 | A_23_P42045 | ORC3L | origin recognition complex, subunit 3, transcript variant 1 | 3.1 | und |
| 168 | A_23_P501372 | SMCR7 | Smith-Magenis syndrome chromosome region, candidate 7, nuclear gene encoding mitochondrial protein, transcript variant 1 | 3.1 | und |
| 169 | A_23_P145711 | C7orf10 | chromosome 7 open reading frame 10, transcript variant 4 | 3.1 | und |
| 170 | A_23_P316150 | IFT80 | intraflagellar transport 80 homolog (Chlamydomonas), transcript variant 1 | 3.1 | developmental disorder, hereditary disorder |
| 171 | A_33_P3300158 | LOC100130078 | cDNA FLJ37340 fis, clone BRAMY2020574 | 3.1 | und |
| 172 | A_33_P3465247 | KIF3A | kinesin family member 3A | 3.1 | cellular death and survival, embryonic development, organismal development, tissue development, cellular assembly and organization, cellular function and maintenance, organ development, organismal survival |
| 173 | A_23_P251051 | NF2 | neurofibromin 2 (merlin), transcript variant 8 | 3.0 | cell death and survival, embryonic development, organismal development, tissue development, cell cycle, organismal survival |
| 174 | A_24_P319942 | SSR3 | signal sequence receptor, gamma (translocon-associated protein gamma) | 3.0 | infectious disease |
| 175 | A_24_P305764 | SMS | spermine synthase | 3.0 | und |
| 176 | A_23_P214678 | CUTA | cutA divalent cation tolerance homolog (E. coli), transcript variant 2 | 3.0 | protein synthesis |
| 177 | A_33_P3252834 | PHLDA3 | pleckstrin homology-like domain, family A, member 3 | 3.0 | cell death and survival, DNA replication, recombination, and repair |
| 178 | A_24_P219114 | SEL1L | sel-1 suppressor of lin-12-like (C. elegans), transcript variant 1 | 3.0 | cellular growth and proliferation |
| 179 | A_32_P192545 | TCEAL6 | transcription elongation factor A (SII)-like 6 | 3.0 | und |
| 180 | A_33_P3269718 | IVD | isovaleryl-CoA dehydrogenase, nuclear gene encoding mitochondrial protein, transcript variant 2 | 3.0 | hematological disorder, metabolic disease, renal and urological disease, protein synthesis, developmental disorder, hereditary disorder |
| 181 | A_33_P3361202 | C1orf96 | chromosome 1 open reading frame 96 | 3.0 | und |
| 182 | A_33_P3232339 | LOC100129119 | hypothetical LOC100129119, transcript variant 1 | 3.0 | und |
| 183 | A_32_P174572 | HTR7P | 5-hydroxytryptamine (serotonin) receptor 7 pseudogene 1 | 2.9 | und |
| 184 | A_24_P74371 | CTSA | cathepsin A (CTSA), transcript variant 1 | 2.9 | organismal development, cellular assembly and organization, cell morphology, metabolic disease, organ morphology, organismal survival |
| 185 | A_23_P53152 | C11orf17 | A kinase (PRKA) interacting protein 1, transcript variant 1 | 2.9 | und |
| 186 | A_33_P3223923 | PDIA3 | protein disulfide isomerase family A, member 3 | 2.9 | cell death and survival, cell to cell signaling and interaction, cellular growth and proliferation, infectious disease, cancer, post translational modification |
| 187 | A_33_P3315190 | MICAL2 | microtubule associated monoxygenase, calponin and LIM domain containing 2 | 2.9 | und |
| 188 | A_33_P3231367 | ATXN10 | ataxin 10, transcript variant 1 | 2.9 | und |
| 189 | A_33_P3258091 | RNPEP | arginyl aminopeptidase (aminopeptidase B) | 2.9 | und |
| 190 | A_19_P00803439 |  | lincRNA:chr16:3662649-3668149 forward strand | 2.9 | und |
| 191 | A_33_P3286481 |  |  | 2.9 | und |
| 192 | A_23_P116602 | USP35 | ubiquitin specific peptidase 35 | 2.9 | und |
| 193 | A_33_P3384452 | TFDP1 | transcription factor Dp-1, transcript variant 1 | 2.9 | cellular death and survival, embryonic development, organismal development, tissue development, cell cycle, organismal survival |
| 194 | A_23_P4885 | AP2A1 | adaptor-related protein complex 2, alpha 1 subunit, transcript variant 1 | 2.9 | infectious disease, molecular transport, protein trafficking, |
| 195 | A_32_P123629 | C18orf17 | tetratricopeptide repeat domain 39C, transcript variant 1 | 2.9 | und |
| 196 | A_24_P166663 | CDK6 | cyclin-dependent kinase 6, transcript variant 1 | 2.9 | cellular death and survival, embryonic development, organismal development, cell cycle, organ morphology, organismal survival, cellular development |
| 197 | A_23_P206532 | PHKB | phosphorylase kinase, beta, transcript variant 2 | 2.9 | metabolic disease, developmental disorder, hereditary disorder |
| 198 | A_24_P331904 | COMMD4 | COMM domain containing 4 | 2.9 | cell death and survival |
| 199 | A_33_P3325018 | C2orf30 | endoplasmic reticulum lectin 1, transcript variant 1 | 2.9 | und |
| 200 | A_23_P132669 | GLT8D1 | glycosyltransferase 8 domain containing 1, transcript variant 3 | 2.8 | und |
| 201 | A_24_P110558 | LOC492311 | IgA-inducing protein homolog (Bos taurus) | 2.8 | und |
| 202 | A_23_P83192 | PHPT1 | phosphohistidine phosphatase 1, transcript variant 3 | und | und |
| 203 | A_23_P170337 | ALDH4A1 | aldehyde dehydrogenase 4 family, member A1, nuclear gene encoding mitochondrial protein, transcript variant P5CDhL | 2.8 | hematological disease, metabolic disease, developmental disorder, hereditary disorder |
| 204 | A_24_P367329 | LOC440292 | hypothetical protein LOC440292 | 2.8 | und |
| 205 | A_23_P90612 | MCM6 | minichromosome maintenance complex component 6 | 2.8 | DNA replication, recombination, and repair, metabolic disease, developmental disorder, hereditary disorder |
| 206 | A_24_P49747 |  | high mobility group protein B3-like (LOC646993), mRNA [XM_929965] | 2.8 | und |
| 207 | A_23_P502797 | WDFY1 | WD repeat and FYVE domain containing 1 | 2.8 | und |
| 208 | A_33_P3503537 | LOC285178 | cDNA FLJ34252 fis, clone FCBBF5000061 | 2.8 | und |
| 209 | A_33_P3329557 | LOC100652869 | hypothetical protein LOC100652869 | 2.8 | und |
| 210 | A_33_P3419594 | PDE9A | cDNA FLJ90181 fis, clone MAMMA1000706 | 2.8 | und |
| 211 | A_24_P415601 | RNH1 | ribonuclease/angiogenin inhibitor 1, transcript variant 1 | 2.8 | infectious disease, cellular growth and proliferation, cancer |
| 212 | A_32_P218228 | FAM109B | family with sequence similarity 109, member B | 2.8 | und |
| 213 | A_33_P3226643 | ATG9A | ATG9 autophagy related 9 homolog A (S. cerevisiae), transcript variant 1 | 2.8 | inflammatory response |
| 214 | A_23_P213718 | UQCRQ | ubiquinol-cytochrome c reductase, complex III subunit VII, 9.5kDa, nuclear gene encoding mitochondrial protein | 2.8 | und |
| 215 | A_33_P3338968 |  |  | 2.8 | und |
| 216 | A_19_P00805955 |  | lincRNA:chr14:58847272-58852597 forward strand | 2.8 | und |
| 217 | A_33_P3310780 | CTTN | cortactin (CTTN), transcript variant 1 | 2.8 | cell death and survival, tissue development, cellular assembly, organization, function and maintenance, cell morphology, cell to cell signaling and interaction, cellular movement |
| 218 | A_24_P212072 | ANKRD32 | ankyrin repeat domain 32 | 2.8 | und |
| 219 | A_23_P95594 | NAT1 | N-acetyltransferase 1 (arylamine N-acetyltransferase), transcript variant 5 | 2.8 | organismal development, organismal survival |
| 220 | A_23_P58877 | GOPC | golgi-associated PDZ and coiled-coil motif containing , transcript variant 1 | 2.8 | infectious disease, protein synthesis, cellular compromise |
| 221 | A_23_P32903 | OCRL | oculocerebrorenal syndrome of Lowe , transcript variant a | 2.7 | metabolic disease, renal and urological disease, developmental disorder, hereditary disorder |
| 222 | A_24_P302802 | PCCB | propionyl CoA carboxylase, beta polypeptide , nuclear gene encoding mitochondrial protein, transcript variant 1 | 2.7 | hematological disease, metabolic disease, cellular movement |
| 223 | A_32_P87531 | DNAH14 | dynein, axonemal, heavy chain 14, transcript variant 2 | 2.7 | und |
| 224 | A_33_P3289218 | AAK1 | AP2 associated kinase 1 (AAK1) | 2.7 | cell death and survival, post translational modification |
| 225 | A_33_P3262814 | REEP3 | receptor accessory protein 3 | 2.7 | und |
| 226 | A_23_P54376 | STOML1 | stomatin (EPB72)-like 1 | 2.7 | und |
| 227 | A_33_P3334225 | ACACA | acetyl-CoA carboxylase alpha , transcript variant 2 | 2.7 | embryonic development, organismal development, organismal survival |
| 228 | A_33_P3210585 |  |  |  |  |
| 229 | A_23_P15348 | MPRIP | myosin phosphatase Rho interacting protein, transcript variant 1 | 2.7 | cell death and survival, cell morphology, skeletical and muscular system development |
| 230 | A_24_P244410 | C11orf51 | chromosome 11 open reading frame 51 | 2.7 | unt |
| 231 | A_23_P126803 | ARPC5 | actin related protein 2/3 complex, subunit 5, 16kDa | 2.7 | cellular assembly and organization |
| 232 | A_23_P500892 | TUB | tubby homolog (mouse), transcript variant 1 | 2.7 | cell death and survival, tissue development, cellular assembly and organization, cellular function and maintenance |
| 233 | A_23_P151746 | ISCA2 | iron-sulfur cluster assembly 2 homolog (S. cerevisiae) | 2.7 | und |
| 234 | A_33_P3305467 |  |  | 2.7 | und |
| 235 | A_33_P3392325 | CDC16 | cell division cycle 16 homolog (S. cerevisiae), transcript variant 2 | 2.7 | cell cycle, cellular growth and proliferation |
| 236 | A_23_P42080 | TMEM14A | transmembrane protein 14A | 2.7 | cell death and survival |
| 237 | A_23_P144578 | GNPDA2 | glucosamine-6-phosphate deaminase 2 | 2.7 | und |
| 238 | A_19_P00318860 | XLOC_011183 |  | 2.7 | und |
| 239 | A_33_P3275290 | GLT8D1 | glycosyltransferase 8 domain containing 1, transcript variant 3 | 2.7 | und |
| 240 | A_23_P101950 | MDH1 | malate dehydrogenase 1, NAD (soluble), transcript variant 2 | 2.7 | cell death and survival, nucleic acid metabolism, small moleccule biochemistry |
| 241 | A_33_P3292602 |  |  | 2.7 | und |
| 242 | A_23_P26954 | VAT1 | vesicle amine transport protein 1 homolog (T. californica) | 2.6 | und |
| 243 | A_19_P00324529 |  | lincRNA:chr1:98439262-98699162 reverse strand | 2.6 | und |
| 244 | A_23_P126499 | C1orf89 | REM2 and RAB-like small GTPase 1 | 2.6 | und |
| 245 | A_33_P3303136 | SERPINB6 | serpin peptidase inhibitor, clade B (ovalbumin), member 6, transcript variant 2 | 2.6 | infectious disease, neurological disease, hereditary disorder, auditory disease |
| 246 | A_33_P3221438 | C3orf21 | xyloside xylosyltransferase | 2.6 | und |
| 247 | A_33_P3410409 | LAMP2 | lysosomal-associated membrane protein 2, transcript variant C | 2.6 | cellular death and survival, organismal development, cellular assembly and organization, cell morphology, metabolic disease, organ morphology, organismal survival |
| 248 | A_33_P3416321 | SLC17A5 | solute carrier family 17 (anion/sugar transporter), member 5 | 2.6 | metabolic disease, neurological disorder, developmental disorder, hereditary disorder |
| 249 | A_33_P3393927 | MTMR11 | myotubularin related protein 11, transcript variant 2 | 2.6 | und |
| 250 | A_19_P00806158 |  |  | 2.6 | und |
| 251 | A_33_P3299872 | HINT3 | histidine triad nucleotide binding protein 3 | 2.6 | und |
| 252 | A_33_P3396527 | POLR3G | polymerase (RNA) III (DNA directed) polypeptide G (32kD) | 2.6 | gene expression, inflammatory response |
| 253 | A_33_P3276519 | GBL | MTOR associated protein, LST8 homolog (S. cerevisiae), transcript variant 1 | 2.6 | inflammatory response |
| 254 | A_33_P3209741 | KATNAL2 | katanin p60 subunit A-like 2 | 2.6 | und |
| 255 | A_23_P8522 | TMEM106B | transmembrane protein 106B , transcript variant 1 | 2.6 | und |
| 256 | A_23_P130856 | CCDC123 | centrosomal protein 89kDa (CEP89) | 2.6 | und |
| 257 | A_33_P3359157 | C1orf2 | family with sequence similarity 189, member B (FAM189B), transcript variant 1 | 2.6 | und |
| 258 | A_19_P00328934 |  | lincRNA:chr12:117562392-117569365 reverse strand | 2.6 | und |
| 259 | A_23_P19348 | CUL7 | cullin 7 (CUL7), transcript variant 2 | 2.6 | cellular death and survival, embryonic development, organismal development, tissue development, cell cycle, organismal survival |
| 260 | A_24_P382467 | SLC39A3 | solute carrier family 39 (zinc transporter), member 3, transcript variant 1 | 2.6 | und |
| 261 | A_33_P3244026 | VISA | mitochondrial antiviral signaling protein (MAVS), nuclear gene encoding mitochondrial protein, transcript variant 1 | 2.5 | und |
| 262 | A_23_P115356 | AKR7A2 | aldo-keto reductase family 7, member A2 (aflatoxin aldehyde reductase) | 2.5 | und |
| 263 | A_23_P148463 | CUL4B | cullin 4B, transcript variant 1 | 2.5 | cell death and survival, cellular development, cellular growth and proliferation, neurological disorder, developmental disorder, hereditary disorder |
| 264 | A_33_P3293391 | LOC642826 | uncharacterized LOC642826 | 2.5 | und |
| 265 | A_19_P00326865 | C6orf147 | chromosome 6 open reading frame 147 | 2.5 | und |
| 266 | A_23_P5731 | FAHD2A | fumarylacetoacetate hydrolase domain containing 2A | 2.5 | und |
| 267 | A_24_P67681 |  |  | 2.5 | und |
| 268 | A_19_P00810328 |  | lincRNA:chr16:11468274-11482224 forward strand | 2.5 | und |
| 269 | A_23_P11331 | TCEAL8 | transcription elongation factor A (SII)-like 8, transcript variant 1 | 2.5 | und |
| 270 | A_24_P93371 | COMMD4 | COMM domain containing 4 | 2.5 | cell death and survival |
| 271 | A_33_P3259620 | TTC37 | tetratricopeptide repeat domain 37 | 2.5 | und |
| 272 | A_24_P59607 | C17orf51 | chromosome 17 open reading frame 51 ( | 2.5 | und |
| 273 | A_33_P3333471 |  | Synthetic construct gateway clone IMAGE:100020926 3' read SCARA3 | 2.5 | und |
| 274 | A_19_P00801159 |  | lincRNA:chr2:9144649-9151499 forward strand | 2.5 | und |
| 275 | A_33_P3226600 | GPAA1 | glycosylphosphatidylinositol anchor attachment protein 1 homolog (yeast) | 2.5 | und |
| 276 | A_33_P3301970 | ALS2CR2 | STE20-related kinase adaptor beta (STRADB), transcript variant 1 | 2.5 | und |
| 277 | A_23_P55174 | G6PC3 | glucose 6 phosphatase, catalytic, 3 (G6PC3), transcript variant 1 | 2.5 | organismal development, organismal survival, cell to cell interaction and function, inflammatory response, |
| 278 | A_33_P3213149 | psiTPTE22 | mRNA for hypothetical protein FLJ37713 variant, clone: ah02419 | 2.5 | und |
| 279 | A_33_P3364884 | PDHB | pyruvate dehydrogenase (lipoamide) beta, nuclear gene encoding mitochondrial protein | 2.5 | und |
| 280 | A_33_P3402500 | TMEM14C | transmembrane protein 14C, transcript variant 1 | 2.5 | und |
| 281 | A_23_P56314 | UQCR | ubiquinol-cytochrome c reductase, complex III subunit X , nuclear gene encoding mitochondrial protein | 2.5 | und |
| 282 | A_23_P146644 | ANXA2 | annexin A2, transcript variant 2 | 2.4 | cell death and survival, organismal development, cellular assembly and organization, cell morphology, cellular growth and proliferation, cancer |
| 283 | A_23_P430181 | ZBTB3 | zinc finger and BTB domain containing 3 | 2.4 | und |
| 284 | A_23_P387552 | RARG | retinoic acid receptor, gamma, transcript variant 1 | 2.4 | cellular death and survival, embryonic development, organismal development, tissue development, cell cycle, organ development, organismal survival |
| 285 | A_19_P00319682 | LRRFIP1 | leucine rich repeat (in FLII) interacting protein 1 , transcript variant 1 | 2.4 | gene expression |
| 286 | A_24_P356406 | PAFAH1B1 | platelet-activating factor acetylhydrolase 1b, regulatory subunit 1 (45kDa) | 2.4 | cell death and survival, embryonic development, tissue development, cellular function and maintenance, cell cycle, organ development |
| 287 | A_24_P406301 | NDUFB2 | NADH dehydrogenase (ubiquinone) 1 beta subcomplex, 2, 8kDa (NDUFB2), nuclear gene encoding mitochondrial protein | 2.4 | und |
| 288 | A_23_P139929 | ERP29 | endoplasmic reticulum protein 29 (ERP29), transcript variant 1, | 2.4 | molecular transport, protein trafficking |
| 289 | A_23_P309361 | C1orf59 | methyltransferase homolog 1 (Arabidopsis), transcript variant 1 | 2.4 | und |
| 290 | A_23_P145777 | NDUFA4 | NADH dehydrogenase (ubiquinone) 1 alpha subcomplex, 4, 9kDa, nuclear gene encoding mitochondrial protein, | 2.4 | und |
| 291 | A_23_P254120 | FBXO9 | F-box protein 9 , transcript variant 2 | 2.4 | und |
| 292 | A_23_P106299 | SERF2 | small EDRK-rich factor 2, transcript variant 3 | 2.4 | und |
| 293 | A_23_P117082 | HEBP1 | heme binding protein 1 | 2.4 | small molecule biochemistry, molecular transport |
| 294 | A_32_P1381 | HMGN2 | non-histone chromosomal protein HMG-17 | 2.4 | cellular assembly and organization, gene expression, infectious disease, cell to cell signaling and function, |
| 295 | A_23_P108303 | NDUFA7 | NADH dehydrogenase (ubiquinone) 1 alpha subcomplex, 7, 14.5kDa, nuclear gene encoding mitochondrial protein | 2.4 | und |
| 296 | A_19_P00317814 |  | lincRNA:chr15:25324097-25327840 reverse strand | 2.4 | und |
| 297 | A_23_P27147 | ANAPC11 | anaphase promoting complex subunit 11, transcript variant 1 | 2.4 | cell cycle |
| 298 | A_24_P143138 | FGD1 | HFYVE, RhoGEF and PH domain containing 1 | 2.4 | developmental disorder, hereditary disorder |
| 299 | A_33_P3223082 | MRPL19 | mitochondrial ribosomal protein L19 (MRPL19), nuclear gene encoding mitochondrial protein | 2.4 | gene expression |
| 300 | A_32_P150856 | LOC407835 | mitogen-activated protein kinase kinase 2 pseudogene ( | 2.4 | und |
| 301 | A_33_P3404697 | PSTK | 0+A291o+A278 | 2.4 | post translational modification |
| 302 | A_24_P378987 | DHRSX | dehydrogenase/reductase (SDR family) X-linked (DHRSX), mRNA [NM_145177] | 2.4 | und |
| 303 | A_24_P74571 | CBY1 | chibby homolog 1 (Drosophila), transcript variant 2 | 2.4 | organismal development, gene expression, hematological disease |
| 304 | A_23_P418199 |  | HCG2040265, isoform CRA_acDNA FLJ50015 | 2.4 | und |
| 305 | A_23_P26945 | NAGLU | N-acetylglucosaminidase, alpha | 2.4 | cell death and survival, embryonic development, organismal development, tissue development, cellular assembly and organization, organ development, organismal survival |
| 306 | A_33_P3289561 |  | chromosome 1 open reading frame 134 | 2.4 | und |
| 307 | A_23_P397341 | PAQR4 | progestin and adipoQ receptor family member IV | 2.4 | und |
| 308 | A_23_P129695 | VASN | vasorin (VASN) | 2.4 | und |
| 309 | A_24_P36299 | GRLF1 | Rho GTPase activating protein 35 (ARHGAP35) | 2.4 | und |
| 310 | A_23_P95302 | RFC5 | replication factor C (activator 1) 5, 36.5kDa, transcript variant 2 | 2.4 | und |
| 311 | A_23_P112634 | C4orf34 | chromosome 4 open reading frame 34 ( | 2.4 | und |
| 312 | A_33_P3313889 | TOR1A | torsin family 1, member A (torsin A) | 2.4 | und |
| 313 | A_24_P328492 | SOCS5 | suppressor of cytokine signaling 5 (SOCS5), transcript variant 2 | 2.4 | cell cycle |
| 314 | A_24_P96593 | EVI5 | ecotropic viral integration site 5 | 2.4 | cell growth and proliferation |
| 315 | A_33_P3217998 | AFAR3 | aldo-keto reductase family 7-like (AKR7L), transcript variant 1 | 2.4 | und |
| 316 | A_23_P77818 | ATP5H | ATP synthase, H+ transporting, mitochondrial Fo complex, subunit d, nuclear gene encoding mitochondrial protein, transcript variant 1 | 2.4 | energy production, nucleic acid metabolism, small molecule biochemistry, DNA replication, recombination, and repair |
| 317 | A_23_P156852 | PECI | enoyl-CoA delta isomerase 2, transcript variant 2 | 2.3 | und |
| 318 | A_23_P63050 | UROD | uroporphyrinogen decarboxylas, transcript variant 1 | 2.3 | small molecule biochemistry, molecular transport |
| 319 | A_19_P00811582 |  | lincRNA:chr6:36084247-36091847 reverse strand | 2.3 | und |
| 320 | A_24_P360722 | DIP2C | DIP2 disco-interacting protein 2 homolog C (Drosophila) | 2.3 | und |
| 321 | A_23_P41872 | B4GALT7 | xylosylprotein beta 1,4-galactosyltransferase, polypeptide 7 (galactosyltransferase I) | 2.3 | cellular growth and proliferation |
| 322 | A_24_P147765 | FOXRED2 | FAD-dependent oxidoreductase domain containing 2, transcript variant 1 | 2.3 | und |
| 323 | A_33_P3380529 | PRTFDC1 | phosphoribosyl transferase domain containing 1 | 2.3 | und |
| 324 | A_19_P00320532 | IPW | Prader-Willi syndrome (non-protein coding) | 2.3 | und |
| 325 | A_33_P3782469 | PYGO1 | pygopus homolog 1 (Drosophila) | 2.3 | embryonic development, organismal development, tissue development, organ development |
| 326 | A_19_P00805168 |  | lincRNA:chr16:88805249-88810399 forward strand | 2.3 | und |
| 327 | A_19_P00800404 |  | hg18:lincRNA:chrX:114863475-114997900 forward strand | 2.3 | und |
| 328 | A_24_P151498 | PRMT2 | protein arginine methyltransferase 2 (PRMT2) | 2.3 | cellular death and survival, gene expression , cell cycle |
| 329 | A_19_P00322132 |  | lincRNA:chr4:15683502-15688229 forward strand | 2.3 | und |
| 330 | A_23_P127128 | DNAJC1 | DnaJ (Hsp40) homolog, subfamily C, member 1 | 2.3 | gene expression |
| 331 | A_23_P103099 | RBM9 | RNA binding protein, fox-1 homolog (C. elegans) 2, transcript variant 1 | 2.3 | und |
| 332 | A_23_P146512 | GOLM1 | golgi membrane protein 1, transcript variant 1 | 2.3 | und |
| 333 | A_33_P3209476 | CCM2 | cerebral cavernous malformation 2, transcript variant 2 | 2.3 | embryonic development, organismal development, tissue development, cancer, neurological disease, developmental disease, |
| 334 | A_23_P134477 | C7orf50 | chromosome 7 open reading frame 50, transcript variant 1 | 2.3 | und |
| 335 | A_32_P213624 | RTCD1 | RNA terminal phosphate cyclase domain 1, transcript variant 1 | 2.3 | und |
| 336 | A_23_P22625 | SLC9A6 | solute carrier family 9 (sodium/hydrogen exchanger), member 6, transcript variant 1 | 2.3 | infectious disease, neurological disease, hereditary disorder, auditory disease |
| 337 | A_33_P3217834 | SMARCAL1 | SWI/SNF related, matrix associated, actin dependent regulator of chromatin, subfamily a-like 1, transcript variant 1 | 2.3 | organismal development, energy production, nucleic acid metabolism, DNA replication, recombination, and repair, organismal survival, cellular growth and proliferation |
| 338 | A_23_P303260 | STX7 | syntaxin 7 (STX7) | 2.3 | molecular transport, protein trafficking |
| 339 | A_23_P13183 | EXT2 | exostosin 2 (EXT2), transcript variant 1 | 2.3 | cell death and survival, embryonic development, organismal development, tissue development, cellular compromise |
| 340 | A_33_P3429576 | AP3S1 | adaptor-related protein complex 3, sigma 1 subunit | 2.3 | molecular transport, cell mediated immunoresponse |
| 341 | A_23_P134827 | ASH2L | ash2 (absent, small, or homeotic)-like (Drosophila), transcript variant 1 | 2.3 | gene expression, DNA replication, recombination, and repair, cell growth and proliferation |
| 342 | A_19_P00812044 | MAVS | mitochondrial antiviral signaling protein, nuclear gene encoding mitochondrial protein, transcript variant 1 | 2.3 | cellular death and survival, embryonic development, organismal development, organismal survival, cellular growth and proliferation, inflammatory response |
| 343 | A_23_P16214 | FBXW9 | F-box and WD repeat domain containing 9 | 2.3 | und |
| 344 | A_23_P47377 | HSD17B12 | hydroxysteroid (17-beta) dehydrogenase 12 | 2.3 | und |
| 345 | A_19_P00319882 | LOC100130890 | uncharacterized protein LOC100130890 | 2.3 | und |
| 346 | A_24_P225961 | DAG1 | dystroglycan 1 (dystrophin-associated glycoprotein 1), transcript variant 2 | 2.2 | cell death and survival, embryonic development, organismal development, tissue development, cellular assembly and organization, tissue morphology, organismal injury and development |
| 347 | A_23_P98092 | OAT | ornithine aminotransferase (OAT), nuclear gene encoding mitochondrial protein | 2.2 | embryonic development, oganismal development, tissue development, nervous system and function, tissue and organ morphology, amino acid metabolism |
| 348 | A_23_P137073 | ZMYM3 | zinc finger, MYM-type 3 (ZMYM3), transcript variant 2 | 2.2 | und |
| 349 | A_32_P71943 | RIPK5 | dual serine/threonine and tyrosine protein kinase, transcript variant 1 | 2.2 | und |
| 350 | A_33_P3345309 | LOC646960 | protease, serine, 56 (PRSS56) | 2.2 | und |
| 351 | A_23_P208674 | C19orf63 | chromosome 19 open reading frame 63 , transcript variant HSS1 | 2.2 | und |
| 352 | A_32_P203515 | SPECC1L | sperm antigen with calponin homology and coiled-coil domains 1-like, transcript variant 1 | 2.2 | und |
| 353 | A_32_P61857 | KIAA1468 | KIAA1468 (KIAA1468) | 2.2 | und |
| 354 | A_23_P115683 | HPS6 | Hermansky-Pudlak syndrome 6 | 2.2 | metabolic disease, dermatological diseases and conditions, organismal injury and abnormalities, developmental disorders, hereditary disorder, ophthalmic disease |
| 355 | A_23_P60225 | GRHPR | glyoxylate reductase/hydroxypyruvate reductase | 2.2 | metabolic disease, renal and urological disease, developmental disorder, hereditary disorder |
| 356 | A_23_P68072 | WDR54 | WD repeat domain 54 | 2.2 | und |
| 357 | A_23_P359904 | PDDC1 | Parkinson disease 7 domain containing 1 | 2.2 | und |
| 358 | A_33_P3223472 | AMZ2 | archaelysin family metallopeptidase 2 , transcript variant 1 | 2.2 | und |
| 359 | A_23_P17512 | DTD1 | D-tyrosyl-tRNA deacylase 1 homolog (S. cerevisiae), nuclear gene encoding mitochondrial protein | 2.2 | cell cycle |
| 360 | A_23_P373054 | C3orf23 | chromosome 3 open reading frame 23, transcript variant 1 | 2.2 | und |
| 361 | A_24_P398940 | CASC4 | cancer susceptibility candidate 4, transcript variant 1 | 2.2 | und |
| 362 | A_33_P3268224 | SPATA5 | spermatogenesis associated 5 | 2.2 | und |
| 363 | A_33_P3232478 | CACNA2D1 | calcium channel, voltage-dependent, alpha 2/delta subunit 1 | 2.2 | infectious disease, neurological disease |
| 364 | A_19_P00327463 |  | lincRNA:chr6:106858857-106926507 reverse strand | 2.2 | und |
| 365 | A_23_P107073 | RPA1 | replication protein A1, 70kDa | 2.2 | cellular growth and proliferation, cellular assembly and organization, cellular function and maintenance, gene expression, DNA replication, recombination, and repair, cell cycle |
| 366 | A_23_P380998 | R3HDM1 | R3H domain containing 1 | 2.2 | und |
| 367 | A_23_P114221 | RBBP7 | retinoblastoma binding protein 7 (RBBP7), transcript variant 2 | 2.2 | gene expression, cellular growth and proliferation |
| 368 | A_33_P3286254 | AP3S1 | adaptor-related protein complex 3, sigma 1 subunit | 2.2 | molecular transport, protein trafficking |
| 369 | A_33_P3275968 | SEL1L | sel-1 suppressor of lin-12-like (C. elegans) (SEL1L), transcript variant 1 | 2.2 | cellular growth and proliferation |
| 370 | A_23_P77437 | PRMT7 | protein arginine methyltransferase 7, transcript variant 1 | 2.2 | gene expression, post translational modification |
| 371 | A_23_P56680 | ATP5G3 | ATP synthase, H+ transporting, mitochondrial Fo complex, subunit C3 (subunit 9), nuclear gene encoding mitochondrial protein, transcript variant 3 | 2.2 | und |
| 372 | A_23_P143414 | C20orf52 | reactive oxygen species modulator 1 (ROMO1), nuclear gene encoding mitochondrial protein | 2.2 | und |
| 373 | A_19_P00328867 |  | lincRNA:chr1:201555452-201589477 reverse strand | 2.2 | und |
| 374 | A_23_P371145 | ADPRHL1 | ADP-ribosylhydrolase like 1 (ADPRHL1), transcript variant 1 | 2.2 | und |
| 375 | A_33_P3530314 | C5orf42 | chromosome 5 open reading frame 42 | 2.1 | und |
| 376 | A_24_P392958 | TBC1D20 | TBC1 domain family, member 20 | 2.1 | und |
| 377 | A_23_P361049 | MYO1B | myosin IB (MYO1B), transcript variant 2 | 2.1 | und |
| 378 | A_32_P222383 | HMGN2 | high mobility group nucleosomal binding domain 2 | 2.1 | cellular assembly and organization, gene expression, infectious disease, cell to cell signaling and interaction |
| 379 | A_23_P118536 | SLFN12 | schlafen family member 12 ( | 2.1 | infectious disease, cellular growth and proliferation |
| 380 | A_23_P76761 | VRK1 | vaccinia related kinase 1 | 2.1 | gene expression, post translational modification |
| 381 | A_33_P3214874 | KPNA6 | karyopherin alpha 6 (importin alpha 7) | 2.1 | und |
| 382 | A_33_P3357620 | C16orf53 | chromosome 16 open reading frame 53 | 2.1 | und |
| 383 | A_33_P3399291 | GUK1 | guanylate kinase 1, transcript variant 5 | 2.1 | energy production, nucleic acid metabolism, small molecule biochemistry |
| 384 | A_23_P426140 | ZYG11B | zyg-11 homolog B (C. elegans) | 2.1 | und |
| 385 | A_23_P133596 | RNASEN | drosha, ribonuclease type III, transcript variant 1 | 2.1 | und |
| 386 | A_32_P37360 | LACE1 | lactation elevated 1 | 2.1 | und |
| 387 | A_24_P108779 | KCTD17 | potassium channel tetramerisation domain containing 17 | 2.1 | und |
| 388 | A_33_P3377060 | SSR1 | signal sequence receptor, alpha | 2.1 | infectious disease , cellular growth and proliferation |
| 389 | A_33_P3212782 | CALM2 | calmodulin 2 (phosphorylase kinase, delta) | 2.1 | und |
| 390 | A_24_P48162 | MPG | N-methylpurine-DNA glycosylase (MPG), transcript variant 1 | 2.1 | cellular death and survival, organismal development, cellular assembly and organization, energy production, cell cycle, organismal survival |
| 391 | A_32_P42925 | MRLC2 | myosin, light chain 12B, regulatory (MYL12B), transcript variant 2 | 2.1 | und |
| 392 | A_23_P214739 | FBXL4 | F-box and leucine-rich repeat protein 4 | 2.1 | und |
| 393 | A_24_P107336 | VPS26B | vacuolar protein sorting 26 homolog B (S. pombe) | 2.1 | und |
| 394 | A_33_P3429575 | LOC643454 | adaptor-related protein complex 3, sigma 1 subunit pseudogene, mRNA | 2.1 | und |
| 395 | A_23_P215253 |  | polymerase (RNA) II (DNA directed) polypeptide J3 | 2.1 | und |
| 396 | A_23_P213661 | HISPPD1 | diphosphoinositol pentakisphosphate kinase 2 | 2.1 | und |
| 397 | A_24_P120346 | NIT2 | nitrilase family, member 2 | 2.1 | und |
| 398 | A_32_P447001 | FLJ27352 | uncharacterized LOC145788 | 2.1 | und |
| 399 | A_23_P42649 | POLR2J | polymerase (RNA) II (DNA directed) polypeptide J, 13.3kDa | 2.1 | gene expression, infectious disease, Infectious disease , cellular growth and proliferation, post translational modification |
| 400 | A_24_P23995 | RNF187 | ring finger protein 187 | 2.1 | cellular assembly and organization, cellular growth and proliferation, post translational modification |
| 401 | A_23_P390704 | STK38 | serine/threonine kinase 38 ( | 2.1 | cellular death and survival, cell cycle, cellular growth and proliferation, post translational modification |
| 402 | A_33_P3407424 | CDC42EP1 | CDC42 effector protein (Rho GTPase binding) 1 | 2.1 | und |
| 403 | A_24_P202717 | PLEKHJ1 | pleckstrin homology domain containing, family J member 1 | 2.1 | und |
| 404 | A_23_P56249 | TBCB | tubulin folding cofactor B | 2.1 | und |
| 405 | A_23_P168669 | CROT | carnitine O-octanoyltransferase (CROT) | 2.1 | small molecule biochemistry, molecular transport, lipid metabolism |
| 406 | A_23_P137434 | RNF11 | ring finger protein 11 | 2.1 | gene expression |
| 407 | A_19_P00328165 |  | lincRNA:chr12:123614572-123628947 reverse strand | 2.1 | und |
| 408 | A_33_P3233666 | RPP30 | ribonuclease P/MRP 30kDa subunit, transcript variant 1 | 2.1 | und |
| 409 | A_24_P18917 | LMF1 | lipase maturation factor 1 (LMF1), transcript variant 1 | 2.1 | metabolic disorder, hereditary disease |
| 410 | A_33_P3221788 | RAB22A | RAB22A, member RAS oncogene family | 2.1 | und |
| 411 | A_23_P326170 | CALM2 | calmodulin 2 (phosphorylase kinase, delta) | 2.1 | und |
| 412 | A_32_P23795 |  |  | 2.1 | und |
| 413 | A_33_P3357918 | PGLS | 6-phosphogluconolactonase | 2.0 | nucleic acid metabolism, small molecule biochemistry, carbohydrate metabolism |
| 414 | A_24_P919452 |  |  | 2.0 | und |
| 415 | A_23_P47565 | LDHA | lactate dehydrogenase A, transcript variant 1 | 2.0 | cell death and survival, cellular development, cellular function and maintenance, cancer, connective tissue function and development |
| 416 | A_23_P19852 | IQCE | IQ motif containing E, transcript variant 1 | 2.0 | und |
| 417 | A_33_P3310981 | ZNF215 | zinc finger protein 215 | 2.0 | und |
| 418 | A_19_P00316263 |  | lincRNA:chr8:143279733-143281816 forward strand | 2.0 | und |
| 419 | A_24_P216253 | DLGAP4 | discs, large (Drosophila) homolog-associated protein 4, transcript variant 1 | 2.0 | infectious disease |
| 420 | A_23_P63281 | GLTPD1 | glycolipid transfer protein domain containing 1 | 2.0 | und |
| 421 | A_23_P59358 | C6orf182 | centrosomal protein 57kDa-like 1, transcript variant 2 | 2.0 | und |
| 422 | A_19_P00813410 |  | lincRNA:chr3:9428550-9436575 forward strand | 2.0 | und |
| 423 | A_33_P3821660 | C9orf86 | chromosome 9 open reading frame 86, transcript variant 4 | 2.0 | und |
| 424 | A_33_P3231653 | GOLM1 | golgi membrane protein 1, transcript variant 1 | 2.0 | und |
| 425 | A_33_P3259902 |  | high mobility group nucleosomal binding domain 2 pseudogene 28 | 2.0 | und |
| 426 | A_19_P00804950 | P39188 | ALU1_HUMAN (P39188) Alu subfamily J sequence contamination warning entry, partial (13%) | 2.0 | und |
| 427 | A_33_P3210168 | PIN1 | peptidylprolyl cis/trans isomerase, NIMA-interacting 1, transcript variant 1 | 2.0 | cell death and survival, organismal development, tissue development, cellular assembly and organization, cellular function and maintenance, DNA replication, recombination, and repair, cell cycle |
| 428 | A_23_P14948 | MBTPS1 | membrane-bound transcription factor peptidase, site 1 | 2.0 | cell death and survival, organismal development, organismal injury and abnormalities, organismal survival, skeletical and muscular disorders, developmental disorders and hereditary disorders |
| 429 | A_23_P94660 | TBC1D13 | TBC1 domain family, member 13 | 2.0 | und |
| 430 | A_23_P214211 | RARS2 | arginyl-tRNA synthetase 2, mitochondrial, nuclear gene encoding mitochondrial protein | 2.0 | neurological disease, developmental disorder |
| 431 | A_23_P259054 | SNX14 | sorting nexin 14, transcript variant 1 | 2.0 | cellular growth and proliferation, |
| 432 | A_23_P132405 | ACAD9 | acyl-CoA dehydrogenase family, member 9, nuclear gene encoding mitochondrial protein, transcript variant 1 | 2.0 | hereditary disease |
| 433 | A_23_P5389 | DPY30 | dpy-30 homolog (C. elegans) | 2.0 | und |
| 434 | A_24_P19810 | PPCS | phosphopantothenoylcysteine synthetase, transcript variant 1 | 2.0 | und |
