# Supplementary material for: Awakened by Cellular Stress: Isolation and Characterization of a Novel Population of Pluripotent Stem Cells Derived from Human Adipose Tissue
Source: PLoS One. 2013 Jun 5;8(6):e64752. doi: 10.1371/journal.pone.0064752 (PMC3673968; doi:10.1371/journal.pone.0064752)
Supplement: Table S3 — Genes expressed by Muse-AT that are not expressed by ASCs. (DOC) [file pone.0064752.s003.doc]

**Supplemental Data: Table 3**

**Genes expressed by Muse-AT that are not expressed by ASCs**

| no. | Probe Name | Gene Symbol | Gene Name | P val. | Function |
| --- | --- | --- | --- | --- | --- |
| 1 | A_24_P237036 | TNFSF14 | tumor necrosis factor (ligand) superfamily, member 14 | 0.0002 | T-cell proliferation |
| 2 | A_32_P217750 | IL3RA | interleukin 3 receptor, alpha | 0.0007 | Lymphoid/myeloid differentiation |
| 3 | A_33_P3267799 | LILRB4 | leukocyte IgG-like receptor, subfam B, member 4 | 0.0013 | Immune regulation |
| 4 | A_33_P3222424 | CSF3 | colony stimulating factor 3 | 0.0013 | WBC production/ survival |
| 5 | A_23_P501754 | CSF3 | colony stimulating factor 3 | 0.0019 | WBC production/ survival |
| 6 | A_24_P28722 | RSAD2 | radical S-adenosyl methionine domain containing 2 | 0.0019 | Antiviral defense |
| 7 | A_23_P83838 | CA8 | carbonic anhydrase VIII | 0.0020 | Neurologic function |
| 8 | A_23_P372946 | TM4SF19 | transmembrane 4 L six family member 19 | 0.0024 | unknown |
| 9 | A_33_P3356462 | FAM148A | C2 calcium-dependent domain containing 4A | 0.0027 | Acute inflammation |
| 10 | A_23_P128974 | BATF | basic leucine zipper transcription factor | 0.0031 | T_h_17 induction |
| 11 | A_23_P253317 | GPR171 | G protein-coupled receptor 171 | 0.0033 | Stem Cell differentiation |
| 12 | A_23_P431179 | HIST1H4A | histone cluster 1, H4a | 0.0033 | Histone function |
| 13 | A_33_P3489646 | SPI1 | spleen focus forming virus (SFFV) proviral integration oncogene | 0.0034 | Bcell/φ differentiation |
| 14 | A_23_P90626 | PSCDBP | cytohesin 1 interacting protein | 0.0037 | Lymphocyte adhesion |
| 15 | A_23_P150768 | SLCO2B1 | solute carrier organic anion transporter family, member 2B1 | 0.0043 | Statin transporter |
| 16 | A_33_P3316539 | SLC7A2 | solute carrier family 7, member 2 | 0.0048 | φ activation |
| 17 | A_23_P203173 | IL10RA | interleukin 10 receptor, alpha | 0.0048 | Immunosuppressive |
| 18 | A_24_P353638 | SLAMF7 | SLAM family member 7 | 0.0050 | NK activation |
| 19 | A_23_P200138 | SLAMF8 | SLAM family member 8 | 0.0052 | Lymphocyte activation |
| 20 | A_33_P3550894 | GATA2 | GATA binding protein 2 | 0.0053 | Stem cell reg |
| 21 | A_23_P152838 | CCL5 | chemokine (C-C motif) ligand 5 | 0.0054 | chemokine |
| 22 | A_23_P161076 | CD2 | CD2 molecule | 0.0057 | co-stimulator |
| 23 | A_33_P3274562 | SLC19A3 | solute carrier family 19, member 3 | 0.0058 | transporter |
| 24 | A_33_P3277514 | BTK | Bruton agammaglobulinemia tyrosine kinase | 0.0061 | B-cell development |
| 25 | A_33_P3243230 | IL8 | interleukin 8 | 0.0062 | chemotactic factor |
| 26 | A_23_P92499 | TLR2 | toll-like receptor 2 | 0.0068 | immune regulation |
| 27 | A_33_P3252068 | JHDM1D | jumonji C domain containing histone demethylase 1 homolog D | 0.0068 | histone demethylase |
| 28 | A_24_P148717 | CCR1 | chemokine (C-C motif) receptor 1 | 0.0073 | immune regulation |
| 29 | A_24_P11506 | KYNU | kynureninase, transcript variant 2 | 0.0074 | Metabolism |
| 30 | A_24_P242646 | CTSS | cathepsin S, transcript variant 1 | 0.0076 | Ag Presentation |
| 31 | A_23_P338479 | CD274 | CD274 molecule | 0.0076 | T cell costimulation |
| 32 | A_24_P274831 | GIMAP7 | GTPase, IMAP family member 7 | 0.0089 | T cell survival |
| 33 | A_33_P3296181 | CCL3L3 | chemokine (C-C motif) ligand 3-like 3 | 0.0093 | chemotactic factor |
| 34 | A_24_P788878 | FAM148B | C2 calcium-dependent domain containing 4B | 0.0103 | Acute inflammation |
| 35 | A_23_P145096 | PLA2G7 | phospholipase A2, group VII | 0.0109 | foam cell formation |
| 36 | A_33_P3318414 | HMHA1 | histocompatibility (minor) HA-1 | 0.0110 | immune recognition |
| 37 | A_24_P153840 | FGD3 | FYVE, RhoGEF and PH domain containing 3 | 0.0118 | Actin remodeling |
| 38 | A_23_P38959 | VAV1 | vav 1 guanine nucleotide exchange factor | 0.0119 | B/T cell development |
| 39 | A_23_P216340 | SLA | Src-like-adaptor, transcript variant 1 | 0.0125 | Tcell, DC development |
| 40 | A_23_P72096 | IL1A | interleukin 1, alpha | 0.0131 | Inflamm cytokine |
| 41 | A_33_P3248265 | LTB | lymphotoxin beta | 0.0136 | Inflam, Neural SC differentiation |
| 42 | A_23_P218626 | NEU4 | sialidase 4, transcript variant 1 | 0.0136 | Sialidase |
| 43 | A_23_P37702 | TPSAB1 | tryptase alpha/beta 1 | 0.0143 | Mast cell marker |
| 44 | A_32_P163247 | CD8A | CD8a molecule | 0.0146 | co-receptor |
| 45 | A_33_P3247042 | FPR3 | formyl peptide receptor 3 | 0.0152 | PMN activation |
| 46 | A_23_P18017 | CPA3 | carboxypeptidase A3 | 0.0154 | Mast cell formation |
| 47 | A_23_P82775 | SOX17 | SRY (sex determining region Y)-box 17 | 0.0157 | Cell fate |
| 48 | A_23_P343398 | CCR7 | chemokine (C-C motif) receptor 7 | 0.0159 | Chemokine |
| 49 | A_23_P323761 | TRAF3IP3 | TRAF3 interacting protein 3 | 0.0167 | Immunity |
| 50 | A_23_P23279 | RCSD1 | RCSD domain containing 1 | 0.0168 | Actin remodeling |
| 51 | A_33_P3372004 | IGSF6 | immunoglobulin superfamily, member 6 | 0.0181 | co-receptor |
| 52 | A_24_P340128 | P2RY8 | purinergic receptor P2Y, G-protein coupled, 8 | 0.0186 | G-protein signaling |
| 53 | A_23_P376488 | TNF | tumor necrosis factor | 0.0188 | Inflamm cytokine |
| 54 | A_23_P30547 | LCP2 | lymphocyte cytosolic protein 2 | 0.0189 | Immunity |
| 55 | A_23_P100730 | SKAP1 | src kinase associated phosphoprotein 1 | 0.0190 | T-cell function |
| 56 | A_24_P206604 | PFKFB3 | 6-phosphofructo-2-kinase/fructose-2,6-biphosphatase 3 (PFKFB3) | 0.0194 | Metabolism |
| 57 | A_33_P3285540 | CLDN5 | claudin 5 | 0.0199 | Tight Junctions |
| 58 | A_23_P85800 | CD52 | CD52 molecule | 0.0205 | Surface Marker |
| 59 | A_33_P3354607 | CCL4 | chemokine (C-C motif) ligand 4 | 0.0209 | Chemokine |
| 60 | A_24_P209455 | GIMAP4 | GTPase, IMAP family member 4 | 0.0209 | Immunity |
| 61 | A_33_P3319905 | TREM1 | triggering receptor expressed on myeloid cells 1 | 0.0210 | Immune Response |
| 62 | A_23_P68487 | BMP7 | bone morphogenetic protein 7 | 0.0212 | Differentiation |
| 63 | A_33_P3382324 | TPSD1 | tryptase delta 1 | 0.0215 | Immune Reactions |
| 64 | A_23_P214627 | AIF1 | allograft inflammatory factor 1, transcript variant 2 | 0.0221 | Immunity |
| 65 | A_33_P3287158 | NLGN4X | neuroligin 4, X-linked, transcript variant 1 | 0.0230 | neurologic function |
| 66 | A_33_P3293049 | HLA-DQA1 | major histocompatibility complex, class II, DQ alpha 1 | 0.0235 | immune recognition |
| 67 | A_23_P25155 | GPR84 | G protein-coupled receptor 84 | 0.0237 | FA metab and immunity |
| 68 | A_24_P347378 | ALOX5AP | arachidonate 5-lipoxygenase-activating protein | 0.0248 | Leukotriene synthesis |
| 69 | A_23_P140384 | CTSG | cathepsin G | 0.0252 | Immunity (complement) |
| 70 | A_23_P29005 | SAMSN1 | SAM domain, SH3 domain and nuclear localization signals 1 | 0.0253 | HSC regulator |
| 71 | A_33_P3842556 | IKZF1 | IKAROS family zinc finger 1, transcript variant 1 | 0.0254 | HSC differentiation |
| 72 | A_23_P52761 | MMP7 | matrix metallopeptidase 7 | 0.0259 | ECM remodeling |
| 73 | A_24_P192914 | AMICA1 | adhesion molecule, interacts with CXADR antigen 1, variant 2 | 0.0260 | Immunity |
| 74 | A_24_P365767 | CYBB | cytochrome b-245, beta polypeptide | 0.0260 | Immunity |
| 75 | A_23_P113351 | SPARCL1 | SPARC-like 1, transcript variant 2 | 0.0263 | Stem cell reg |
| 76 | A_23_P218369 | CCL14 | chemokine (C-C motif) ligand 14, transcript variant 3 | 0.0267 | Stem cell reg |
| 77 | A_23_P117662 | HDC | histidine decarboxylase | 0.0269 | Metabolism |
| 78 | A_33_P3211432 | NCF1 | neutrophil cytosolic factor 1 | 0.0275 | Immunity |
| 79 | A_23_P434809 | S100A8 | S100 calcium binding protein A8 | 0.0276 | Immunity |
| 80 | A_33_P3231414 | LILRB1 | leukocyte IgG-like receptor, subfam B | 0.0278 | immune recognition |
| 81 | A_23_P312132 | ITGAX | integrin, alpha X | 0.0279 | Immunity |
| 82 | A_23_P69310 | CCRL2 | chemokine (C-C motif) receptor-like 2 | 0.0282 | Th2 induction |
| 83 | A_33_P3281273 | S1PR4 | sphingosine-1-phosphate receptor 4 | 0.0283 | lymphocyte migration |
| 84 | A_23_P152002 | BCL2A1 | BCL2-related protein A1, transcript variant 1 | 0.0289 | HSC promotion |
| 85 | A_23_P81898 | UBD | ubiquitin D | 0.0295 | Ubiquitination |
| 86 | A_23_P167920 | DLL1 | delta-like 1 | 0.0296 | Stem cell reg |
| 87 | A_24_P305345 | CD209 | CD209 molecule, transcript variant 1 | 0.0298 | T-cell activation |
| 88 | A_23_P98410 | CD3G | CD3g molecule, gamma | 0.0299 | T-cell marker |
| 89 | A_33_P3352098 | MS4A7 | membrane-spanning 4-domains, subfamily A, member 7 | 0.0305 | HSC regulator |
| 90 | A_33_P3251876 | IL18R1 | interleukin 18 receptor 1 | 0.0307 | Inflammation |
| 91 | A_23_P106362 | AQP9 | aquaporin 9 | 0.0309 | transporter |
| 92 | A_33_P3313622 | MIRHG1 | miR-17-92 cluster host gene | 0.0310 | microRNA |
| 93 | A_33_P3327444 | BACH1 | BTB and CNC homology 1, basic leucine zipper transcription factor 1 | 0.0318 | Cell regulation |
| 94 | A_23_P153562 | C5AR1 | complement component 5a receptor 1 | 0.0324 | Immunity (complement) |
| 95 | A_23_P97112 | SELE | selectin E | 0.0330 | Inflammation |
| 96 | A_23_P166408 | OSM | oncostatin M | 0.0377 | Growth regulator |
| 97 | A_23_P133408 | CSF2 | colony stimulating factor 2 | 0.0379 | growth factor (SCs) |
| 98 | A_33_P3376821 | GZMA | granzyme A | 0.0478 | Immunity |
| 99 | A_33_P3405213 | PECAM1 | platelet/endothelial cell adhesion molecule | 0.0491 | Adipose SC marker |
| 100 | A_23_P99275 | KLRB1 | killer cell lectin-like receptor subfamily B, member 1 | 0.0493 | NK function |
