# Supplementary material for: Awakened by Cellular Stress: Isolation and Characterization of a Novel Population of Pluripotent Stem Cells Derived from Human Adipose Tissue
Source: PLoS One. 2013 Jun 5;8(6):e64752. doi: 10.1371/journal.pone.0064752 (PMC3673968; doi:10.1371/journal.pone.0064752)
Supplement: Table S4 — Cluster of Differentiation (CD) genes expressed by Muse-AT that are not expressed by ASCs. (DOC) [file pone.0064752.s004.doc]

**Supplemental Data: Table 4**

**Cluster of Differentiation *(*CD*)* genes expressed by Muse-AT that are not expressed by ASCs**

| no. | ProbeName | Gene Symbol | Gene Name | P val | Function |
| --- | --- | --- | --- | --- | --- |
| 1 | A_23_P161076 | CD2 | CD2 molecule | 0.0057 | co-stimulator |
| 2 | A_23_P98410 | CD3G | CD3g molecule, gamma | 0.0299 | T-cell marker |
| 3 | A_32_P163247 | CD8A | CD8a molecule, transcript variant 1 | 0.0146 | co-receptor |
| 4 | A_23_P312132 | ITGAX | integrin, alpha X | 0.0279 | Immunity |
| 5 | A_33_P3352098 | MS4A7 | membrane-spanning 4-domains, subfamily A, member 7 | 0.0306 | HSC regulator |
| 6 | A_23_P252471 | PECAM1 | platelet/endothelial cell adhesion molecule | 0.0368 | Adipose SC marker |
| 7 | A_33_P3403576 | FCGR2A | Fc fragment of IgG, low affinity IIa, receptor | 0.0503 | Opsonization |
| 8 | A_33_P3364811 | PTPRC | protein tyrosine phosphatase, receptor type, C | 0.0346 | T-cell activation |
| 9 | A_23_P85800 | CD52 | CD52 molecule | 0.0205 | Surface Marker |
| 10 | A_23_P74547 | CD53 | CD53 molecule (CD53), transcript variant 1 | 0.0428 | Immunity |
| 11 | A_23_P97112 | SELE | selectin E (SELE) | 0.0330 | Inflammation |
| 12 | A_32_P217750 | IL3RA | interleukin 3 receptor, alpha | 0.0007 | Lymphoid/myeloid differentiation |
| 13 | A_23_P99275 | KLRB1 | killer cell lectin-like receptor subfamily B, member 1 | 0.0493 | NK function |
| 14 | A_24_P148717 | CCR1 | chemokine (C-C motif) receptor 1 | 0.0073 | immune regulation |
| 15 | A_23_P343398 | CCR7 | chemokine (C-C motif) receptor 7 | 0.0159 | Chemokine |
| 16 | A_24_P305345 | CD209 | CD209 molecule | 0.0298 | T-cell activation |
| 17 | A_23_P203173 | IL10RA | interleukin 10 receptor, alpha | 0.0048 | Immunosuppressive |
| 18 | A_24_P237036 | TNFSF14 | tumor necrosis factor (ligand) superfamily, member 14 | 0.0002 | T-cell proliferation |
| 19 | A_23_P338479 | CD274 | CD274 molecule | 0.0076 | T cell costimulation |
| 20 | A_23_P92499 | TLR2 | toll-like receptor 2 | 0.0068 | immune regulation |
| 21 | A_23_P200138 | SLAMF8 | SLAM family member 8 | 0.0052 | Lymphocyte activation |
| 22 | A_33_P3319905 | TREM1 | triggering receptor expressed on myeloid cells 1 | 0.0210 | Immune Response |
