# Supplementary material for: Awakened by Cellular Stress: Isolation and Characterization of a Novel Population of Pluripotent Stem Cells Derived from Human Adipose Tissue
Source: PLoS One. 2013 Jun 5;8(6):e64752. doi: 10.1371/journal.pone.0064752 (PMC3673968; doi:10.1371/journal.pone.0064752)
Supplement: Table S5 — Genes Expressed by ASCs that are not expressed in Muse-AT cells. (DOC) [file pone.0064752.s005.doc]

**Supplemental Data: Table 5**

**Genes Expressed by ASCs that are not expressed in Muse-AT cells**

| no. | Probe Name | Gene Symbol | Gene Name | P val | Function |
| --- | --- | --- | --- | --- | --- |
| 1 | A_24_P323598 | ESCO2 | establishment of cohesion 1 homolog 2 | 0.0007 | Mitosis |
| 2 | A_23_P256956 | KIF20A | kinesin family member 20A | 0.0009 | Mitosis |
| 3 | A_24_P96780 | CENPF | centromere protein F | 0.0023 | Mitosis |
| 4 | A_24_P319613 | NEK2 | NIMA (never in mitosis gene a)-related kinase 2 | 0.0029 | Mitosis |
| 5 | A_23_P388168 | RAB3B | RAB3B, member RAS oncogene family | 0.0031 | Protein transport |
| 6 | A_33_P3288159 | ASPM | asp (abnormal spindle) homolog, microcephaly associated | 0.0037 | Mitosis |
| 7 | A_24_P322354 | SKA1 | spindle and kinetochore associated complex subunit 1 | 0.0039 | Mitosis |
| 8 | A_32_P150891 | DIAPH3 | diaphanous homolog 3 | 0.0048 | Actin |
| 9 | A_24_P943894 | SCUBE3 | signal peptide, CUB domain, EGF-like 3 | 0.0058 | Ca^2+^-binding |
| 10 | A_23_P212800 | FGF5 | fibroblast growth factor 5 | 0.0068 | Cell Cycle |
| 11 | A_23_P162719 | DIAPH3 | diaphanous homolog 3, transcript variant 2 | 0.0068 | Actin Remodeling |
| 12 | A_23_P130182 | AURKB | aurora kinase B | 0.0079 | Mitosis |
| 13 | A_23_P88331 | DLGAP5 | discs, large homolog-associated protein 5 | 0.0079 | Cell Cycle |
| 14 | A_23_P17593 | CDH4 | cadherin 4, type 1, R-cadherin, transcript variant 1 | 0.0084 | Development |
| 15 | A_33_P3294917 | PRUNE2 | prune homolog 2 | 0.0100 | Cell survival |
| 16 | A_23_P345707 | C15orf42 | Chromosome 15 open reading frame 42 | 0.0100 | Cell Cycle TOPBP1-interacting checkpoint and replication regulator. |
| 17 | A_23_P51587 | RGS7 | regulator of G-protein signaling 7 (RGS7) | 0.0103 | Signaling (NT) |
| 18 | A_23_P29723 | SGOL1 | shugoshin-like 1 (S. pombe) (SGOL1), transcript variant A2 | 0.0112 | Mitosis |
| 19 | A_33_P3271930 | PYCR1 | pyrroline-5-carboxylate reductase 1, transcript variant 2 | 0.0113 | Metabolism |
| 20 | A_24_P419132 | CENPI | centromere protein I | 0.0117 | Mitosis |
| 21 | A_24_P225970 | SGOL1 | shugoshin-like 1, transcript variant A1 | 0.0128 | Mitosis |
| 22 | A_24_P296254 | ARHGAP11A | Rho GTPase activating protein 11A | 0.0145 | Activated in iPS |
| 23 | A_24_P346855 | MKI67 | antigen identified by monoclonal antibody Ki-67 | 0.0157 | Proliferation |
| 24 | A_23_P333228 | C3HC4 | membrane-associated ring finger 4 | 0.0159 | Ubiquitination |
| 25 | A_33_P3366221 | NTNG1 | netrin G1, transcript variant 3 | 0.0159 | Migration |
| 26 | A_33_P3253596 | KIF4A | kinesin family member 4A | 0.0159 | Mitosis |
| 27 | A_19_P00318057 |  | lincRNA:chr4:15670037-15683240 reverse strand | 0.0164 | unknown |
| 28 | A_33_P3221898 |  | keratin 17 pseudogene | 0.0171 | unknown |
| 29 | A_33_P3326210 | ESCO2 | establishment of cohesion 1 homolog 2 | 0.0171 | Mitosis |
| 30 | A_33_P3221313 | CENPI | centromere protein I | 0.0175 | Mitosis |
| 31 | A_24_P342632 | AK5 | adenylate kinase 5, transcript variant 1 | 0.0208 | Brain metabolism |
| 32 | A_33_P3298387 | PLK1 | polo-like kinase 1 | 0.0220 | Cell Cycle |
| 33 | A_19_P00808586 | LOC152742 | uncharacterized LOC152742 | 0.0237 | unknown |
| 34 | A_23_P161218 | ANKRD1 | ankyrin repeat domain 1 | 0.0245 | Endothelial inflamm, migration |
| 35 | A_24_P327181 | WNK4 | WNK lysine deficient protein kinase 4 | 0.0248 | Proliferation / Survival |
| 36 | A_23_P121637 | PRSS12 | protease, serine, 12 | 0.0259 | Plasticity |
| 37 | A_33_P3339361 | ARHGAP11A | Rho GTPase activating protein 11A, xscript var 2 | 0.0266 | Activated in iPS |
| 38 | A_33_P3307253 | AK5 | adenylate kinase 5, transcript variant 2 | 0.0294 | Brain metabolism |
| 39 | A_23_P7727 | HAPLN1 | hyaluronan and proteoglycan link protein 1 | 0.0294 | Cell adhesion |
| 40 | A_33_P3387524 | DEPDC1 | DEP domain containing 1 | 0.0301 | Cell survival |
| 41 | A_33_P3340468 | CENPI | centromere protein I | 0.0312 | Mitosis |
| 42 | A_33_P3376116 | SPC24 | SPC24, NDC80 kinetochore complex component, homolog | 0.0316 | Mitosis |
| 43 | A_23_P126212 | CLSPN | claspin | 0.0316 | DNA repair/ Mitosis |
| 44 | A_33_P3672756 |  | PREDICTED: hypothetical LOC284561 | 0.0358 | antisense RNA |
| 45 | A_33_P3343250 | GPR1 | G protein-coupled receptor 1 | 0.0360 | Inflammation/ chemotactant |
| 46 | A_23_P155711 | NEIL3 | nei endonuclease VIII-like 3 | 0.0374 | DNA repair/ Mitosis |
| 47 | A_33_P3411907 | FGF5 | fibroblast growth factor 5 | 0.0393 | Cell Cycle |
| 48 | A_23_P38271 | MYH2 | myosin, heavy chain 2, skeletal muscle, adult | 0.0396 | Actin Remodeling |
| 49 | A_33_P3218832 | RIMS1 | regulating synaptic membrane exocytosis 1 | 0.0427 | NT release |
| 50 | A_23_P35871 | E2F8 | E2F transcription factor 8 | 0.0452 | Cell Cycle |
| 51 | A_33_P3374205 | MKI67 | antigen identified by monoclonal antibody Ki-67 | 0.0498 | Proliferation |
