# Supplementary material for: Awakened by Cellular Stress: Isolation and Characterization of a Novel Population of Pluripotent Stem Cells Derived from Human Adipose Tissue
Source: PLoS One. 2013 Jun 5;8(6):e64752. doi: 10.1371/journal.pone.0064752 (PMC3673968; doi:10.1371/journal.pone.0064752)
Supplement: Table S6 — Genes related to DNA stability. (DOC) [file pone.0064752.s006.doc]

| **Supplemental Table 6. Genes related to DNA stability** | | | | |  |
| --- | --- | --- | --- | --- | --- |
| **A) DNA-repair genes that are upregulated in Muse-ATs vs. ASCs** | | | | |  |
| ProbeName | Gene Symbol | Gene Name | | Pval |  |
| A_24_P54131 | DCLRE1B | DNA cross-link repair 1B | | 0.007183 |  |
| A_23_P117225 | ERCC5 | excision repair cross-complementing rodent repair deficiency, complementation group 5 | | 0.007947 |  |
| A_32_P95914 | C6orf167 | MMS22-like, DNA repair protein | | 0.008321 |  |
| A_23_P1292 | ERCC6 | excision repair cross-complementing rodent repair deficiency, complementation group 6 | | 0.009173 |  |
| A_24_P83678 | C6orf167 | MMS22-like, DNA repair protein | | 0.010651 |  |
| A_23_P130488 | ERCC2 | excision repair cross-complementing rodent repair deficiency, complementation group 2 | | 0.010807 |  |
| A_23_P23221 | GADD45A | growth arrest and DNA-damage-inducible, alpha | | 0.011135 |  |
| A_19_P00806947 | PCNA | proliferating cell nuclear antigen | | 0.016807 |  |
| A_33_P3252394 | GADD45G | growth arrest and DNA-damage-inducible, gamma | | 0.018317 |  |
| A_24_P120934 | GADD45G | growth arrest and DNA-damage-inducible, gamma | | 0.023703 |  |
| A_23_P216355 | NFKBIL2 | tonsoku-like, DNA repair protein | | 0.025060 |  |
| A_23_P39402 | ALKBH6 | alkB, alkylation repair homolog 6 (E. coli) | | 0.028503 |  |
| A_33_P3241190 | XRCC2 | X-ray repair complementing defective repair in Chinese hamster cells 2 | | 0.030130 |  |
| A_33_P3258612 | PCNA | proliferating cell nuclear antigen | | 0.031406 |  |
| A_23_P28886 | PCNA | proliferating cell nuclear antigen | | 0.033924 |  |
| A_23_P96325 | ERCC6L | excision repair cross-complementing rodent repair deficiency, complementation group 6-like | | 0.036486 |  |
| A_23_P35989 | ALKBH3 | alkB, alkylation repair homolog 3 (E. coli) | | 0.042099 |  |
| A_33_P3301524 | XRCC3 | X-ray repair complementing defective repair in Chinese hamster cells 3 | | 0.045211 |  |
|  | | | | |  |
|  | | | | |  |
|  | | | | |  |
| **B) ABC-cassette genes that are upregulated in Muse-ATs vs. ASCs** | | | | |  |
| ProbeName | Gene Symbol | Gene Name | | Pval |  |
| A_23_P219013 | ABCC10 | ATP-binding cassette, sub-family C (CFTR/MRP), member 10 | | 0.000987 |  |
| A_33_P3422897 | ABCA1 | ATP-binding cassette, sub-family A (ABC1), member 1 | | 0.002002 |  |
| A_23_P89422 | ABCA10 | ATP-binding cassette, sub-family A (ABC1), member 10 | | 0.010420 |  |
| A_33_P3385266 | ABCC6 | ATP-binding cassette, sub-family C (CFTR/MRP), member 6 | | 0.014926 |  |
| A_23_P140876 | ABCA3 | ATP-binding cassette, sub-family A (ABC1), member 3 | | 0.024829 |  |
| A_33_P3292179 | ABCA9 | ATP-binding cassette, sub-family A (ABC1), member 9 | | 0.036646 |  |
| A_32_P23624 | ABCB10 | ATP-binding cassette, sub-family B (MDR/TAP), member 10 | | 0.041847 |  |
| A_23_P171258 | ABCB7 | ATP-binding cassette, sub-family B (MDR/TAP), member 7 | | 0.047579 |  |
|  |  |  | |  |  |
|  |  |  | |  |  |
|  |  |  | |  |  |
|  |  |  | |  |  |
|  |  |  | |  |  |
| **C) Connexin / Gap Junction genes that are present in Muse-AT cells** | | | | |  |
| ProbeName | Gene Symbol | Gene Name | Pval | |  |
| A_23_P204947 | GJB2 | gap junction protein, beta 2, 26kDa | 0.006616 | |  |
| A_23_P1083 | GJA4 | gap junction protein, alpha 4, 37kDa | 0.020058 | |  |
| A_33_P3388397 | GJB4 | gap junction protein, beta 4, 30.3kDa | 0.030993 | |  |
| A_33_P3376379 | Clof17 | consortin, connexin sorting protein (CNST) | 0.048288 | |  |
